# Rest-activity rhythms and cardiovascular events in cardiovascular–kidney–metabolic syndrome: evidence from two nationwide cohorts

**DOI:** 10.1016/j.ajpc.2026.101414

**Published:** 2026-01-10

**Authors:** Bingtao Weng, Haizhen Chen, Han Chen, Ningjian Wang, Hongliang Feng, Kehua Yang, Xiao Tan

**Affiliations:** a Department of Psychiatry, Sir Run Shaw Hospital and Department of Big Data in Health Science, School of Public Health, Zhejiang University School of Medicine, Hangzhou, China; b Department of Endocrinology and Metabolism, Shanghai Gongli Hospital, Shanghai University of Medicine & Health Sciences, Shanghai, China; c Center for Sleep and Circadian Medicine, The Affiliated Brain Hospital, Guangzhou Medical University, Guangzhou, China; d Nursing Department of Psychiatry, Sir Run Shaw Hospital, Zhejiang University School of Medicine, Hangzhou, China; e Department of Clinical Neuroscience, Karolinska Institutet, Stockholm, Sweden

**Keywords:** Cardiovascular–kidney–metabolic syndrome, Circadian rest-activity rhythm, Cardiovascular incidence and mortality, Inflammation, Risk stratification, Incremental predictive model

## Abstract

**Background:**

Circadian rest–activity rhythm (CRAR) is a modifiable determinant of metabolic and cardiovascular health, yet its role in cardiovascular events and mortality among individuals with cardiovascular–kidney–metabolic (CKM) syndrome remains unclear.

**Methods:**

Accelerometer-derived CRAR parameters were analyzed in two nationally representative cohorts. Primary outcomes included cardiovascular incidence among participants with CKM stages 0–3 and all-cause and cardiovascular mortality among those with stages 1–4. Multinomial logistic and Cox proportional hazards models assessed associations of CRAR with CKM progression and subsequent outcomes. Mediation analyses examined inflammatory biomarkers, and improvements in prediction were evaluated using changes in C-statistics.

**Results:**

Among 74,777 participants, higher relative amplitude (RA) tertiles were associated with slower CKM progression and lower risks of cardiovascular incidence (T2: HR 0.87, 95% CI 0.82–0.93; T3: HR 0.79, 95% CI 0.73–0.85), all-cause mortality (T2: HR 0.70, 95% CI 0.64–0.77; T3: HR 0.60, 95% CI 0.54–0.67), and cardiovascular mortality (T2: HR 0.70, 95% CI 0.57–0.86; T3: HR 0.45, 95% CI 0.34–0.61). Higher intradaily variability (IV) was associated with increased all-cause mortality (T2: HR 1.12, 95% CI 1.02–1.22; T3: HR 1.19, 95% CI 1.08–1.30). Inflammatory biomarkers modestly mediated these associations (1%–5%). Optimal thresholds were RA = 0.87 for cardiovascular incidence, RA = 0.81 and IV = 0.68 for mortality. Adding CRAR to basic models improved prediction of all-cause and cardiovascular mortality (ΔC-statistic = 0.019 and 0.017). Results were validated in an independent cohort of 6046 participants.

**Conclusion:**

Adverse CRAR is associated with CKM progression and elevated risks of cardiovascular events and mortality, highlighting its utility in identifying high-risk individuals and guiding targeted interventions through risk stratification and incremental prediction.

## Highlights

• Using two large cohorts, associations between CRAR patterns and CKM progression were analyzed.
• Adverse CRAR patterns may raise cardiovascular and mortality risks, possibly via inflammatory pathways.
• Incorporating CRAR parameters may enhance predictive performance and effectively identify high-risk individuals.
• CRAR may play an important role in preventing cardiovascular events in early CKM (0–3) and in reducing mortality in advanced CKM (1–4).

## Graphical abstract

**Figure:**
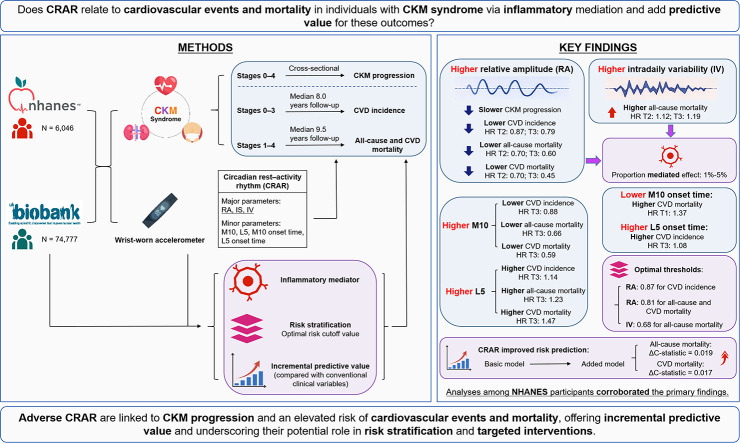

## 1 Introduction

According to the American Heart Association (AHA), cardiovascular-kidney-metabolic (CKM) syndrome is a systemic and progressive condition marked by the coexistence of metabolic disorders, chronic kidney disease (CKD), and cardiovascular disease (CVD), with stages defined from 0 (no risk factors) to 4 (clinical CVD) [1]. The interactions and shared pathological mechanisms among these conditions substantially heighten the risk of adverse CVD outcomes and mortality. From 2015 to 2020, over one-quarter of U.S. adults were estimated to have CKM, accounting for >75 % of total healthcare expenditures [2,3], and by 2021, nearly 30 % of all-cause deaths were attributable to CVD [4]. Given the substantial burden of CKM, prioritizing early prevention of CVD events in CKM stages 0–3 and implementing strategies to reduce mortality risk in stages 1–4 are imperative. Considering the progressive nature of CKM stages, identifying factors that influence stage progression is critical for the early prevention of metabolic disorders, CVD, and related adverse outcomes.

Circadian rest-activity rhythm (CRAR), reflecting an individual’s 24-hour cycle of activity and rest, plays a crucial role in optimizing internal physiological functions and maintaining energy balance [5,6]. Technological advances have facilitated the widespread use of continuous accelerometer recordings to quantify CRAR in free-living settings [[7], [8], [9]]. Owing to factors such as social jet lag, light pollution and excessive use of electronic devices (e.g., mobile phones), the prevalence of CRAR disruptions has surged in recent years [[10], [11], [12]]. In addition, these disruptions have been closely linked to adverse health outcomes, including metabolic dysfunction and diabetes [13,14]. Consequently, a comprehensive evaluation of the associations between CRAR, metabolic disorders, and the risk of CVD incidence and mortality is of paramount importance. Although prior studies have linked CRAR to CVD, hypertension, and mortality [15,16], they are limited by study populations, incomplete consideration of metabolic factors, and unclear underlying mechanisms. Furthermore, disruptions in CRAR have been linked to elevated leukocyte inflammatory markers, and inflammation is well established as a major risk factor for CVD [17,18]. Given that CKM syndrome encompasses metabolic disorders, CKD, and subclinical CVD, it is crucial to investigate the relationships between CRAR, CKM, and subsequent CVD, as well as to determine whether inflammation serves as a mediating factor.

This study leveraged two large-scale databases—the UK Biobank (UKB) and the U.S. National Health and Nutrition Examination Survey (NHANES)—to comprehensively investigate, among individuals with CKM: (1) the relationships between objectively measured CRAR and CKM stages; (2) the associations of CRAR with CVD incidence and mortality; (3) the potential mediation of these associations by inflammatory biomarkers; and (4) the incremental predictive value of CRAR for these outcomes. We hypothesized that, in individuals with CKM, CRAR are associated with CVD incidence, all-cause and CVD mortality through mediation by inflammatory biomarkers and confer additional predictive value for these outcomes.

## 2 Methods

### 2.1 Study design and population

In this prospective cohort study, the UKB was designated as the primary discovery cohort, and NHANES served as the external validation cohort. The UKB and NHANES were approved by the Ethics Review Committees of the National Health Service North West Multicenter (16/NW/0274) and the National Center for Health Statistics (NCHS), respectively, and all participants provided informed consent. This study followed the Strengthening the Reporting of Observational Studies in Epidemiology guideline (*Table S1*). Comprehensive information regarding NHANES participants and the study design can be found in the *Supplementary Methods*.

The UKB collected valid 7-day accelerometer data from 103,594 participants using the Axivity AX3 wrist-worn device between 2013 and 2015 [19], while NHANES obtained data from 14,256 participants using the ActiGraph GT3X+ device across 2011–2014 [20]. Data processing and quality control procedures have been detailed previously and summarized in the *Supplementary Methods* [9,21]. After excluding participants with unreliable accelerometer data, missing CKM or inflammation information, pregnancy, or incomplete follow-up, the final analytical samples included 74,777 UKB and 6046 NHANES participants (*Figures S1–S2*).

### 2.2 Assessment of CRAR parameters

CRAR parameters were derived nonparametrically from activity counts using the R package "nparACT" (version 0.8) [9,21]. The major CRAR parameters in this study include: 1) relative amplitude (RA), calculated as (M10 - L5) / (M10 + L5), reflects the robustness of the 24-hour rhythm; 2) interdaily stability (IS), assessing the regularity of activity patterns across days; and 3) intradaily variability (IV), measuring the fragmentation of the CRAR. Minor CRAR parameters include: 1) the most active 10-h period (M10), the activity counts during the most active 10 h of the day, indicating physical activity levels; 2) the least active 5-h period (L5), the activity counts during the least active 5 h, which captures rest or wakefulness during sleep periods; 3) M10 onset time, where a lower value indicates an earlier wake-up time; and 4) L5 onset time, where a lower value indicates an earlier sleep onset. Details of CRAR parameters are provided in *Table S2*.

### 2.3 Definitions of CKM stages

As defined in the 2023 AHA Presidential Advisory on CKM Health, CKM syndrome encompasses three components: CVD, kidney diseases, and metabolic disorders, classified into stages 0–4 [22]. Details of CKM conditions and staging criteria are provided in *Tables S3–S4*. The predicted 10-year CVD risk for subclinical CVD was estimated based on the AHA PREVENT risk model with the R package "preventr" (version 0.11) [23]. Kidney function was classified according to the "Kidney Disease: Improving Global Outcomes (KDIGO)" guidelines [24]. This study examined CKM stages in relation to various follow-up outcomes: stage 0 indicates no CKM risk factors, and stage 4 indicates clinical CVD. For incidence outcomes, we focused on stages 0–3 to emphasize early prevention; for mortality outcomes, we concentrated on stages 1–4 to guide later-stage interventions.

### 2.4 Ascertainment of incidence and mortality outcomes

For UKB participants, outcomes included CVD incidence, all-cause mortality, and CVD mortality. For NHANES participants, only all-cause and CVD mortality were analyzed due to its cross-sectional design. The incidence and causes of death in this study were determined according to the International Classification of Diseases, 10th Edition (ICD-10) (*Table S5*).

For the UKB cohort, incidence outcomes were ascertained hospital, primary care, and death records, with hospital data updated through October 31, 2022 (England), August 31, 2022 (Scotland), and May 31, 2022 (Wales). Death data were available through May 31, 2024 (England/Wales) and December 31, 2023 (Scotland). Person-years of follow-up were calculated from accelerometer wear to diagnosis, death, or the end of the follow-up. In the NHANES, mortality outcomes were identified using National Death Index (NDI) records through December 31, 2019, with person-months calculated from laboratory assessment to death or follow-up end, due to the unavailability of the exact accelerometer start date.

### 2.5 Covariates and mediators

Analyses were controlled for demographic characteristics including age, sex, race, education level, and Townsend deprivation index (TDI)/poverty income ratio (PIR) (in UKB/in NHANES); behavioral lifestyle including body mass index (BMI), smoking status, drinking status, healthy diet score (only in UKB), and shift work (only in UKB); and chronic disease history including hypertension, diabetes, and hyperlipidemia. Accelerometer-related variables such as moderate to vigorous physical activity (MVPA), sleep duration, sleep efficiency, and season of accelerometer wear were also adjusted. Covariates are detailed in *Table S6*, and missing data were multiply imputed using the R package "mice" (*Table S7*).

Based on previous studies, we selected neutrophil-to-lymphocyte ratio (NLR), systemic inflammation response index (SIRI), acute inflammation severity index (AISI), red cell distribution width (RDW), albumin, and the red cell distribution width to albumin ratio (RAR) as inflammatory biomarkers, with calculation methods reported previously [25,26].

### 2.6 Statistical analysis

The UKB cohort was used for primary analyses, with NHANES for external validation. Continuous variables were described by median and interquartile range (IQR) due to non-normal distribution and compared using the Kruskal-Wallis rank-sum test. while categorical variables were presented as counts and percentages and compared using the Pearson Chi-square test. All continuous CRAR parameters were divided into tertiles (T1–T3), with grouped CRAR as the primary measure and continuous CRAR as supplementary. CKM stages were treated as ordinal, but violation of the proportional odds assumption led to using multinomial logistic regression instead of ordinal logistic regression to assess associations with major CRAR parameters. Earlier CKM stages were used as the reference to examine the relationship between CRAR and CKM progression.

Since participants in the incidence and mortality analyses differed by CKM stages, the two outcomes were analyzed separately using consistent methods. First, Fisher’s exact test was used to assess the distribution of incidence/mortality outcomes across tertiles of the major CRAR parameters and CKM stages. Second, Kaplan-Meier (K-M) survival curves were plotted to depict cumulative incidence/mortality by the major CRAR parameters, with group differences evaluated via log-rank tests. Third, restricted cubic spline (RCS) analyses were conducted to visualize potential nonlinear relationships, with knots selected based on the minimum Bayesian Information Criterion (BIC). Fourth, multivariable Cox proportional hazards models were fitted to estimate hazard ratios (HR) and 95 % confidence intervals (CI) for incidence/mortality outcomes across CRAR tertiles, with the T1 group as the reference. Analyses were adjusted for age, sex, race, education level, TDI, BMI, smoking status, alcohol status, healthy diet score, shift work, hypertension, diabetes, hyperlipidemia, MVPA, sleep duration, sleep efficiency, and season of accelerometer wear. The proportional hazards assumption was evaluated using the Schoenfeld residuals method, with no apparent violations observed. Fifth, the R package "survminer" was applied to identify optimal cutoff points for risk stratification based on maximally selected rank statistics method [27], followed by segmented Cox models to assess the associations between the major CRAR parameters and incidence/mortality outcomes. Associations between CRAR and the incidence of five CVD subtypes—heart failure, coronary heart disease (CHD), atrial fibrillation, peripheral artery disease (PAD), and stroke—were also examined.

Subsequently, exploratory mediation analyses were performed using the R package "CMAverse" for major CRAR parameters that were significantly associated with CVD incidence and mortality, to assess whether specific inflammatory biomarkers mediated these relationships in CKM participants, as detailed in the *Supplementary Methods*. All biomarkers were log-transformed and standardized prior to analysis. Subgroup analyses were further conducted to evaluate potential effect modification by age (< 65 vs. ≥ 65 years), sex, and CKM staging group (stages 0–1 vs. 2–3 for incidence; stages 1–2 vs. 3–4 for mortality). Additionally, six sensitivity analyses were performed: 1) additionally adjusting for estimated glomerular filtration rate (eGFR), depression, and cancer; 2) excluding participants with events or died within the first two years of follow-up; 3) applying the Fine-Gray model to account for competing risk of CVD death; 4) assessing four minor CRAR parameters to complement the main findings; 5) further exploring the mediating role of C-reactive protein (CRP) in the association between adverse CRAR and CVD incidence and mortality; and 6) replicating RCS and multivariable Cox analyses in the NHANES CKM population to externally validate the robustness of results from the UKB cohort.

Finally, the R package "survIDINRI" was used to calculate the C-statistic, continuous net reclassification improvement (NRI), and integrated discrimination improvement (IDI). Receiver operating characteristic (ROC) curves were generated, and changes in the C-statistic and area under the curve (AUC) were compared to assess the incremental predictive value of incorporating CRAR parameters into the basic model for incidence/mortality outcomes in the CKM population. Furthermore, likelihood ratio tests were conducted to evaluate improvements in model fit, and decision curve analysis (DCA) was applied to determine the clinical utility of the incremental models. Prior to model construction, the dataset was randomly split 7:3 into training and testing sets, key features were identified using the Adaptive Best Subset Selection (ABESS) and Boruta algorithm [28,29], and variables with variance inflation factor (VIF) > 10 were excluded to address multicollinearity.

The mediation analyses of inflammatory biomarkers, risk stratification, and the evaluation of the incremental predictive value of CRAR parameters were exploratory, providing supplementary evidence on potential mechanisms and predictive relevance for cardiovascular events in the CKM population. All statistical analyses were conducted using R software (version 4.5.1), with a two-tailed *P* value of < 0.050 considered statistically significant.

## 3 Results

### 3.1 Baseline characteristics

A total of 74,777 eligible UKB participants were included in primary analysis (median [IQR] age: 62.0 [55.0–68.0] years; 44.8 % male, median [IQR] follow-up: 8.0 [7.4–8.5] years for incidence outcomes and 9.5 [9.0–10.0] years for mortality outcomes), including 8944 participants at CKM stage 0 and 9720 at CKM stage 4 or with CVD diagnosed at baseline. Baseline characteristics by RA tertiles are presented in Table 1. Participants in higher RA tertiles were younger, had lower TDI and BMI, showed lower prevalences of hypertension, diabetes, hyperlipidemia, depression, and cancer, reported less smoking but more drinking, and demonstrated longer sleep duration, higher sleep efficiency, and greater MVPA (all *P* < 0.001). Moreover, with increasing RA tertiles, the proportion of individuals at advanced CKM stages (3–4) decreased, whereas that at CKM stage 0 increased. Additionally, the baseline characteristics of participants in the UKB and NHANES stratified by CKM staging are provided in *Tables S8–S9*, respectively.

**Table 1:** Baseline characteristics of participants from the UKB categorized by RA tertiles a.

**Characteristics**	**Overall**	**Categories of RA**			***P***^b^
		**Tertile 1**	**Tertile 2**	**Tertile 3**	
**No. of participants**	74,777	24,927 (33.3)	24,922 (33.3)	24,928 (33.3)	
**Age (years)**	62.0 (55.0, 68.0)	63.0 (55.0, 68.0)	63.0 (55.0, 68.0)	62.0 (55.0, 67.0)	0.001
**Sex**					>0.999
Male	33,524 (44.8)	11,175 (44.8)	11,173 (44.8)	11,176 (44.8)	
Female	41,253 (55.2)	13,752 (55.2)	13,749 (55.2)	13,752 (55.2)	
**Race/Ethnicity**					<0.001
White	72,563 (97.0)	23,774 (95.4)	24,279 (97.4)	24,510 (98.3)	
Non-White	2214 (3.0)	1153 (4.6)	643 (2.6)	418 (1.7)	
**Education level**					0.027
College or above	17,248 (23.1)	5729 (23.0)	5653 (22.7)	5866 (23.5)	
High school or equivalent	4637 (6.2)	1611 (6.5)	1554 (6.2)	1472 (5.9)	
Less than high school	52,892 (70.7)	17,587 (70.6)	17,715 (71.1)	17,590 (70.6)	
**TDI**	−2.5 (−3.8, −0.2)	−2.1 (−3.6, 0.6)	−2.6 (−3.9, −0.4)	−2.7 (−4.0, −0.8)	<0.001
**BMI (kg/m^2^)**	26.0 (23.6, 29.0)	27.4 (24.6, 30.8)	26.1 (23.7, 28.8)	25.0 (22.9, 27.4)	<0.001
**Smoking status**					<0.001
Never smoking	42,697 (57.1)	13,481 (54.1)	14,293 (57.4)	14,923 (59.9)	
Previous smoking	26,921 (36.0)	8991 (36.1)	9048 (36.3)	8882 (35.6)	
Current smoking	5159 (6.9)	2455 (9.8)	1581 (6.3)	1123 (4.5)	
**Drinking status**					<0.001
No	4065 (5.4)	1760 (7.1)	1186 (4.8)	1119 (4.5)	
Yes	70,712 (94.6)	23,167 (92.9)	23,736 (95.2)	23,809 (95.5)	
**Healthy diet score**	3.0 (2.0, 4.0)	3.0 (2.0, 4.0)	3.0 (2.0, 4.0)	3.0 (2.0, 4.0)	<0.001
**Shift work**					<0.001
No	68,727 (91.9)	22,413 (89.9)	23,169 (93.0)	23,145 (92.8)	
Yes	6050 (8.1)	2514 (10.1)	1753 (7.0)	1783 (7.2)	
**Hypertension**					<0.001
No	19,845 (26.5)	5791 (23.2)	6553 (26.3)	7501 (30.1)	
Yes	54,932 (73.5)	19,136 (76.8)	18,369 (73.7)	17,427 (69.9)	
**Diabetes**					<0.001
No	72,303 (96.7)	23,507 (94.3)	24,248 (97.3)	24,548 (98.5)	
Yes	2474 (3.3)	1420 (5.7)	674 (2.7)	380 (1.5)	
**Hyperlipidemia**					<0.001
No	10,586 (14.2)	3091 (12.4)	3500 (14.0)	3995 (16.0)	
Yes	64,191 (85.8)	21,836 (87.6)	21,422 (86.0)	20,933 (84.0)	
**Depression**					<0.001
No	68,439 (91.5)	22,279 (89.4)	22,930 (92.0)	23,230 (93.2)	
Yes	6338 (8.5)	2648 (10.6)	1992 (8.0)	1698 (6.8)	
**Cancer**					<0.001
No	67,803 (90.7)	22,466 (90.1)	22,600 (90.7)	22,737 (91.2)	
Yes	6974 (9.3)	2461 (9.9)	2322 (9.3)	2191 (8.8)	
**eGFR (mL/min/1.73m^2^)**	92.5 (83.1, 101.1)	90.8 (81.2, 100.0)	92.5 (83.3, 101.2)	94.0 (85.1, 101.9)	<0.001
**MVPA (minutes/week)**	115.3 (53.7, 213.0)	59.5 (25.7, 118.3)	108.7 (58.5, 181.8)	207.8 (121.2, 331.3)	<0.001
**Sleep duration (hours/day)**	7.3 (6.8, 7.9)	7.1 (6.5, 7.7)	7.4 (6.8, 7.9)	7.5 (7.0, 8.0)	<0.001
**Sleep efficiency (%)**	76.7 (71.9, 81.0)	73.8 (68.6, 78.5)	77.0 (72.7, 81.0)	78.8 (74.6, 82.7)	<0.001
**Season of accelerometer wear**					<0.001
Spring	16,849 (22.5)	5252 (21.1)	5550 (22.3)	6047 (24.3)	
Summer	19,555 (26.2)	6428 (25.8)	6460 (25.9)	6667 (26.7)	
Autumn	22,344 (29.9)	7403 (29.7)	7457 (29.9)	7484 (30.0)	
Winter	16,029 (21.4)	5844 (23.4)	5455 (21.9)	4730 (19.0)	
**CKM stage**					<0.001
Stage 0	8944 (12.0)	2129 (8.5)	2850 (11.4)	3965 (15.9)	
Stage 1	4681 (6.3)	1598 (6.4)	1602 (6.4)	1481 (5.9)	
Stage 2	55,128 (73.7)	18,443 (74.0)	18,526 (74.3)	18,159 (72.8)	
Stage 3	1891 (2.5)	864 (3.5)	609 (2.4)	418 (1.7)	
Stage 4	4133 (5.5)	1893 (7.6)	1335 (5.4)	905 (3.6)	

a Given the non-normal distribution of continuous variables, these are presented as median (interquartile range), while categorical variables are expressed as n ( %). All values are rounded to one decimal place for precision.

b Due to the non-normal distribution, the Kruskal-Wallis rank sum test was used for continuous variables, while the Pearson's Chi-squared test was applied to categorical variables. **Abbreviations:** UKB, UK Biobank; RA, relative amplitude; TDI, Townsend deprivation index; BMI, body mass index; eGFR, estimated glomerular filtration rate; MVPA, moderate to vigorous physical activity; CKM, cardiovascular-kidney-metabolic syndrom.

### 3.2 Relationship between CRAR and CKM staging

**Fig. 1:**
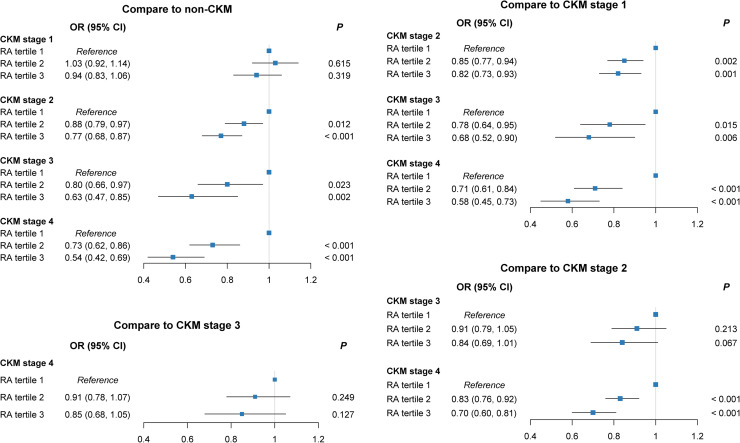
**Association between RA tertiles and CKM staging (stages 0–4).***P* values from multinomial logistic regression models adjusted for age, sex, race, education, TDI, BMI, smoking status, drinking status, healthy diet score, shift work, SBP, HbA1c, total cholesterol, MVPA, sleep duration, sleep efficiency, and season of accelerometer wear. **Abbreviations:** RA, relative amplitude; CKM, cardiovascular-kidney-metabolic syndrome; RA, relative amplitude; OR, odds ratio; CI, confidence interval; TDI, Townsend deprivation index; BMI, body mass index; SBP, systolic blood pressure; HbA1c, glycated hemoglobin; MVPA, moderate to vigorous physical activity.

As shown in Fig. 1, compared with T1 RA groups, higher RA tertiles were associated with a lower likelihood of progressing from earlier to advanced CKM stages. Specifically, in T2 RA groups, compared with the non-CKM group, the risks of progression to CKM stages 2–4 were reduced (odds ratio [OR] = 0.88, 95 % confidence interval [CI]: 0.79–0.97, *P* = 0.012; OR = 0.80, 95 % CI: 0.66–0.97, *P* = 0.023; OR = 0.73, 95 % CI: 0.62–0.86, *P* < 0.001, respectively), with even greater reductions observed in T3 RA groups. Similar patterns were noted when CKM stage 1 or 2 served as the reference, while no protective effect was observed when comparing stage 3 with 4 (all *P* > 0.050). For IV, only participants in T3 group showed a higher risk of progression from CKM stages 0–2 to stage 4 (*P* < 0.050), while IS was not associated with CKM progression (all *P* > 0.050) (*Figures S3–S4*).

### 3.3 Associations between CRAR and incidence outcomes in individuals with CKM stages 0–3

As shown in Table 2, after adjustment for all covariates, participants in T2 and T3 RA groups exhibited significantly lower CVD incidence risks compared with T1 group (HR = 0.87, 95 % CI: 0.82–0.93, *P* < 0.001; HR = 0.79, 95 % CI: 0.73–0.85, *P* < 0.001, respectively), with a significant decreasing trend across RA tertiles (*P* for trend < 0.001). In contrast, IS and IV showed no significant associations with CVD incidence (all *P* > 0.050). Associations of RA, IS, and IV with the risks of five CVD subtypes are shown in *Figure S5. Figure S6* and *Table S10* indicate that only RA enabled meaningful risk stratification: among participants with CKM stages 0–3, those with RA ≥ 0.87 exhibited significantly lower CVD incidence risk than those with RA < 0.87.

**Table 2:** Associations between major circadian rest-activity rhythm and the risk of CVD incidence in individuals with CKM stages 0–3.

**CRAR**	**Events/PYs (‰)**	**Model 1**			**Model 2**			**Model 3**		
		**HR (95% CI)**	***P***	***P* for trend**^b^	**HR (95% CI)**	***P***	***P* for trend**^b^	**HR (95% CI)**	***P***	***P* for trend**^b^
**RA**										
Continuous a	6777/496,893 (13.64)	0.78 (0.76, 0.81)	< 0.001		0.86 (0.83, 0.89)	< 0.001		0.89 (0.85, 0.92)	< 0.001	
Categories				< 0.001			< 0.001			< 0.001
Tertile 1	2612/156,924 (16.65)	*Reference*	-		*Reference*	-		*Reference*	-	
Tertile 2	2236/166,514 (13.43)	0.79 (0.74, 0.83)	< 0.001		0.86 (0.81, 0.91)	< 0.001		0.87 (0.82, 0.93)	< 0.001	
Tertile 3	1929/173,456 (11.12)	0.65 (0.61, 0.68)	< 0.001		0.75 (0.70, 0.80)	< 0.001		0.79 (0.73, 0.85)	< 0.001	
**IS**										
Continuous a	6777/496,893 (13.64)	1.00 (0.98, 1.03)	0.660		1.00 (0.98, 1.02)	0.811		0.99 (0.97, 1.01)	0.440	
Categories				0.659			0.971			0.684
Tertile 1	2212/166,749 (13.27)	*Reference*	-		*Reference*	-		*Reference*	-	
Tertile 2	2263/165,927 (13.64)	1.00 (0.94, 1.06)	0.960		1.00 (0.94, 1.06)	0.890		0.98 (0.93, 1.04)	0.543	
Tertile 3	2302/164,217 (14.02)	1.01 (0.96, 1.07)	0.653		1.00 (0.94, 1.06)	0.967		0.99 (0.93, 1.05)	0.690	
**IV**										
Continuous a	6777/496,893 (13.64)	0.99 (0.98, 1.00)	0.139		1.00 (0.99, 1.01)	0.517		1.00 (0.99, 1.01)	0.764	
Categories				0.257			0.475			0.625
Tertile 1	2292/165,567 (13.84)	*Reference*	-		*Reference*	-		*Reference*	-	
Tertile 2	2307/165,620 (13.93)	1.01 (0.95, 1.07)	0.796		1.01 (0.95, 1.07)	0.694		1.00 (0.95, 1.06)	0.876	
Tertile 3	2178/165,706 (13.14)	0.97 (0.91, 1.03)	0.278		0.98 (0.92, 1.04)	0.503		0.99 (0.93, 1.05)	0.640	

a Since the continuous variable has a narrow range (1–2), it was scaled by a factor of ten for the analysis. The HR reflects the hazard ratio for a 0.1 unit change in the variable.

b *P* for trend was calculated using multivariable Cox regression models to evaluate the differences in the medians of continuous variables across different tertile groups. Model 1 was adjusted solely for age and sex; Model 2, building upon the covariate adjustments in Model 1, was further adjusted for race, education level, TDI, BMI, smoking status, drinking status, healthy diet score, shift work, hypertension, diabetes, and hyperlipidemia; Model 3, building upon the covariate adjustments in Model 2, was additionally adjusted for MVPA, sleep duration, sleep efficiency, and season of accelerometer wear. **Abbreviations:** CVD, cardiovascular disease; CKM, cardiovascular-kidney-metabolic syndrom; CRAR, circadian rest-activity rhythm; PYs, person-years; HR, hazard ratio; CI, confidence interval; RA, relative amplitude; IS, interdaily stability; IV, intradaily variability; BMI, body mass index; TDI, Townsend deprivation index; MVPA, moderate to vigorous physical activity.

The CVD incidence decreased progressively with higher RA tertiles (*P* < 0.001) and increased with advancing CKM stages (*P* < 0.001), while no significant differences were observed across IS or IV tertiles (*P* = 0.064 and 0.089, respectively) (*Figure S7*). The K-M survival curves (*Figure S8*) revealed that, for CVD incidence, survival probability increased across RA tertiles, decreased across IS tertiles, and for IV, the T2 and T3 groups had similar probabilities, both lower than T1 (all log-rank *P* < 0.001). RA exhibited a significant linear inverse association with the CVD incidence risk (*P* for overall < 0.001, *P* for nonlinear = 0.265), whereas IV demonstrated a significant nonlinear positive association (*P* for overall = 0.006, *P* for nonlinear = 0.002) (*Figure S9*).

### 3.4 Associations between CRAR and mortality outcomes in patients with CKM stages 1–4

**Fig. 2:**
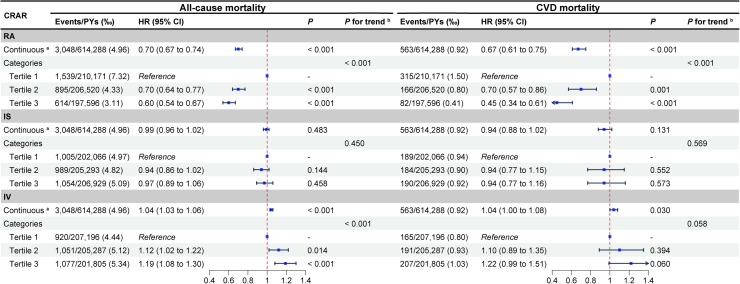
**Associations between major CRAR and the risk of all-cause and CVD mortality in patients with CKM stages 1–4. ^a^** Since the continuous variable has a narrow range (1–2), it was scaled by a factor of ten for the analysis. The HR reflects the hazard ratio for a 0.1 unit change in the variable. **^b^***P* for trend was calculated using multivariable Cox regression models to evaluate the differences in the medians of continuous variables across different tertile groups. **Abbreviations**: CRAR, circadian rest-activity rhythm; RA, relative amplitude; IS, interdaily stability; IV, intradaily variability; CVD, cardiovascular disease; HR, hazard ratio; CI, confidence interval; CKM, cardiovascular-kidney-metabolic syndrome.

As shown in Fig. 2, after adjusting for all covariates, participants in the T2 RA group had significantly lower risks of all-cause and CVD mortality compared withT1 group (HR = 0.70, 95 % CI: 0.64–0.77, *P* < 0.001; HR = 0.70, 95 % CI: 0.57–0.86, *P* = 0.001, respectively). The protective effect was even stronger in T3 RA group (HR = 0.60, 95 % CI: 0.54–0.67; HR = 0.45, 95 % CI: 0.34–0.61, respectively; both *P* < 0.001), with significant decreasing trends across RA tertiles (both *P* for trend < 0.001). In contrast, participants in the T2 and T3 IV groups had a significantly higher risks of all-cause mortality only compared with the T1 group (HR = 1.12, 95 % CI: 1.02–1.22, *P* = 0.014; HR = 1.19, 95 % CI: 1.08–1.30, *P* < 0.001, respectively), with an increasing trend across IV tertiles (*P* for trend < 0.001), whereas IS showed no significant associations with either outcome (all *P* > 0.050). *Figures S10-S11* and *Tables S11-S12* further indicate that both RA and IV provided meaningful risk stratification, with RA showing a cutoff of 0.81 for both outcomes, and IV showing cutoffs of 0.68 for all-cause mortality and 0.76 for CVD mortality (all *P* < 0.050).

The all-cause and CVD mortality decreased progressively with higher RA tertiles (both *P* < 0.001), increased with higher IV tertiles (*P* < 0.001 and *P* = 0.017, respectively), and rose markedly at advanced CKM stages (3–4) (both *P* < 0.001) (*Figures S12–S13*). The K-M survival curves (*Figures S14–S15*) revealed that, for both all-cause and CVD mortality, survival probability increased across RA tertiles (both log-rank *P* < 0.001), whereas survival patterns across IS and IV groups varied by mortality outcomes. RA exhibited significant nonlinear inverse associations with all-cause and CVD mortality (both *P* for overall < 0.001; *P* for nonlinear = 0.037 and 0.009, respectively), whereas IV demonstrated a positive linear association with all-cause mortality only (*P* for overall < 0.001, *P* for nonlinear = 0.682) (*Figures S16–S17*).

### 3.5 Results of mediation, subgroup and sensitivity analyses

**Fig. 3:**
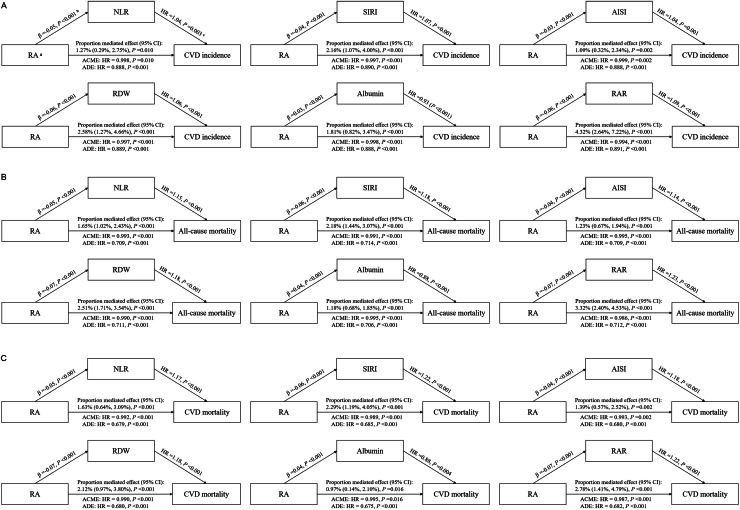
**Mediation analyses of inflammation biomarkers in the associations of RA with CVD incidence, all-cause and CVD mortality in CKM individuals. ^a^** Since the continuous variable has a narrow range (1–2), it was scaled by a factor of ten for the analysis. The HR reflects the hazard ratio for a 0.1 unit change in the variable. **^b^***P* values from multivariable logistic regression models for major CRAR and inflammation biomarkers. **^c^***P* values from multivariable Cox proportional hazards models for inflammation biomarkers and all-cause mortality. (A) CVD incidence, (B) all-cause mortality, (C) CVD mortality. **Abbreviations**: RA, relative amplitude; NLR, neutrophil-to-lymphocyte ratio; SIRI, systemic inflammation response index; AISI, absolute inflammatory score index; RDW, red cell distribution width; RAR, red cell distribution width to albumin ratio; HR, hazard ratio; CI, confidence interval; ACME: average causal mediation effect; ADE: average direct effect; CRAR, circadian rest-activity rhythm; CKM, cardiovascular-kidney-metabolic syndrome.

Inflammatory biomarkers—including NLR, SIRI, AISI, RDW, albumin, RAR, and CRP—partially mediated the associations of RA with CVD incidence, all-cause and CVD mortality (all *P* < 0.050), with mediation proportions ranging from 1 % to 5 %, as shown in Fig. 3 and *Figure S18*. RDW, albumin, and RAR also mediated the link between IV and all-cause mortality (*Figure S19*). Subgroup analyses showed interactions of age with grouped RA/IS on CVD incidence (*Figures S20–S22*).

In sensitivity analyses, results remained consistent after additionally adjusting for eGFR, depression, and cancer, or excluding participants with CVD incidence or deaths in the first two follow-up years (*Figures S23–S24*), and were supported by the Fine-Gray competing risk model for CVD death (*Table S13*). Analyses of minor CRAR parameters showed M10 and L5 were associated with CVD incidence, all-cause and CVD mortality, exhibiting nonlinear trends; T3 L5 onset time group was linked to increased CVD incidence, and M10 onset time was linearly negatively associated with CVD mortality (*Tables S14-S15, Figures S25–S26*). The NHANES results were largely consistent with the UKB findings, supporting the robustness of our results (*Table S16, Figures S27–S28*).

### 3.6 Incremental predictive value of CRAR parameters

**Fig. 4:**
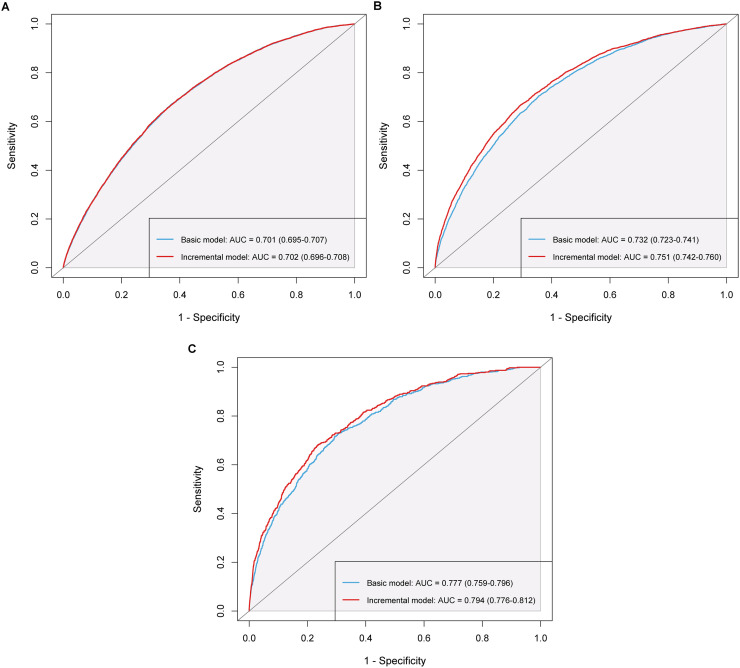
**ROC curves for basic and incremental prediction models of CVD incidence, all-cause and CVD mortality.** (A) CVD incidence, (B) all-cause mortality, (C) CVD mortality. The study population for CVD incidence includes individuals with CKM stages 0–3, while those for all-cause and CVD mortality consist of patients with CKM stages 1–4. **Abbreviations**: ROC, receiver operating characteristic; AUC, area under the curve; CVD, cardiovascular disease; CKM, cardiovascular-kidney-metabolic syndrome.

Table 3 presents the incremental predictive value of CRAR parameters for CVD incidence, all-cause and CVD mortality. Variable selection and VIF values are detailed in *Tables S17-S19* and *Figure S29*. Adding CRAR parameters markedly improved the C-statistic, risk reclassification, and discriminative power of the basic model for all-cause and CVD mortality (ΔC-statistic = 0.019, continuous NRI = 0.120, IDI = 0.016; ΔC-statistic = 0.017, continuous NRI = 0.200, IDI = 0.008, respectively, all *P* < 0.001). Fig. 4 and *Table S20* show that adding CRAR parameters significantly increased the AUC for all-cause and CVD mortality models (ΔAUC = 0.019; ΔAUC = 0.017, respectively, both DeLong *P* < 0.001). *Figure S30* further demonstrates that adding CRAR parameters increases net benefit across thresholds, highlighting their incremental value in predicting all-cause and CVD mortality.

**Table 3:** Evaluation metrics for incremental predictive value of circadian rest-activity rhythm for CVD incidence, all-cause and CVD mortality in individuals with CKM.

	**C-statistic (95% CI)**	**ΔC-statistic (95% CI)**	***P***	**Continuous NRI (95% CI)**	***P***	**IDI (95% CI)**	***P***
**CVD incidence**^a^							
Basic model	0.694 (0.689, 0.700)	*Reference*		*Reference*		*Reference*	
Basic model + CRAR	0.696 (0.690, 0.702)	0.001 (0.001, 0.002)	< 0.001	0.032 (0.015, 0.045)	< 0.001	0.001 (0.001, 0.002)	< 0.001
**All-cause mortality**^b^							
Basic model	0.727 (0.718, 0.735)	*Reference*		*Reference*		*Reference*	
Basic model + CRAR	0.745 (0.737, 0.754)	0.019 (0.015, 0.023)	< 0.001	0.120 (0.100, 0.139)	< 0.001	0.016 (0.013, 0.019)	< 0.001
**CVD mortality**^c^							
Basic model	0.779 (0.761, 0.799)	*Reference*		*Reference*		*Reference*	
Basic model + CRAR	0.797 (0.779, 0.815)	0.017 (0.011, 0.025)	< 0.001	0.200 (0.143, 0.241)	< 0.001	0.008 (0.005, 0.014)	< 0.001

a The population for predicting CVD incidence includes participants in CKM stages 0–3. The basic model incorporates variables including age, sex, BMI, SBP, HbA1c, eGFR, and MVPA. The incremental model builds upon the base model by adding CRAR feature, specifically RA.

b The population for predicting all-cause mortality includes participants in CKM stages 1–4. The basic model incorporates variables including age, sex, TDI, SBP, HbA1c, total cholesterol, eGFR, and cancer. The incremental model builds upon the base model by adding CRAR features, specifically RA, IS, and L5.

c The population for predicting CVD mortality includes participants in CKM stages 1–4. The basic model incorporates variables including age, sex, SBP, HbA1c, and eGFR. The incremental model builds upon the base model by adding CRAR feature, specifically RA and M10 onset time. **Abbreviations:** CVD, cardiovascular disease; CKM, cardiovascular-kidney-metabolic syndrom; CRAR, circadian rest-activity rhythm; CI, confidence interval; NRI, net reclassification improvement; IDI, integrated discrimination improvement; BMI, body mass index; TDI, Townsend deprivation index; SBP, systolic blood pressure; HbA1c, glycated hemoglobin; eGFR, estimated glomerular filtration rate; MVPA, moderate to vigorous physical activity; RA, relative amplitude; IS, interdaily stability; M10, the most active 10-h period; L5, the least active 5-h period.

## 4 Discussion

This study leveraged data from CKM participants in the UKB and NHANES as the main analyses and external validation, respectively, to comprehensively examine the associations of CRAR with CVD incidence, all-cause mortality, and CVD mortality. Higher RA tertiles were associated with lower progression to advanced CKM stages. RCS analyses indicated that, within the CKM population, RA exhibited a linear inverse association with CVD incidence and a nonlinear inverse association with all-cause and CVD mortality, while IV (rhythm fragmentation) was positively associated with all-cause mortality. Higher RA tertiles were linked to better baseline characteristics and lower risks of CVD incidence, all-cause and CVD mortality, whereas higher IV tertiles were associated solely with increased all-cause mortality. Mediation analyses demonstrated that inflammatory biomarkers significantly mediated these associations. Additionally, higher M10 and L5 tertiles were respectively associated with lower and higher risks of CVD incidence and mortality. Optimal thresholds for risk stratification were identified as RA = 0.87 for CVD incidence, RA = 0.81 for all-cause and CVD mortality, and IV = 0.68 for all-cause mortality. Importantly, incorporating CRAR parameters into basic risk models markedly improved their predictive performance for all-cause and CVD mortality.

This study is the first to investigate the associations of CRAR with CVD incidence and mortality among individuals with CKM syndrome, thereby complementing and extending the existing evidence. Previous studies with small to medium sample sizes have reported that higher RA and IS, together with lower IV, were linked to more favorable cardiometabolic profiles [30,31]. Findings from the UKB and NHANES further demonstrated that lower RA and higher IV were associated with elevated risks of CVD incidence and mortality [9,15,16]. Additionally, a cross-sectional study found that better overall sleep quality was associated with lower odds of advanced CKM (stages 3–4) [32], although assessments relied on subjective self-reports. Notably, our study extends these observations to the emerging high-risk CKM population, revealing a dose–response inverse association of objectively measured CRAR robustness (i.e., RA) with CVD incidence, all-cause mortality, and CVD mortality, as well as a positive association of CRAR fragmentation (i.e., IV) with all-cause mortality. Moreover, prior research identified an l-shaped relationship between questionnaire-based physical activity and early CKM risk (stages 1–2), emphasizing the protective effect of moderate-intensity activity, whereas higher-intensity activity might attenuate or reverse these benefits [33]. Our findings provide new evidence from objectively monitored rest-activity rhythms, demonstrating that lower daytime activity intensity (i.e., M10) and higher nighttime movement intensity (i.e., L5) were associated with increased risks of CVD incidence and mortality, while later sleep onset (i.e., L5 onset time) and earlier wake-up time (i.e., M10 onset time) were associated with higher risks of CVD incidence and CVD mortality, respectively. Analyses of these specific activity intensities and temporal patterns offer more precise insights for guiding CVD prevention strategies through CRAR–based interventions.

The associations between unfavorable CRAR and elevated risks of CVD incidence and mortality in individuals with CKM syndrome are likely driven by multiple biological mechanisms. CRAR disruption has been implicated in a variety of conditions, including diabetes, cancer, dementia, and pro-inflammatory states [[34], [35], [36], [37]], which may accelerate biological aging and consequently increase mortality risk. Inflammatory responses can trigger insulin resistance and disrupt glucose and lipid metabolism [38,39], while obesity or metabolic abnormalities in early CKM stages (0–1) may further exacerbate these effects [40], thereby promoting disease progression. The release of inflammatory cytokines and oxidative stress products impairs vascular endothelial homeostasis and drives vascular remodeling. In patients with later CKM (stages 2–3), these changes increase arterial stiffness, elevate the risk of cardiovascular events and end-stage renal disease, and ultimately contribute to higher mortality [41,42]. CRAR disruption has also been associated with elevated leukocyte-based inflammatory indices and increased levels of salivary pro-inflammatory biomarkers [17,43]. Consistent with previous studies, our mediation analyses identified inflammatory markers such as AISI and RAR as potential mediators linking CRAR to CVD incidence and mortality. Furthermore, genetic studies have shown that mutations associated with CRAR disruption (e.g., *FOXJ1, ZFYVE21*) are linked to multiple diseases [44], and *clock* gene have been demonstrated to regulate cardiovascular function [45,46]. Collectively, our findings suggest a potential inflammatory pathway mediating the observed associations, although further studies are warranted to elucidate the underlying mechanisms in CKM populations.

This study further emphasizes the clinical relevance of CRAR in stratifying and predicting CVD incidence and mortality among individuals with CKM syndrome. Specifically, the study determined optimal RA thresholds for CVD incidence, all-cause mortality, and CVD mortality, and an optimal IV threshold for all-cause mortality, offering guidance for identifying high-risk CKM populations and informing targeted interventions. CRAR reflects the integrated effects of rhythmic environmental and behavioral factors, and disrupted CRAR can be mitigated through lifestyle and environmental interventions [47], thereby reducing CVD incidence and mortality risk. Previous studies have indicated that increased physical activity and daylight exposure may improve CRAR in patients with gastrointestinal cancers [48]. A study of an elderly Brazilian cohort indicated that, after accounting for physical activity, CRAR fragmentation and daytime stability were not associated with mortality, highlighting physical activity as a key strategy for promoting population health [49]. In mouse models, high-fat diets have been shown to activate inflammatory pathways, linking them to CRAR disruption [50]. Moreover, combined circadian rhythm and dietary interventions may enhance cardiometabolic function in CVD patients [51]. Generally, these findings underscore the potential benefits of targeted interventions—including controlled light exposure, physical activity, and dietary modifications—for individuals with high-risk CRAR profiles to reduce inflammation and improve clinical outcomes. Additionally, our study demonstrated that incorporating CRAR parameters into basic models—including demographic and hematological indicators—substantially enhanced the prediction of all-cause and CVD mortality. Prior study has also proposed that CRAR parameters may serve as digital biomarkers for predicting type 2 diabetes risk [52]. Given the cost-effectiveness and broad applicability of accelerometer-measured CRAR, it may represent a practical tool for community-level mortality risk screening.

### 4.1 Strengths and limitations

Our study has several strengths, including being the first to examine associations between CRAR, CKM stages, and subsequent CVD outcomes, with analyses spanning incidence in early CKM (stages 0–3) and mortality in advanced CKM (stages 1–4), and the use of CRAR thresholds and predictive models to identify high-risk populations. Two large prospective cohorts with diverse demographic and genetic backgrounds were included, with one serving as the primary analysis cohort and the other as an external validation cohort, providing mutual corroboration and further strengthening the robustness and generalizability of the findings. Nonetheless, several limitations should be noted. First, as an observational study, residual confounding cannot be excluded and causality remains unconfirmed, highlighting the need for interventional and Mendelian randomization studies. Second, most participants were of European ancestry, limiting generalizability and emphasizing the need for multi-ethnic research. Third, CRAR were assessed only at baseline, so temporal changes were not captured. Although CRAR parameters are generally stable among individuals aged 20 to 60 years [21], future studies should adopt repeated measurements to evaluate their dynamic effects. Fourth, although CRAR may serve as a preliminary predictor of all-cause and CVD mortality in CKM, its utility for community-based screening requires further validation. Finally, although this study used two cohorts for cross-validation, potential measurement differences may exist as CRAR parameters were derived from different accelerometers in UKB and NHANES. Previous studies have analyzed accelerometer-measured samples from both cohorts, supporting the comparability of these data [53].

## 5 Conclusions

In the CKM population, adverse CRAR patterns (such as lower RA and higher IV) were associated with CKM progression and may contribute to elevated risks of CVD incidence and mortality, potentially mediated by inflammatory pathways. Incorporating CRAR parameters improved the prediction of these outcomes, and CRAR thresholds effectively identified high-risk individuals. These findings suggest a potential role of CRAR in preventing cardiovascular events in early CKM stages (0–3) and reducing mortality in advanced CKM stages (1–4), providing valuable evidence to inform the development of targeted circadian rhythm–based interventions.

## Data availability

The data that support the findings of this study are available from the website of the UK Biobank at http://ukbiobank.ac.uk/ and website of U.S. National Health and Nutrition Examination Survey at https://cdc.gov/nchs/nhanes/. Statistical code is available on the request by directly contacting the corresponding author (email: xiao.tan@zju.edu.cn).

## Ethics approval statements

This study was conducted in accordance with the Declaration of Helsinki and approved by the appropriate institutional and national research ethics committees. The UK Biobank received ethical approval from the North West Multi-Centre Research Ethics Committee (reference 11/NW/0382). The U.S. National Health and Nutrition Examination Survey (NHANES) was approved by the U.S. NHANES Institutional Review Board and National Center for Health Statistics Research Ethics Review Board (Protocol #2011–17). Informed consent was obtained from each subject in these two cohorts.

## Funding

This research was supported by 10.13039/501100001809National Natural Science Foundation of China (X.T., 82570128), Rut and Arvid Wolff Memorial Foundation (X.T., 2023–02467), and Pioneer R&D Program of Zhejiang Province (X.T., 2025C01119). The funders of the study had no role in study design, data collection, data analysis, data interpretation, writing of the report or involved in the decision to submit the paper for publication.

## CRediT authorship contribution statement

**Bingtao Weng:** Writing – review & editing, Writing – original draft, Visualization, Software, Methodology, Investigation, Formal analysis, Data curation, Conceptualization. **Haizhen Chen:** Writing – review & editing, Methodology, Data curation. **Han Chen:** Writing – review & editing, Methodology. **Ningjian Wang:** Writing – review & editing, Supervision, Methodology. **Hongliang Feng:** Writing – review & editing, Supervision, Resources, Methodology. **Kehua Yang:** Writing – review & editing, Resources, Methodology. **Xiao Tan:** Writing – review & editing, Validation, Supervision, Resources, Project administration, Methodology, Funding acquisition.

## Declaration of competing interest

The authors declare that they have no known competing financial interests or personal relationships that could have appeared to influence the work reported in this paper.

## Appendix Supplementary materials

Image, application 1
