# Supplementary material for: Rest-activity rhythms and cardiovascular events in cardiovascular–kidney–metabolic syndrome: evidence from two nationwide cohorts
Source: Am J Prev Cardiol. 2026 Jan 10;26:101414. doi: 10.1016/j.ajpc.2026.101414 (PMC12830231; doi:10.1016/j.ajpc.2026.101414)
Supplement: Supplementary file 1 [file mmc1.docx]

Rest-activity rhythms and cardiovascular events in cardiovascular–kidney–metabolic syndrome: evidence from two nationwide cohorts

***Supplementary Material***

**Legends**

***Supplementary Methods***

***Supplementary Tables***

**Table S1.** Strengthening the Reporting of Observational Studies in Epidemiology Checklist for this cohort study.

**Table S2.** The meaning of accelerometer-measured circadian rest-activity rhythm parameters.

**Table S3.** Association between IS tertiles and CKM staging (stages 0-4).

**Table S4.** Association between IV tertiles and CKM staging (stages 0-4).

**Table S5.** Identification of outcomes in the UKB and NHANES participants.

**Table S6.** Definitions and descriptions of covariates in the UKB and NHANES participants.

**Table S7.** Proportions of missing value in the UKB and NHANES participants.

**Table S8.** Baseline characteristics of participants from the UKB categorized by CKM stages.

**Table S9.** Baseline characteristics of participants from the NHANES categorized by CKM stages.

**Table S10.** Association between major circadian rest-activity rhythm and the risk of CVD incidence in individuals with CKM stages 0-3 stratified by optimal risk cut-off points.

**Table S11.** Association between major circadian rest-activity rhythm and the risk of all-cause mortality in patients with CKM stages 1-4 stratified by optimal risk cut-off points.

**Table S12.** Association between major circadian rest-activity rhythm and the risk of CVD mortality in patients with CKM stages 1-4 stratified by optimal risk cut-off points.

**Table S13.** Association between the major circadian rest-activity rhythm and the competing risk of CVD mortality in patients with CKM stages 1-4, based on the Fine-Gray model.

**Table S14.** Associations between minor circadian rest-activity rhythm and the risk of CVD incidence in individuals with CKM stages 0-3.

**Table S15.** Associations between minor circadian rest-activity rhythm and the risk of all-cause and CVD mortality in patients with CKM stages 1-4.

**Table S16.** Associations between circadian rest-activity rhythm and the risk of all-cause and CVD mortality in patients with CKM stages 1-4 from the NHANES.

**Table S17.** Multicollinearity evaluation of predictor variables for CVD incidence in individuals with CKM stages 0-3 through variance inflation factor (VIF) analysis.

**Table S18.** Multicollinearity evaluation of predictor variables for all-cause mortality in patients with CKM stages 1-4 through variance inflation factor (VIF) analysis.

**Table S19.** Multicollinearity evaluation of predictor variables for CVD mortality in patients with CKM stages 1-4 through variance inflation factor (VIF) analysis.

**Table S20.** Additional evaluation Metrics for incremental predictive value of circadian rest-activity rhythm for CVD incidence, all-cause and CVD mortality in individuals with CKM.

***Supplementary Figures***

**Figure S1**. Flowchart of participants selection in the UKB cohort.

**Figure S2**. Flowchart of participants selection in the NHANES cohort.

**Figure S3**. Association between IV tertiles and CKM staging (stages 0-4).

**Figure S4**. Association between IS tertiles and CKM staging (stages 0-4).

**Figure S5**. Associations between major CRAR and the risk of incidence of five CVD subtypes in individuals with CKM stages 0-3.

**Figure S6**. Determination of optimal risk stratification cut-off points for major CRAR on CVD incidence in individuals with CKM stages 0-3.

**Figure S7**. Distribution of CVD incidence by major CRAR tertiles and CKM stages in individuals with CKM stages 0-3.

**Figure S8**. Kaplan-Meier survival curves for CVD incidence across major CRAR tertiles in individuals with CKM stages 0-3.

**Figure S9**. Restricted cubic spline analyses for associations between major CRAR and CVD incidence in individuals with CKM stages 0-3.

**Figure S10**. Determination of optimal risk stratification cut-off points for major CRAR on all-cause mortality in patients with CKM stages 1-4.

**Figure S11**. Determination of optimal risk stratification cut-off points for major CRAR on CVD mortality in patients with CKM stages 1-4.

**Figure S12**. Distribution of all-cause mortality by major CRAR tertiles and CKM stages in patients with CKM stages 1-4.

**Figure S13**. Distribution of CVD mortality by major CRAR tertiles and CKM stages in patients with CKM stages 1-4.

**Figure S14**. Kaplan-Meier survival curves for all-cause mortality across major CRAR tertiles in patients with CKM stages 1-4.

**Figure S15**. Kaplan-Meier survival curves for CVD mortality across major CRAR tertiles in patients with CKM stages 1-4.

**Figure S16**. Restricted cubic spline analyses for associations between major CRAR and all-cause mortality in patients with CKM stages 1-4.

**Figure S17**. Restricted cubic spline analyses for associations between major CRAR and CVD mortality in patients with CKM stages 1-4.

**Figure S18**. Mediation analyses of CRP in the associations of RA with CVD incidence, all-cause and CVD mortality in CKM individuals.

**Figure S19**. Mediation analyses of inflammation biomarkers in the associations of IV with all-cause mortality in patients with CKM stages 1-4.

**Figure S20**. Associations between major CRAR and the risk of CVD incidence, all-cause and CVD mortality in CKM individuals, stratified by age.

**Figure S21**. Associations between major CRAR and the risk of CVD incidence, all-cause and CVD mortality in CKM individuals, stratified by sex.

**Figure S22**. Associations between major CRAR and the risk of CVD incidence, all-cause and CVD mortality in CKM individuals, stratified by CKM stages.

**Figure S23**. Associations between major CRAR and the risk of CVD incidence, all-cause and CVD mortality in CKM individuals, further adjusted for eGFR, depression and cancer.

**Figure S24**. Associations between major CRAR and the risk of CVD incidence, all-cause and CVD mortality in CKM individuals, after excluding outcomes occurring within the first two years of follow-up.

**Figure S25**. Restricted cubic spline analyses for associations between minor CRAR and CVD incidence in individuals with CKM stages 0-3.

**Figure S26**. Restricted cubic spline analyses for associations between minor CRAR and all-cause and CVD mortality in patients with CKM stages 1-4.

**Figure S27**. Restricted cubic spline analyses for associations between CRAR and all-cause mortality in patients with CKM stages 1-4 from the NHANES.

**Figure S28**. Restricted cubic spline analyses for associations between CRAR and CVD mortality in patients with CKM stages 1-4 from the NHANES.

**Figure S29**. Feature selection for CVD incidence, all-cause and CVD mortality using the ABESS and Boruta algorithms.

**Figure S30**. Flow chart of participants selection for the change-to-dementia analyses.

***Supplementary Methods***

***Study design for NHANES participants***

This study used data from the 2011–2014 waves of the NHANES as an external validation cohort to corroborate the findings observed in the UKB population. During these waves, all participants aged 6 years and older were asked to wear a wrist-worn accelerometer, the ActiGraph GT3X+ (ActiGraph, Pensacola, FL), continuously for 9 calendar days. According to the study protocol, the first and last days were partial days, yielding up to 7 full days of valid data. NHANES accelerometry data are available at multiple resolutions, including day, hour, and minute levels. For the present study, we used the minute-level MIMS units to derive all accelerometry features included in our models.

Individuals aged 80 and over at the time of interview were excluded as NHANES 2011–2014 topcodes individuals’ age at 80 for privacy reasons. As the NHANES database contains only mortality records, our study population was restricted to participants at CKM stages 1–4. Among participants meeting the inclusion criteria, a total of 5,677 individuals (aged 30–80 years; 38,283.4 person-years of follow-up; 438 deaths) had both wrist accelerometry data that met the specified quality criteria and complete data on a set of traditional risk factors. Survey weights for the combined 2011–2012 and 2013–2014 waves were calculated using the 2-year examination weights divided by 2 as suggested by the NHANES analytic guidelines.

***Quality control of accelerometer measurement data***

UKB participants with unreliable accelerometer data will be excluded if any of the following conditions are met: 1) unexpectedly small or large size; 2) less than 72 hours of data or failure to provide data for all 1-hour periods within a 24-hour cycle during the 7-day data collection period; 3) poor calibration; 4) recalibration using the previous accelerometer record from the same device worn by a different participant; 5) data with a non-zero count of interrupted recording periods; and 6) more than 768 data recording errors (Q3 + 1.5 × IQR).

For NHANES accelerometer measurement data, data quality control procedures were applied, and the tri-axial accelerometer values for the corresponding time periods were set to NA if any of the following conditions were met: 1) poor data quality, indicated by a letter value in the per-minute data quality review flag (PAXFLGSM) variable; 2) non-wear state, indicated by a value of '3' in the estimated wake/sleep/wear state (PAXPREDM) variable for a given minute; 3) inability to compute activity values, indicated by a value of '-0.01' in the per-minute tri-axial activity value (PAXMTSM) variable. Missing activity data were subsequently imputed using the R package "accelmissing" (version 2.2), with a minimum missing interval of 60 minutes. A valid day was defined as having a wear time of ≥16 hours, and NHANES participants with fewer than 4 valid days were excluded.

***Exploratory mediation analyses***

Consistent with mediation criteria, Cox regression was performed to examine the associations of inflammation biomarkers (log-transformed and standardized) with CVD events. Generalized linear models (GLM) assessed the associations of CRAR parameters with inflammation biomarkers. Inflammatory biomarkers were measured at time points proximate to the CRAR parameters, whereas CVD events occurred during subsequent follow-up; therefore, this mediation analyses were performed solely as an exploratory investigation.

Prior to exploratory mediation analyses, several key assumptions were established to ensure reliability: a exploratory relationship between exposure (significant CRAR parameters), mediators (inflammation biomarkers), and outcomes (CVD incidence, all-cause and CVD mortality); linear relationships among these variables; and absence of unmeasured confounding factors influencing both mediator and outcome simultaneously. Based on these assumptions, exploratory mediation analyses were conducted using the R package "CMAverse" with 1,000 bootstrap resamples to estimate indirect effects and their 95% confidence intervals.

***Supplementary Tables***

**Table S1.** Strengthening the Reporting of Observational Studies in Epidemiology Checklist for this cohort study.

|  | **Item No** | **Recommendation** | **Section** |
| --- | --- | --- | --- |
| **Title and abstract** | 1 | (*a*) Indicate the study’s design with a commonly used term in the title or the abstract | **Title** |
|  |  | (*b*) Provide in the abstract an informative and balanced summary of what was done and what was found | **Abstract** |
| **Introduction** |  |  |  |
| Background/rationale | 2 | Explain the scientific background and rationale for the investigation being reported | **Introduction** – Paragraph 1-2 |
| Objectives | 3 | State specific objectives, including any prespecified hypotheses | **Introduction** – Paragraph 3 |
| **Methods** |  |  |  |
| Study design | 4 | Present key elements of study design early in the paper | **Methods** – Study design and population |
| Setting | 5 | Describe the setting, locations, and relevant dates, including periods of recruitment, exposure, follow-up, and data collection | **Methods** – Study design and population |
| Participants | 6 | (*a*) Give the eligibility criteria, and the sources and methods of selection of participants. Describe methods of follow-up | **Methods** – Study design and population |
|  |  | (*b*) For matched studies, give matching criteria and number of exposed and unexposed | - |
| Variables | 7 | Clearly define all outcomes, exposures, predictors, potential confounders, and effect modifiers. Give diagnostic criteria, if applicable | **Methods** – Assessment of CRAR parameters, Definitions of CKM stages, Ascertainment of incidence and mortality outcomes,  Covariates and mediators;  ***Supplementary Materials*** – *Tables S2-S6* |
| Data sources / measurement | 8* | For each variable of interest, give sources of data and details of methods of assessment (measurement). Describe comparability of assessment methods if there is more than one group | ***Supplementary Materials*** – *Supplemental Methods* |
| Bias | 9 | Describe any efforts to address potential sources of bias | **Methods** – Statistical analysis |
| Study size | 10 | Explain how the study size was arrived at | **Methods** – Study design and population |
| Quantitative variables | 11 | Explain how quantitative variables were handled in the analyses. If applicable, describe which groupings were chosen and why | **Methods** – Assessment of CRAR parameters,  Covariates and mediators |
| Statistical methods | 12 | (*a*) Describe all statistical methods, including those used to control for confounding | **Methods** – Statistical analysis– Paragraph 1-4 |
|  |  | (*b*) Describe any methods used to examine subgroups and interactions | **Methods** – Statistical analysis – Paragraph 3 |
|  |  | (*c*) Explain how missing data were addressed | **Methods** – Covariates and mediators; ***Supplementary Materials*** – *Table S7* |
|  |  | (*d*) If applicable, explain how loss to follow-up was addressed | **Methods** – Ascertainment of incidence and mortality outcomes |
|  |  | (*e*) Describe any sensitivity analyses | **Methods** – Statistical analysis – Paragraph 3 |
| **Results** |  |  |  |
| Participants | 13* | (*a*) Report numbers of individuals at each stage of study—e.g., numbers potentially eligible, examined for eligibility, confirmed eligible, included in the study, completing follow-up, and analyzed | **Results** – Baseline characteristics; ***Supplementary Materials*** – *Tables S8-S9* |
|  |  | (*b*) Give reasons for non-participation at each stage | - |
|  |  | (*c*) Consider use of a flow diagram | ***Supplementary Materials*** – *Figures S1-S2* |
| Descriptive data | 14* | (*a*) Give characteristics of study participants (e.g., demographic, clinical, social) and information on exposures and potential confounders | **Results** – Table 1; ***Supplementary Materials*** – *Tables S8-S9* |
|  |  | (*b*) Indicate number of participants with missing data for each variable of interest | ***Supplementary Materials*** – *Table S7* |
|  |  | (*c*) Summarise follow-up time (e.g., average and total amount) | **Results** – Baseline characteristics |
| Outcome data | 15* | Report numbers of outcome events or summary measures over time | **Results** – Section 2-3 |
| Main results | 16 | (*a*) Give unadjusted estimates and, if applicable, confounder-adjusted estimates and their precision (e.g., 95% confidence interval). Make clear which confounders were adjusted for and why they were included | **Results** – Section 2-6 – Tables 2-3 and Figures 1-4 |
|  |  | (*b*) If relevant, consider translating estimates of relative risk into absolute risk for a meaningful time period | - |
| Other analyses | 17 | Report other analyses done—e.g., analyses of subgroups and interactions, and sensitivity analyses | **Results** – Section 5; ***Supplementary Materials*** – *Tables S10-S20* and *Figures S3-S29* |
| **Discussion** |  |  |  |
| Key results | 18 | Summarise key results with reference to study objectives | **Discussion** – Paragraph 1 |
| Limitations | 19 | Discuss limitations of the study, taking into account sources of potential bias or imprecision. Discuss both direction and magnitude of any potential bias | **Discussion** – Paragraph 5 |
| Interpretation | 20 | Give a cautious overall interpretation of results considering objectives, limitations, multiplicity of analyses, results from similar studies, and other relevant evidence | **Discussion** – Paragraph 2-4 |
| Generalisability | 21 | Discuss the generalisability (external validity) of the study results | **Discussion** – Paragraph 5 |
| **Other information** |  |  |  |
| Funding | 22 | Give the source of funding and the role of the funders for the present study and, if applicable, for the original study on which the present article is based | **Funding** |

* Give information separately for exposed and unexposed groups.

### **Note:** An Explanation and Elaboration article provides a detailed discussion of each checklist item, along with methodological background and examples of transparent reporting. The STROBE checklist is most effectively used alongside this article, which is freely accessible on the websites of PLoS Medicine (<http://www.plosmedicine.org>), Annals of Internal Medicine (<http://www.annals.org/>), and Epidemiology (<http://www.epidem.com/>). More information on the STROBE Initiative can be found at <http://www.strobe-statement.org.>

**Table S2.** The meaning of accelerometer-measured circadian rest-activity rhythm parameters.

| **CRAR** | **Unit** | **Definition** | **Meaning** |
| --- | --- | --- | --- |
| RA | \ | RA = (M10 - L5) / (M10 + L5).  RA quantifies the relative difference between the M10 and L5 values within a 24-hour cycle, yielding a value within the range of 0 to 1. | A higher RA value indicates a greater difference in activity levels between the most and least active periods, reflecting a more pronounced circadian rhythm, possibly due to increased daytime activity, adequate sleep, or both. |
| IS | \ | IS is the CARA parameter that reflects the consistency between the daily and 24-hour circadian rhythms (day-to-day stability), with a range from 0 to 1. | A higher IS value indicates a more stable circadian rhythm, reflecting more regular routines. |
| IV | \ | IV is the CARA parameter that reflects the fragmentation of the daily circadian rhythm, with values greater than 0. | A higher IV value indicates a greater degree of circadian rhythm fragmentation, reflecting more irregular routines. |
| M10 | Milli-gravity | The average activity during the most active 10-hour period within 24 hours serves as an indicator of activity intensity during wakefulness. | A higher M10 value indicates a higher average activity level during the most active 10-hour period, reflecting greater daytime activity intensity. |
| L5 | Milli-gravity | The average activity during the least active 5-hour period within 24 hours offers insight into the activity pattern during rest. | A higher L5 value indicates a higher average activity level during the least active 5-hour period, reflecting increased activity intensity during the night (including at sleep onset). |
| M10 onset time | Hours | The start time of M10, represented as a floating-point number in the 0-24 hour format. | It indicates whether a person is more active earlier or later in the day, with a smaller value reflecting an earlier wake-up time. |
| L5 onset time | Hours | The start time of L5 (sleep onset time), expressed as a floating-point number in the 0-24 hour format. | It indicates whether inactivity corresponding to sleep occurs earlier or later in the day, with a smaller value reflecting an earlier sleep onset. |

**Abbreviations:** CRAR, circadian rest-activity rhythm; RA, relative amplitude; IS, interdaily stability; IV, intradaily variability; M10, the most active 10-h period; L5, the least active 5-h period.

**Table S3.** Definition of CKM conditions and indicators in the UKB and NHANES participants.

| **CKM conditions** | **Definition** | **CKM indicators** | **Threshold for CKM indicators** |
| --- | --- | --- | --- |
| **CVD** | Individuals with clinical CVD or subclinical CVD | Clinical CVD | **(Only in UKB)** Self-reported or clinically diagnosed CVD, satisfying at least one of the following:   1. Heart failure; 2. Coronary heart disease; 3. Atrial fibrillation; 4. Peripheral artery disease; 5. Stroke   **(Only in NHANES)** Self-reported CVD, satisfying at least one of the following:   1. Congestive heart failure; 2. Coronary heart disease; 3. Angina; 4. Heart attack; 5. Stroke |
|  |  | Subclinical CVD | Predicted 10-year CVD risk ≥ 20% based on the American Heart Association (AHA) PREVENT risk model |
| **Kidney diseases** | Individuals with CKD in KDIGO classification | Very high-risk CKD | Satisfying at least one of the following:   1. 30 ≤ eGFR < 45 mL/min/1.73m^2^ and UACR ≥ 30 mg/g; 2. 45 ≤ eGFR < 60 mL/min/1.73m^2^ and UACR ≥ 300 mg/g; 3. eGFR < 30 mL/min/1.73m^2^ |
|  |  | Moderate-to-high-risk CKD | Satisfying at least one of the following:   1. 30 ≤ eGFR < 45 mL/min/1.73m^2^ and UACR < 30 mg/g; 2. 45 ≤ eGFR < 60 mL/min/1.73m^2^ and 30 ≤ UACR < 300 mg/g; 3. eGFR ≥ 60 mL/min/1.73m^2^ and 30 ≤ UACR ≥ 300 mg/g |
|  |  | Low-risk CKD | Satisfying at least one of the following:   1. eGFR ≥ 45 mL/min/1.73m^2^ and UACR < 30 mg/g; 2. eGFR ≥ 60 mL/min/1.73m^2^ and UACR < 300 mg/g |
| **Metabolic disorders** | Individuals with overweight/obesity, abdominal obesity, prediabetes, diabetes, hypertension, hypertriglyceridemia or MetS | Overweight/obesity | BMI ≥ 25 kg/m^2^ or ≥ 23 kg/m^2^ if Asian ancestry |
|  |  | Abdominal obesity | Waist circumference ≥ 102/88 cm in male/female or ≥ 90/80 cm in male/female if Asian ancestry |
|  |  | Prediabetes | Satisfy the following **third** condition and **at least one** of the first or second conditions:   1. 5.7% ≤ HbA1c < 6.5%; 2. 100 ≤ fasting blood glucose < 126 mg/dL **(only in NHANES)**; 3. Without self-reported or clinically diagnosed diabetes, use of insulin, or oral hypoglycemic agents |
|  |  | Diabetes | Satisfying at least one of the following:   1. HbA1c ≥ 6.5%; 2. Fasting blood glucose ≥ 126 mg/dL **(only in NHANES)**; 3. Self-reported or clinically diagnosed diabetes; 4. Use of insulin, or oral hypoglycemic agents |
|  |  | Hypertension | Satisfying at least one of the following:   1. SBP ≥ 130 mmHg; 2. DBP ≥ 80 mmHg; 3. Self-reported or clinically diagnosed hypertension; 4. Use of antihypertensive medications |
|  |  | Hypertriglyceridemia | Triglycerides ≥ 135 mg/dL |
|  |  | MetS | Satisfying **at least three** of the following:   1. Abdominal obesity (Waist circumference ≥ 102/88 cm in male/female or ≥ 90/80 cm in male/female if Asian ancestry); 2. Hypertension (SBP ≥ 130 mmHg or DBP ≥ 80 mmHg or self-reported/clinically diagnosed hypertension or use of antihypertensive medications); 3. HbA1c ≥ 5.7% or fasting blood glucose ≥ 100 mg/dL **(only in NHANES)**; 4. Triglycerides ≥ 150 mg/dL; 5. HDL-C < 40/50 mg/dL in male/female |

**Abbreviations:** CKM, cardiovascular-kidney-metabolic syndrom; UKB, UK Biobank; NHANES, National Health and Nutrition Examination Survey; CVD, cardiovascular disease; KDIGO, The Kidney Disease: Improving Global Outcomes; CKD, chronic kidney disease; eGFR, estimated glomerular filtration rate; UACR, urinary albumin to creatinine ratio; MetS, metabolic syndrome; BMI, body mass index; HbA1c, glycated hemoglobin; SBP, systolic blood pressure; DBP, diastolic blood pressure; HDL-C, high-density lipoprotein cholesterol.

**Table S4.** Detailed algorithm for evaluating each CKM stage in the UKB and NHANES participants.

| **CKM stages** | **Definition** | **Criterion** | **Threshold for CKM conditions** |
| --- | --- | --- | --- |
| **Stage 0:**  No CKM risk factors | Individuals with normal BMI and waist circumference, normoglycemia, normotension, a normal lipid profile, and no evidence of CKD or subclinical or clinical CVD | All criteria are **not** met | Overweight/obesity |
|  |  |  | Abdominal obesity |
|  |  |  | Prediabetes and diabetes |
|  |  |  | Hypertension |
|  |  |  | Hypertriglyceridemia |
|  |  |  | MetS |
|  |  |  | Subclinical CVD |
|  |  |  | Clinical CVD |
|  |  | Met | Low-risk CKD |
| **Stage 1:**  Excess or dysfunctional adiposity | Individuals with overweight/obesity, abdominal obesity, or dysfunctional adipose tissue, without the presence of other metabolic risk factors or CKD | Any of the three criteria is met | Overweight/obesity |
|  |  |  | Abdominal obesity |
|  |  |  | Prediabetes |
|  |  | All criteria are **not** met | Diabetes |
|  |  |  | Hypertension |
|  |  |  | Hypertriglyceridemia |
|  |  |  | MetS |
|  |  |  | Subclinical CVD |
|  |  |  | Clinical CVD |
|  |  | Met | Low-risk CKD |
| **Stage 2:**  Metabolic risk factors and CKD | Individuals with metabolic risk factors (hypertriglyceridemia, hypertension, MetS, diabetes), or CKD | Any of the five criteria is met | Diabetes |
|  |  |  | Hypertension |
|  |  |  | Hypertriglyceridemia |
|  |  |  | MetS |
|  |  |  | Moderate-to-high-risk CKD |
|  |  | All criteria are **not** met | Very high-risk CKD |
|  |  |  | Subclinical CVD |
|  |  |  | Clinical CVD |
| **Stage 3:**  Subclinical CVD in CKM | Subclinical CVD among individuals with excess/dysfunctional adiposity, other metabolic risk factors, or CKD | Any of the two criteria is met | Very high-risk CKD |
|  |  |  | Subclinical CVD |
|  |  | Any of the eight criteria is met | Overweight/obesity |
|  |  |  | Abdominal obesity |
|  |  |  | Prediabetes |
|  |  |  | Diabetes |
|  |  |  | Hypertension |
|  |  |  | Hypertriglyceridemia |
|  |  |  | MetS |
|  |  |  | Moderate-to-high-risk CKD |
|  |  | **Not** met | Clinical CVD |
| **Stage 4:**  Clinical CVD in CKM | Clinical CVD among individuals with excess/dysfunctional adiposity, other metabolic risk factors, or CKD | Met | Clinical CVD |
|  |  | Any of the nine criteria is met | Overweight/obesity |
|  |  |  | Abdominal obesity |
|  |  |  | Prediabetes |
|  |  |  | Diabetes |
|  |  |  | Hypertension |
|  |  |  | Hypertriglyceridemia |
|  |  |  | MetS |
|  |  |  | Moderate-to-high-risk CKD |
|  |  |  | Very high-risk CKD |

**Abbreviations:** CKM, cardiovascular-kidney-metabolic syndrom; UKB, UK Biobank; NHANES, National Health and Nutrition Examination Survey; BMI, body mass index; CKD, chronic kidney disease; CVD, cardiovascular disease; MetS, metabolic syndrome.

**Table S5.** Identification of outcomes in the UKB and NHANES participants.

| **Disease** | **ICD-10 Codes** | **No. of cases** | **Female** |
| --- | --- | --- | --- |
| **Incidence outcomes (only in UKB) ^a^** | | | |
| CVD | I20, I21, I22, I23, I24, I25, I26, I48, I50, I60, I61, I63, I64, I69, I70, I71, I73, I80, G45 | 6,777 | 42.6% |
| CHD | I20, I21, I22, I23, I24, I25 | 2,672 | 37.5% |
| Atrial fibrillation | I48 | 2,298 | 40.0% |
| Heart failure | I50 | 841 | 41.3% |
| Stroke | I60, I61, I63, I64, I69 | 757 | 46.2% |
| PVD | I70, I73 | 599 | 50.1% |
| **Mortality outcomes ^b^** | | | |
| All-cause | - | **(In UKB)** 3,048 | 38.4% |
|  |  | **(In NHANES)** 438 | 44.5% |
| CVD | I00-I09, I11, I13, I20-I51, I60-I69 | **(In UKB)** 563 | 28.8% |
|  |  | **(In NHANES)** 123 | 43.1% |

**^a^** The incidence outcomes were observed in a population with CKM stages 0-3 and no CVD diagnosis.

**^b^** The mortality outcomes were observed in a population with CKM stages 1-4.

**Abbreviations:** UKB, UK Biobank; NHANES, National Health and Nutrition Examination Survey; ICD-10, International Classification of Diseases, 10th Revision; CVD, cardiovascular disease; CHD, coronary artery disease; PVD, peripheral vascular disease; CKM, cardiovascular-kidney-metabolic syndrom.

**Table S6.** Definitions and descriptions of covariates in the UKB and NHANES participants.

| **Variable** | **Unit / Categorizations** | **UKB Field ID /**  **NHANES Variable Name** | **Descriptions** |
| --- | --- | --- | --- |
| Age | Years | (In UKB) 21003, 53, 90010 | **(In UKB)** Age at accelerometer wear: Age at assessment center visit plus the difference between accelerometer wear start date and visit date. |
|  |  | (In NHANES) RIDAGEYR | **(In NHANES)** Age at screening: Due to the less than one-year difference between accelerometer wear and screening start, age at screening is used as a substitute. |
| Sex | Male; female | 31 | - |
|  |  | RIAGENDR |  |
| Race/Ethnicity | White; non-White | 21000 | White or non-White (including Mixed, Asian or Asian British, Black or Black British, and Chinese). |
|  |  | RIDRETH3 | White (non-Hispanic White) or non-White (including Mexican American, other Hispanic, non-Hispanic Black, non-Hispanic Asian, and other race). |
| Education level | College or above; high school or equivalent; less than high school | 6138 | Highest educational qualifications obtained. |
|  |  | DMDEDUC2 |  |
| TDI  **(only in UKB)** | / | 22189 | TDI is based on four socioeconomic variables (unemployment, non-car ownership, non-home ownership, and household overcrowding). A higher TDI denotes lower area-level socioeconomic status. |
| PIR  **(only in NHANES)** | Low income (<1.3); middle income (1.3-3.5); high income (>3.5) | INDFMPIR | PIR accounts for inflation and household size. A higher PIR indicates higher household income relative to the federal poverty line. |
| BMI | kg/m^2^ | 21001 | Calculated by dividing weight (kg) by the square of height (m). |
|  |  | BMXBMI |  |
| Smoking status | Current; previous; never smoking | 20116 | - |
|  |  | SMQ020, SMQ040 | Never smoked (<100 cigarettes lifetime), former smoker (≥100 cigarettes lifetime but not currently), or current smoker (≥100 cigarettes lifetime and currently smoking). |
| Drinking status | Yes; no | 20117 | Yes (currently drinking) or no (never drank, or former drinker). |
|  |  | ALQ120Q | Yes (drank ≥12 times in the past year) or no (drank <12 times in the past year). |
| Healthy diet score  **(only in UKB)** | /  (Range: 0-5) | 1309, 1319, 1289, 1299, 1329, 1339, 1349, 1369, 1379, 1389 | UKB Food Frequency Questionnaire, 1 point for meeting each criterion, cumulative score:  1. Fruits: ≥3 pieces/day  2. Vegetables: ≥4 tablespoons/day  3. Fish: ≥2 times/week  4. Processed meats: ≤2 times/week  5. Unprocessed red meats: ≤2 times/week |
| Shift work  **(only in UKB)** | Yes; no | 826 | Yes (sometimes/usually/always shift work) or no (never/rarely shift work). |
| Hypertension | Yes; no | 4080, 93, 4079, 94, 41270, 41280, 20002, 20008, 6153, 6177, 20003 | Satisfying at least one of the following indicates hypertension:   1. SBP ≥130 mmHg; 2. DBP ≥80 mmHg; 3. Self-reported or clinically diagnosed hypertension; 4. Use of antihypertensive medications |
|  |  | BPXSY1, BPXSY2, BPXSY3, BPXSY4, BPXDI1, BPXDI2, BPXDI3, BPXDI4, BPQ020, BPQ050A, RXDDRGID |  |
| Diabetes | Yes; no | 30750, 41270, 41280, 20002, 20008, 6153, 6177, 20003 | Satisfying at least one of the following indicates diabetes:   1. HbA1c ≥6.5%; 2. Fasting blood glucose ≥126 mg/dL **(only in NHANES)**; 3. Self-reported or clinically diagnosed diabetes; 4. Use of insulin, or oral hypoglycemic agents |
|  |  | LBXGH, LBXGLU, DIQ010, DIQ050, DIQ070, RXDDRGID |  |
| Hyperlipidemia | Yes; no | 30870, 30690, 30760, 30780, 6153, 6177, 20003 | Satisfying at least one of the following indicates hyperlipidemia:   1. Triglycerides ≥150 mg/dL; 2. Total cholesterol ≥200 mg/dL; 3. HDL-C <40/50 mg/dL in male/female; 4. LDL-C ≥160 mg/dL; 5. Use of antihyperlipidemic medications |
|  |  | LBXSTR, LBXTC, LBDHDD, LBDLDL, RXDDRGID |  |
| Depression | Yes; no | 130894, 130895, 130896, 130897 | Diagnosed depression is considered if the first reported depressive episode or recurrent depressive disorder precedes the accelerometer wear start. |
|  |  | DPQ010, DPQ020, DPQ030, DPQ040, DPQ050, DPQ060, DPQ070, DPQ080, DPQ090 | Based on the 9 questions of the PHQ-9, a total score of ≥10 indicates depression. |
| Cancer | Yes; no | 84 | Diagnosed cancer is considered if the first occurred cancer precedes the accelerometer wear start. |
|  |  | MCQ220 | Self-reported doctor-diagnosed cancer. |
| MVPA | Minutes/week | 90004 | Weekly duration of moderate to vigorous activity (average ENMO >100 milli-gravity) measured by accelerometer. |
|  |  | PAXMTSM |  |
| Sleep duration  **(only in UKB)** | Hours/day | Return ID  1862 | Total sleep time measured by accelerometer. |
| Sleep efficiency  **(only in UKB)** | %  (Range: 0-1) | Return ID  1862 | Sleep efficiency is the ratio of total sleep time to time in bed measured by accelerometer. |
| Season of accelerometer wear  **(only in UKB)** | Spring; summer; autumn; winter | 90010 | The start time of accelerometer wear is in spring (March to May), summer (June to August), autumn (September to November), and winter (December to February). |

**Abbreviations:** UKB, UK Biobank; NHANES, National Health and Nutrition Examination Survey; TDI, Townsend deprivation index; PIR, poverty income ratio; BMI, body mass index; SBP, systolic blood pressure; DBP, diastolic blood pressure; HbA1c, glycated hemoglobin; HDL-C, high-density lipoprotein cholesterol; LDL-C, low-density lipoprotein cholesterol; PHQ-9, Patient Health Questionnaire-9; MVPA, moderate to vigorous physical activity; ENMO, Euclidean norm minus one.

**Table S7.** Proportions of missing value in the UKB and NHANES participants.

| **Variable** | **N** | **Percentage, %** |
| --- | --- | --- |
| **Overall participants in UKB (n = 74,777)** | | |
| HbA1c | 3,482 | 4.66 |
| Diabetes | 3,437 | 4.60 |
| Healthy diet score | 423 | 0.57 |
| Education level | 245 | 0.33 |
| Sleep efficiency | 184 | 0.25 |
| Sleep duration | 184 | 0.25 |
| TDI | 79 | 0.11 |
| Smoking status | 39 | 0.05 |
| BMI | 33 | 0.04 |
| Drinking status | 24 | 0.03 |
| Hyperlipidemia | 19 | 0.03 |
| SBP | 17 | 0.02 |
| Total cholesterol | 10 | 0.01 |
| **Overall participants in NHANES (n = 6,046)** | | |
| PIR | 448 | 7.41 |
| Depression | 439 | 7.26 |
| Drinking status | 404 | 6.68 |
| BMI | 40 | 0.66 |
| Smoking status | 4 | 0.07 |
| Education level | 3 | 0.05 |
| Cancer | 1 | 0.02 |

**Abbreviations:** UKB, UK Biobank; NHANES, National Health and Nutrition Examination Survey; HbA1c, glycated hemoglobin; TDI, Townsend deprivation index; BMI, body mass index; SBP, systolic blood pressure; PIR, poverty income ratio.

**Table S8.** Baseline characteristics of participants from the UKB categorized by CKM stages **^a^**.

| **Characteristics** | **Overall** | **CKM stages** | | | | | ***P*** **^b^** |
| --- | --- | --- | --- | --- | --- | --- | --- |
|  |  | **Stage 0** | **Stage 1** | **Stage 2** | **Stage 3** | **Stage 4** |  |
| **No. of participants** | 74,777 | 8,944 (12.0) | 4,681 (6.3) | 55,128 (73.7) | 1,891 (2.5) | 4,133 (5.5) |  |
| **Age (years)** | 62.0 (55.0, 68.0) | 57.0 (51.0, 64.0) | 57.0 (51.0, 64.0) | 63.0 (56.0, 67.0) | 72.0 (69.0, 74.0) | 67.0 (63.0, 71.0) | <0.001 |
| **Sex** |  |  |  |  |  |  | <0.001 |
| Male | 33,524 (44.8) | 1,933 (21.6) | 1,258 (26.9) | 25,893 (47.0) | 1,602 (84.7) | 2,838 (68.7) |  |
| Female | 41,253 (55.2) | 7,011 (78.4) | 3,423 (73.1) | 29,235 (53.0) | 289 (15.3) | 1,295 (31.3) |  |
| **Race/Ethnicity** |  |  |  |  |  |  | <0.001 |
| White | 72,563 (97.0) | 8,739 (97.7) | 4,462 (95.3) | 53,477 (97.0) | 1,858 (98.3) | 4,027 (97.4) |  |
| Non-White | 2,214 (3.0) | 205 (2.3) | 219 (4.7) | 1,651 (3.0) | 33 (1.7) | 106 (2.6) |  |
| **Education level** |  |  |  |  |  |  | <0.001 |
| College or above | 17,248 (23.1) | 1,489 (16.6) | 986 (21.1) | 12,909 (23.4) | 598 (31.6) | 1,266 (30.6) |  |
| High school or equivalent | 4,637 (6.2) | 608 (6.8) | 266 (5.7) | 3,445 (6.2) | 99 (5.2) | 219 (5.3) |  |
| Less than high school | 52,892 (70.7) | 6,847 (76.6) | 3,429 (73.3) | 38,774 (70.3) | 1,194 (63.1) | 2,648 (64.1) |  |
| **TDI** | -2.5 (-3.8, -0.2) | -2.4 (-3.8, 0.0) | -2.3 (-3.7, 0.1) | -2.5 (-3.8, -0.3) | -2.6 (-3.9, -0.5) | -2.3 (-3.8, 0.0) | <0.001 |
| **BMI (kg/m^2^)** | 26.0 (23.6, 29.0) | 22.5 (21.2, 23.7) | 26.8 (25.7, 28.5) | 26.5 (24.1, 29.4) | 28.2 (25.9, 31.0) | 27.7 (25.2, 30.8) | <0.001 |
| **Smoking status** |  |  |  |  |  |  | <0.001 |
| Never smoking | 42,697 (57.1) | 5,782 (64.6) | 2,790 (59.6) | 31,686 (57.5) | 672 (35.5) | 1,767 (42.8) |  |
| Previous smoking | 26,921 (36.0) | 2,582 (28.9) | 1,579 (33.7) | 19,856 (36.0) | 851 (45.0) | 2,053 (49.7) |  |
| Current smoking | 5,159 (6.9) | 580 (6.5) | 312 (6.7) | 3,586 (6.5) | 368 (19.5) | 313 (7.6) |  |
| **Drinking status** |  |  |  |  |  |  | <0.001 |
| No | 4,065 (5.4) | 468 (5.2) | 233 (5.0) | 2,927 (5.3) | 115 (6.1) | 322 (7.8) |  |
| Yes | 70,712 (94.6) | 8,476 (94.8) | 4,448 (95.0) | 52,201 (94.7) | 1,776 (93.9) | 3,811 (92.2) |  |
| **Healthy diet score** | 3.0 (2.0, 4.0) | 3.0 (2.0, 4.0) | 3.0 (2.0, 4.0) | 3.0 (2.0, 4.0) | 3.0 (2.0, 3.0) | 3.0 (2.0, 4.0) | <0.001 |
| **Shift work** |  |  |  |  |  |  | <0.001 |
| No | 68,727 (91.9) | 8,248 (92.2) | 4,220 (90.2) | 50,511 (91.6) | 1,833 (96.9) | 3,915 (94.7) |  |
| Yes | 6,050 (8.1) | 696 (7.8) | 461 (9.8) | 4,617 (8.4) | 58 (3.1) | 218 (5.3) |  |
| **Hypertension** |  |  |  |  |  |  | <0.001 |
| No | 19,845 (26.5) | 8,944 (100.0) | 4,681 (100.0) | 5,983 (10.9) | 17 (0.9) | 221 (5.3) |  |
| Yes | 54,932 (73.5) | 0 (0.0) | 0 (0.0) | 49,145 (89.1) | 1,874 (99.1) | 3,912 (94.7) |  |
| **Diabetes** |  |  |  |  |  |  | <0.001 |
| No | 72,303 (96.7) | 8,944 (100.0) | 4,681 (100.0) | 53,699 (97.4) | 1,295 (68.5) | 3,684 (89.1) |  |
| Yes | 2,474 (3.3) | 0 (0.0) | 0 (0.0) | 1,429 (2.6) | 596 (31.5) | 449 (10.9) |  |
| **Hyperlipidemia** |  |  |  |  |  |  | <0.001 |
| No | 10,586 (14.2) | 3,017 (33.7) | 1,180 (25.2) | 5,990 (10.9) | 157 (8.3) | 242 (5.9) |  |
| Yes | 64,191 (85.8) | 5,927 (66.3) | 3,501 (74.8) | 49,138 (89.1) | 1,734 (91.7) | 3,891 (94.1) |  |
| **Depression** |  |  |  |  |  |  | <0.001 |
| No | 68,439 (91.5) | 8,186 (91.5) | 4,241 (90.6) | 50,515 (91.6) | 1,764 (93.3) | 3,733 (90.3) |  |
| Yes | 6,338 (8.5) | 758 (8.5) | 440 (9.4) | 4,613 (8.4) | 127 (6.7) | 400 (9.7) |  |
| **Cancer** |  |  |  |  |  |  | <0.001 |
| No | 67,803 (90.7) | 8,233 (92.1) | 4,328 (92.5) | 49,929 (90.6) | 1,611 (85.2) | 3,702 (89.6) |  |
| Yes | 6,974 (9.3) | 711 (7.9) | 353 (7.5) | 5,199 (9.4) | 280 (14.8) | 431 (10.4) |  |
| **eGFR (mL/min/1.73m^2^)** | 92.5 (83.1, 101.1) | 98.9 (90.6, 106.4) | 95.4 (86.5, 103.7) | 92.0 (83.0, 100.5) | 79.4 (67.8, 88.1) | 85.3 (75.4, 94.2) | <0.001 |
| **MVPA (minutes/week)** | 115.3 (53.7, 213.0) | 162.2 (88.4, 276.5) | 122.8 (60.8, 217.0) | 113.5 (53.3, 209.3) | 57.3 (22.2, 126.2) | 67.8 (27.2, 144.5) | <0.001 |
| **Sleep duration (hours/day)** | 7.3 (6.8, 7.9) | 7.4 (6.8, 7.9) | 7.3 (6.7, 7.8) | 7.3 (6.7, 7.9) | 7.3 (6.7, 7.9) | 7.3 (6.7, 7.9) | <0.001 |
| **Sleep efficiency (%)** | 76.7 (71.9, 81.0) | 78.1 (73.6, 82.2) | 77.1 (72.4, 81.2) | 76.5 (71.8, 80.8) | 74.8 (69.8, 79.4) | 75.5 (70.4, 80.0) | <0.001 |
| **Season of accelerometer wear** |  |  |  |  |  |  | 0.048 |
| Spring | 16,849 (22.5) | 2,085 (23.3) | 1,069 (22.8) | 12,422 (22.5) | 389 (20.6) | 884 (21.4) |  |
| Summer | 19,555 (26.2) | 2,295 (25.7) | 1,214 (25.9) | 14,432 (26.2) | 491 (26.0) | 1,123 (27.2) |  |
| Autumn | 22,344 (29.9) | 2,729 (30.5) | 1,362 (29.1) | 16,436 (29.8) | 602 (31.8) | 1,215 (29.4) |  |
| Winter | 16,029 (21.4) | 1,835 (20.5) | 1,036 (22.1) | 11,838 (21.5) | 409 (21.6) | 911 (22.0) |  |
| **CRAR parameters:** |  |  |  |  |  |  |  |
| **RA ^c^** | 0.88 (0.85, 0.90) | 0.89 (0.87, 0.91) | 0.88 (0.85, 0.90) | 0.88 (0.85, 0.90) | 0.85 (0.81, 0.88) | 0.86 (0.82, 0.89) | <0.001 |
| **IS ^c^** | 0.55 (0.46, 0.63) | 0.54 (0.44, 0.62) | 0.53 (0.45, 0.62) | 0.55 (0.46, 0.63) | 0.57 (0.49, 0.64) | 0.57 (0.48, 0.64) | <0.001 |
| **IV ^c^** | 0.89 (0.74, 1.05) | 0.91 (0.76, 1.08) | 0.89 (0.75, 1.06) | 0.88 (0.74, 1.05) | 0.91 (0.76, 1.06) | 0.88 (0.74, 1.05) | <0.001 |
| **M10 (milli-gravity)** | 45.5 (37.3, 55.2) | 50.3 (41.9, 60.4) | 46.4 (38.5, 55.5) | 45.3 (37.3, 54.9) | 37.7 (30.2, 45.5) | 40.0 (32.1, 48.6) | <0.001 |
| **L5 (milli-gravity)** | 2.9 (2.5, 3.4) | 2.8 (2.5, 3.3) | 2.9 (2.5, 3.4) | 2.9 (2.5, 3.4) | 3.0 (2.6, 3.5) | 3.0 (2.6, 3.5) | <0.001 |
| **M10 onset time (hours)** | 8.4 (7.7, 9.4) | 8.5 (7.7, 9.6) | 8.6 (7.7, 9.6) | 8.4 (7.7, 9.3) | 8.2 (7.6, 9.0) | 8.4 (7.6, 9.2) | <0.001 |
| **L5 onset time (hours)** | 24.9 (24.1, 25.6) | 24.8 (24.1, 25.5) | 24.9 (24.1, 25.6) | 24.9 (24.1, 25.6) | 24.9 (24.1, 25.8) | 24.9 (24.1, 25.8) | <0.001 |

**^a^** Given the non-normal distribution of continuous variables, these are presented as median (interquartile range), while categorical variables are expressed as n (%). All values are rounded to one decimal place for precision.

**^b^** Due to the non-normal distribution, the Kruskal-Wallis rank sum test was used for continuous variables, while the Pearson's Chi-squared test was applied to categorical variables.

**^c^** Since the continuous variable has a narrow range (1-2), it was rounded to two decimal places for clarity in the description.

**Abbreviations:** UKB, UK Biobank; CKM, cardiovascular-kidney-metabolic syndrom; TDI, Townsend deprivation index; BMI, body mass index; eGFR, estimated glomerular filtration rate; MVPA, moderate to vigorous physical activity; CRAR, circadian rest-activity rhythm; RA, relative amplitude; IS, interdaily stability; IV, intradaily variability; M10, the most active 10-h period; L5, the least active 5-h period.

**Table S9.** Baseline characteristics of participants from the NHANES categorized by CKM stages **^a^**.

| **Characteristics** | **Overall** | **CKM stages** | | | | | ***P*** **^b^** |
| --- | --- | --- | --- | --- | --- | --- | --- |
|  |  | **Stage 0** | **Stage 1** | **Stage 2** | **Stage 3** | **Stage 4** |  |
| **No. of participants** | 6,046 | 369 (6.1) | 958 (15.8) | 3723 (61.6) | 345 (5.7) | 651 (10.8) |  |
| **Age (years)** | 53.0 (42.0, 64.0) | 41.0 (35.0, 50.0) | 43.0 (36.0, 53.0) | 52.0 (43.0, 62.0) | 73.0 (68.0, 76.0) | 65.0 (57.0, 72.0) | <0.001 |
| **Sex** |  |  |  |  |  |  | <0.001 |
| Male | 2,930 (48.5) | 124 (33.6) | 404 (42.2) | 1,841 (49.4) | 212 (61.4) | 349 (53.6) |  |
| Female | 3,116 (51.5) | 245 (66.4) | 554 (57.8) | 1,882 (50.6) | 133 (38.6) | 302 (46.4) |  |
| **Race/Ethnicity** |  |  |  |  |  |  | <0.001 |
| White | 2,432 (40.2) | 185 (50.1) | 365 (38.1) | 1,436 (38.6) | 143 (41.4) | 303 (46.5) |  |
| Non-White | 3,614 (59.8) | 184 (49.9) | 593 (61.9) | 2,287 (61.4) | 202 (58.6) | 348 (53.5) |  |
| **Education level** |  |  |  |  |  |  | <0.001 |
| College or above | 1,351 (22.3) | 37 (10.0) | 181 (18.9) | 820 (22.0) | 116 (33.6) | 197 (30.3) |  |
| High school or equivalent | 1,360 (22.5) | 74 (20.1) | 168 (17.5) | 860 (23.1) | 83 (24.1) | 175 (26.9) |  |
| Less than high school | 3,335 (55.2) | 258 (69.9) | 609 (63.6) | 2,043 (54.9) | 146 (42.3) | 279 (42.9) |  |
| **PIR** |  |  |  |  |  |  | <0.001 |
| Low income (<1.3) | 2,016 (33.3) | 95 (25.7) | 273 (28.5) | 1,219 (32.7) | 128 (37.1) | 301 (46.2) |  |
| Middle income (1.3-3.5) | 2,069 (34.2) | 94 (25.5) | 329 (34.3) | 1,275 (34.2) | 144 (41.7) | 227 (34.9) |  |
| High income (>3.5) | 1,961 (32.4) | 180 (48.8) | 356 (37.2) | 1,229 (33.0) | 73 (21.2) | 123 (18.9) |  |
| **BMI (kg/m^2^)** | 28.4 (24.8, 33.1) | 22.1 (20.5, 23.3) | 27.3 (25.0, 30.5) | 29.4 (25.7, 34.1) | 28.7 (25.4, 32.6) | 30.0 (26.0, 34.9) | <0.001 |
| **Smoking status** |  |  |  |  |  |  | <0.001 |
| Never smoking | 3,293 (54.5) | 242 (65.6) | 604 (63.0) | 2,060 (55.3) | 143 (41.4) | 244 (37.5) |  |
| Previous smoking | 1,544 (25.5) | 56 (15.2) | 190 (19.8) | 925 (24.8) | 137 (39.7) | 236 (36.3) |  |
| Current smoking | 1,209 (20.0) | 71 (19.2) | 164 (17.1) | 738 (19.8) | 65 (18.8) | 171 (26.3) |  |
| **Drinking status** |  |  |  |  |  |  | 0.188 |
| No | 5,876 (97.2) | 359 (97.3) | 926 (96.7) | 3,615 (97.1) | 334 (96.8) | 642 (98.6) |  |
| Yes | 170 (2.8) | 10 (2.7) | 32 (3.3) | 108 (2.9) | 11 (3.2) | 9 (1.4) |  |
| **Hypertension** |  |  |  |  |  |  | <0.001 |
| No | 2,469 (40.8) | 369 (100.0) | 958 (100.0) | 1,025 (27.5) | 15 (4.3) | 102 (15.7) |  |
| Yes | 3,577 (59.2) | 0 (0.0) | 0 (0.0) | 2,698 (72.5) | 330 (95.7) | 549 (84.3) |  |
| **Diabetes** |  |  |  |  |  |  | <0.001 |
| No | 4,794 (79.3) | 369 (100.0) | 958 (100.0) | 2,952 (79.3) | 151 (43.8) | 364 (55.9) |  |
| Yes | 1,252 (20.7) | 0 (0.0) | 0 (0.0) | 771 (20.7) | 194 (56.2) | 287 (44.1) |  |
| **Hyperlipidemia** |  |  |  |  |  |  | <0.001 |
| No | 1,583 (26.2) | 226 (61.2) | 462 (48.2) | 664 (17.8) | 84 (24.3) | 147 (22.6) |  |
| Yes | 4,463 (73.8) | 143 (38.8) | 496 (51.8) | 3,059 (82.2) | 261 (75.7) | 504 (77.4) |  |
| **Depression** |  |  |  |  |  |  | <0.001 |
| No | 5,437 (89.9) | 348 (94.3) | 891 (93.0) | 3,356 (90.1) | 319 (92.5) | 523 (80.3) |  |
| Yes | 609 (10.1) | 21 (5.7) | 67 (7.0) | 367 (9.9) | 26 (7.5) | 128 (19.7) |  |
| **Cancer** |  |  |  |  |  |  | <0.001 |
| No | 5,469 (90.5) | 337 (91.3) | 915 (95.5) | 3,419 (91.8) | 272 (78.8) | 526 (80.8) |  |
| Yes | 577 (9.5) | 32 (8.7) | 43 (4.5) | 304 (8.2) | 73 (21.2) | 125 (19.2) |  |
| **eGFR (mL/min/1.73m^2^)** | 92.6 (77.7, 105.5) | 101.8 (89.5, 112.6) | 99.0 (87.1, 111.6) | 94.0 (80.6, 105.7) | 65.2 (48.7, 81.2) | 76.3 (59.9, 91.9) | <0.001 |
| **MVPA (minutes/week)** | 0.0 (0.0, 0.0) | 0.0 (0.0, 1.0) | 0.0 (0.0, 1.0) | 0.0 (0.0, 0.0) | 0.0 (0.0, 0.0) | 0.0 (0.0, 0.0) | <0.001 |
| **CRAR parameters:** |  |  |  |  |  |  |  |
| **RA ^c^** | 0.88 (0.80, 0.93) | 0.92 (0.86, 0.94) | 0.90 (0.83, 0.94) | 0.88 (0.80, 0.92) | 0.86 (0.77, 0.90) | 0.85 (0.75, 0.91) | <0.001 |
| **IS ^c^** | 0.60 (0.50, 0.68) | 0.60 (0.49, 0.67) | 0.60 (0.51, 0.67) | 0.60 (0.50, 0.68) | 0.61 (0.51, 0.69) | 0.58 (0.47, 0.67) | 0.003 |
| **IV ^c^** | 0.69 (0.55, 0.83) | 0.67 (0.55, 0.82) | 0.67 (0.53, 0.80) | 0.68 (0.55, 0.82) | 0.77 (0.63, 0.93) | 0.75 (0.60, 0.93) | <0.001 |
| **M10 (milli-gravity)** | 13.7 (11.3, 16.5) | 15.2 (13.1, 17.4) | 14.9 (12.5, 17.7) | 13.9 (11.5, 16.5) | 10.9 (9.1, 13.2) | 11.3 (8.7, 13.9) | <0.001 |
| **L5 (milli-gravity)** | 0.8 (0.5, 1.4) | 0.7 (0.5, 1.1) | 0.8 (0.5, 1.3) | 0.9 (0.6, 1.4) | 0.8 (0.6, 1.4) | 0.9 (0.6, 1.5) | <0.001 |
| **M10 onset time (hours)** | 8.8 (7.4, 10.5) | 9.0 (7.5, 11.0) | 9.1 (7.7, 11.0) | 8.8 (7.4, 10.6) | 8.0 (7.0, 9.1) | 8.6 (7.3, 10.0) | <0.001 |
| **L5 onset time (hours)** | 1.8 (0.9, 22.9) | 1.5 (0.8, 4.5) | 1.6 (0.8, 22.7) | 1.9 (0.9, 22.9) | 2.3 (0.9, 23.1) | 2.1 (1.0, 22.9) | <0.001 |

**^a^** Given the non-normal distribution of continuous variables, these are presented as median (interquartile range), while categorical variables are expressed as n (%). All values are rounded to one decimal place for precision.

**^b^** Due to the non-normal distribution, the Kruskal-Wallis rank sum test was used for continuous variables, while the Pearson's Chi-squared test was applied to categorical variables.

**^c^** Since the continuous variable has a narrow range (1-2), it was rounded to two decimal places for clarity in the description.

**Abbreviations:** NHANES, National Health and Nutrition Examination Survey; CKM, cardiovascular-kidney-metabolic syndrom; PIR, poverty income ratio; BMI, body mass index; eGFR, estimated glomerular filtration rate; MVPA, moderate to vigorous physical activity; CRAR, circadian rest-activity rhythm; RA, relative amplitude; IS, interdaily stability; IV, intradaily variability; M10, the most active 10-h period; L5, the least active 5-h period.

**Table S10.** Association between major circadian rest-activity rhythm and the risk of CVD incidence in individuals with CKM stages 0-3 stratified by optimal risk cut-off points.

| **CRAR** | **Events/PYs (‰)** | **CVD incidence** | |
| --- | --- | --- | --- |
|  |  | **HR (95% CI)** | ***P*** |
| **RA** |  |  |  |
| <0.87 | 3,380/197,902 (17.08) | *Reference* | - |
| ≥0.87 | 3,397/289,992 (11.36) | 0.88 (0.83, 0.93) | < 0.001 |
| **IS** |  |  |  |
| <0.47 | 1,591/138,721 (11.47) | *Reference* | - |
| ≥0.47 | 6,379/358,173 (14.48) | 1.00 (0.94, 1.06) | 0.989 |
| **IV** |  |  |  |
| <1.10 | 5,586/397,442 (14.06) | *Reference* | - |
| ≥1.10 | 1,191/99,471 (11.97) | 0.95 (0.89, 1.01) | 0.132 |

Models were adjusted for age, sex, race, education level, TDI, BMI, smoking status, drinking status, healthy diet score, shift work, hypertension, diabetes, hyperlipidemia, MVPA, sleep efficiency, sleep duration, and season of accelerometer wear.

**Abbreviations:** CVD, cardiovascular disease; CKM, cardiovascular-kidney-metabolic syndrom; CRAR, circadian rest-activity rhythm; PYs, person-years; HR, hazard ratio; CI, confidence interval; RA, relative amplitude; IS, interdaily stability; IV, intradaily variability; BMI, body mass index; TDI, Townsend deprivation index; MVPA, moderate to vigorous physical activity.

**Table S11.** Association between major circadian rest-activity rhythm and the risk of all-cause mortality in patients with CKM stages 1-4 stratified by optimal risk cut-off points.

| **CRAR** | **Events/PYs (‰)** | **All-cause mortality** | |
| --- | --- | --- | --- |
|  |  | **HR (95% CI)** | ***P*** |
| **RA** |  |  |  |
| <0.81 | 857/78,042 (10.98) | *Reference* | - |
| ≥0.81 | 2,191/536,246 (4.09) | 0.56 (0.51, 0.62) | < 0.001 |
| **IS** |  |  |  |
| <0.45 | 535/135,184 (3.96) | *Reference* | - |
| ≥0.45 | 2,513/479,104 (5.25) | 0.96 (0.87, 1.06) | 0.433 |
| **IV** |  |  |  |
| <0.68 | 394/99,230 (3.97) | *Reference* | - |
| ≥0.68 | 2,654/515,058 (5.15) | 1.26 (1.13, 1.40) | < 0.001 |

Models were adjusted for age, sex, race, education level, TDI, BMI, smoking status, drinking status, healthy diet score, shift work, hypertension, diabetes, hyperlipidemia, MVPA, sleep efficiency, sleep duration, and season of accelerometer wear.

**Abbreviations:** CKM, cardiovascular-kidney-metabolic syndrom; CRAR, circadian rest-activity rhythm; PYs, person-years; HR, hazard ratio; CI, confidence interval; RA, relative amplitude; IS, interdaily stability; IV, intradaily variability; BMI, body mass index; TDI, Townsend deprivation index; MVPA, moderate to vigorous physical activity.

**Table S12.** Association between major circadian rest-activity rhythm and the risk of CVD mortality in patients with CKM stages 1-4 stratified by optimal risk cut-off points.

| **CRAR** | **Events/PYs (‰)** | **CVD mortality** | |
| --- | --- | --- | --- |
|  |  | **HR (95% CI)** | ***P*** |
| **RA** |  |  |  |
| <0.81 | 199/78,042 (2.55) | *Reference* | - |
| ≥0.81 | 364/536,246 (0.68) | 0.49 (0.40, 0.60) | < 0.001 |
| **IS** |  |  |  |
| <0.58 | 311/363,091 (0.86) | *Reference* | - |
| ≥0.58 | 252/251,197 (1.00) | 1.03 (0.86, 1.22) | 0.768 |
| **IV** |  |  |  |
| <0.76 | 123/173,457 (0.75) | *Reference* | - |
| ≥0.76 | 440/440,831 (1.00) | 1.33 (1.08, 1.62) | 0.007 |

Models were adjusted for age, sex, race, education level, TDI, BMI, smoking status, drinking status, healthy diet score, shift work, hypertension, diabetes, hyperlipidemia, MVPA, sleep efficiency, sleep duration, and season of accelerometer wear.

**Abbreviations:** CVD, cardiovascular disease; CKM, cardiovascular-kidney-metabolic syndrom; CRAR, circadian rest-activity rhythm; PYs, person-years; HR, hazard ratio; CI, confidence interval; RA, relative amplitude; IS, interdaily stability; IV, intradaily variability; BMI, body mass index; TDI, Townsend deprivation index; MVPA, moderate to vigorous physical activity.

**Table S13.** Association between the major circadian rest-activity rhythm and the competing risk of CVD mortality in patients with CKM stages 1-4, based on the Fine-Gray model.

| **CRAR** | **Events/PYs (‰)** | **CVD mortality** | |
| --- | --- | --- | --- |
|  |  | **HR (95% CI)** | ***P*** |
| **RA** |  |  |  |
| Continuous **^a^** | 563/614,288 (0.92) | 0.69 (0.63, 0.76) | < 0.001 |
| Categories |  |  |  |
| Tertile 1 | 315/210,171 (1.50) | *Reference* | - |
| Tertile 2 | 166/206,520 (0.80) | 0.71 (0.58, 0.87) | 0.001 |
| Tertile 3 | 82/197,596 (0.41) | 0.46 (0.35, 0.60) | < 0.001 |
| **IS** |  |  |  |
| Continuous **^a^** | 563/614,288 (0.92) | 0.95 (0.88, 1.02) | 0.136 |
| Categories |  |  |  |
| Tertile 1 | 189/202,066 (0.94) | *Reference* | - |
| Tertile 2 | 184/205,293 (0.90) | 0.94 (0.77, 1.15) | 0.550 |
| Tertile 3 | 190/206,929 (0.92) | 0.94 (0.77, 1.16) | 0.584 |
| **IV** |  |  |  |
| Continuous **^a^** | 563/614,288 (0.92) | 1.04 (1.00, 1.08) | 0.037 |
| Categories |  |  |  |
| Tertile 1 | 165/207,196 (0.80) | *Reference* | - |
| Tertile 2 | 191/205,287 (0.93) | 1.09 (0.88, 1.34) | 0.417 |
| Tertile 3 | 207/201,805 (1.03) | 1.21 (0.98, 1.49) | 0.072 |

Model was adjusted for age, sex, race, education level, TDI, BMI, smoking status, drinking status, healthy diet score, shift work, hypertension, diabetes, hyperlipidemia, MVPA, sleep efficiency, sleep duration, and season of accelerometer wear.

**^a^** Since the continuous variable has a narrow range (1-2), it was scaled by a factor of ten for the analysis. The HR reflects the hazard ratio for a 0.1 unit change in the variable.

**Abbreviations:** CVD, cardiovascular disease; CKM, cardiovascular-kidney-metabolic syndrom; CRAR, circadian rest-activity rhythm; PYs, person-years; HR, hazard ratio; CI, confidence interval; RA, relative amplitude; IS, interdaily stability; IV, intradaily variability; BMI, body mass index; TDI, Townsend deprivation index; MVPA, moderate to vigorous physical activity.

**Table S14.** Associations between minor circadian rest-activity rhythm and the risk of CVD incidence in individuals with CKM stages 0-3.

| **CRAR** | **Events/PYs (‰)** | **CVD incidence** | | |
| --- | --- | --- | --- | --- |
|  |  | **HR (95% CI)** | ***P*** | ***P* for trend** **^a^** |
| **M10** |  |  |  |  |
| Continuous | 6,777/496,893 (13.64) | 1.00 (0.99, 1.00) | 0.118 |  |
| Categories |  |  |  | 0.001 |
| Tertile 1 | 2,510/157,242 (15.96) | *Reference* | - |  |
| Tertile 2 | 2,272/166,403 (13.65) | 0.94 (0.89, 1.00) | 0.045 |  |
| Tertile 3 | 1,995/173,248 (11.52) | 0.88 (0.82, 0.95) | 0.001 |  |
| **L5** |  |  |  |  |
| Continuous | 6,777/496,893 (13.64) | 1.03 (1.01, 1.04) | 0.005 |  |
| Categories |  |  |  | < 0.001 |
| Tertile 1 | 2,111/168,348 (12.54) | *Reference* | - |  |
| Tertile 2 | 2,217/166,058 (13.35) | 1.05 (0.99, 1.12) | 0.098 |  |
| Tertile 3 | 2,449/162,487 (15.07) | 1.14 (1.07, 1.21) | < 0.001 |  |
| **M10 onset time** |  |  |  |  |
| Categories |  |  |  | - |
| Tertile 1 | 2,226/165,500 (13.45) | 1.00 (0.94, 1.06) | 0.947 |  |
| Tertile 2 | 2,210/165,685 (13.34) | *Reference* | - |  |
| Tertile 3 | 2,341/165,708 (14.13) | 1.06 (1.00, 1.12) | 0.069 |  |
| **L5 onset time** |  |  |  |  |
| Categories |  |  |  | - |
| Tertile 1 | 2,174/167,455 (12.98) | 1.05 (0.99, 1.11) | 0.106 |  |
| Tertile 2 | 2,231/164,811 (13.54) | *Reference* | - |  |
| Tertile 3 | 2,372/164,628 (14.41) | 1.08 (1.02, 1.14) | 0.013 |  |

**^a^** *P* for trend was calculated using multivariable Cox regression models to evaluate the differences in the medians of continuous variables across different tertile groups.

Model was adjusted for age, sex, race, education level, TDI, BMI, smoking status, drinking status, healthy diet score, shift work, hypertension, diabetes, hyperlipidemia, MVPA, sleep efficiency, sleep duration, and season of accelerometer wear.

**Abbreviations:** CVD, cardiovascular disease; CKM, cardiovascular-kidney-metabolic syndrom; CRAR, circadian rest-activity rhythm; PYs, person-years; HR, hazard ratio; CI, confidence interval; M10, the most active 10-h period; L5, the least active 5-h period; BMI, body mass index; TDI, Townsend deprivation index; MVPA, moderate to vigorous physical activity.

**Table S15.** Associations between minor circadian rest-activity rhythm and the risk of all-cause and CVD mortality in patients with CKM stages 1-4.

| **CRAR** |  | **All-cause mortality** | | | |  | **CVD mortality** | | | |
| --- | --- | --- | --- | --- | --- | --- | --- | --- | --- | --- |
|  |  | **Events/PYs (‰)** | **HR (95% CI)** | ***P*** | ***P* for trend** **^a^** |  | **Events/PYs (‰)** | **HR (95% CI)** | ***P*** | ***P* for trend** **^a^** |
| **M10** |  |  |  |  |  |  |  |  |  |  |
| Continuous |  | 3,048/614,288 (4.96) | 0.98 (0.97, 0.98) | < 0.001 |  |  | 563/614,288 (0.92) | 0.98 (0.97, 0.99) | < 0.001 |  |
| Categories |  |  |  |  | < 0.001 |  |  |  |  | < 0.001 |
| Tertile 1 |  | 1,495/210,805 (7.09) | *Reference* | - |  |  | 294/210,805 (1.39) | *Reference* | - |  |
| Tertile 2 |  | 911/205,767 (4.43) | 0.75 (0.69, 0.82) | < 0.001 |  |  | 172/205,767 (0.84) | 0.78 (0.64, 0.95) | 0.016 |  |
| Tertile 3 |  | 642/197,717 (3.25) | 0.66 (0.59, 0.75) | < 0.001 |  |  | 97/197,717 (0.49) | 0.59 (0.44, 0.78) | < 0.001 |  |
| **L5** |  |  |  |  |  |  |  |  |  |  |
| Continuous |  | 3,048/614,288 (4.96) | 1.06 (1.04, 1.09) | < 0.001 |  |  | 563/614,288 (0.92) | 1.12 (1.07, 1.17) | < 0.001 |  |
| Categories |  |  |  |  | < 0.001 |  |  |  |  | 0.001 |
| Tertile 1 |  | 873/203,086 (4.30) | *Reference* | - |  |  | 135/203,086 (0.66) | *Reference* | - |  |
| Tertile 2 |  | 938/204,111 (4.60) | 1.03 (0.94, 1.13) | 0.490 |  |  | 172/204,111 (0.84) | 1.18 (0.94, 1.48) | 0.154 |  |
| Tertile 3 |  | 1,237/207,090 (5.97) | 1.23 (1.12, 1.35) | < 0.001 |  |  | 256/207,090 (1.24) | 1.47 (1.17, 1.85) | 0.001 |  |
| **M10 onset time** |  |  |  |  |  |  |  |  |  |  |
| Categories |  |  |  |  | - |  |  |  |  | - |
| Tertile 1 |  | 993/206,423 (4.81) | 1.04 (0.95, 1.14) | 0.391 |  |  | 156/206,423 (0.76) | 1.37 (1.11, 1.69) | 0.003 |  |
| Tertile 2 |  | 992/203,716 (4.87) | *Reference* | - |  |  | 209/203,716 (1.03) | *Reference* | - |  |
| Tertile 3 |  | 1,063/204,149 (5.21) | 1.02 (0.94, 1.11) | 0.626 |  |  | 198/204,149 (0.97) | 1.17 (0.95, 1.45) | 0.137 |  |
| **L5 onset time** |  |  |  |  |  |  |  |  |  |  |
| Categories |  |  |  |  | - |  |  |  |  | - |
| Tertile 1 |  | 949/203,970 (4.65) | 1.06 (0.97, 1.16) | 0.214 |  |  | 179/203,970 (0.88) | 1.12 (0.91, 1.37) | 0.289 |  |
| Tertile 2 |  | 1,000/203,191 (4.92) | *Reference* | - |  |  | 197/203,191 (0.97) | *Reference* | - |  |
| Tertile 3 |  | 1,099/207,127 (5.31) | 1.07 (0.98, 1.16) | 0.153 |  |  | 187/207,127 (0.90) | 0.94 (0.77, 1.16) | 0.574 |  |

**^a^** *P* for trend was calculated using multivariable Cox regression models to evaluate the differences in the medians of continuous variables across different tertile groups.

Model was adjusted for age, sex, race, education level, TDI, BMI, smoking status, drinking status, healthy diet score, shift work, hypertension, diabetes, hyperlipidemia, MVPA, sleep efficiency, sleep duration, and season of accelerometer wear.

**Abbreviations:** CVD, cardiovascular disease; CKM, cardiovascular-kidney-metabolic syndrom; CRAR, circadian rest-activity rhythm; PYs, person-years; HR, hazard ratio; CI, confidence interval; M10, the most active 10-h period; L5, the least active 5-h period; TDI, Townsend deprivation index; BMI, body mass index; MVPA, moderate to vigorous physical activity.

**Table S16.** Associations between circadian rest-activity rhythm and the risk of all-cause and CVD mortality in patients with CKM stages 1-4 from the NHANES.

| **CRAR** |  | **All-cause mortality** | | | |  | **CVD mortality** | | | |
| --- | --- | --- | --- | --- | --- | --- | --- | --- | --- | --- |
|  |  | **Events/PYs (‰)** | **HR (95% CI)** | ***P*** | ***P* for trend** **^b^** |  | **Events/PYs (‰)** | **HR (95% CI)** | ***P*** | ***P* for trend** **^b^** |
| **RA** |  |  |  |  |  |  |  |  |  |  |
| Continuous **^a^** |  | 438/38,283 (11.44) | 0.78 (0.72, 0.84) | < 0.001 |  |  | 123/38,283 (3.21) | 0.83 (0.73, 0.94) | 0.005 |  |
| Categories |  |  |  |  | < 0.001 |  |  |  |  | < 0.001 |
| Tertile 1 |  | 217/12,071 (17.98) | *Reference* | - |  |  | 64/12,071 (5.30) | *Reference* | - |  |
| Tertile 2 |  | 128/12,288 (10.42) | 0.50 (0.38, 0.67) | < 0.001 |  |  | 42/12,288 (3.42) | 0.69 (0.40, 1.18) | 0.179 |  |
| Tertile 3 |  | 93/13,925 (6.68) | 0.37 (0.26, 0.53) | < 0.001 |  |  | 17/13,925 (1.22) | 0.34 (0.16, 0.70) | 0.004 |  |
| **IS** |  |  |  |  |  |  |  |  |  |  |
| Continuous **^a^** |  | 438/38,283 (11.44) | 0.86 (0.80, 0.93) | < 0.001 |  |  | 123/38,283 (3.21) | 0.89 (0.77, 1.04) | 0.146 |  |
| Categories |  |  |  |  | 0.002 |  |  |  |  | 0.326 |
| Tertile 1 |  | 176/12,058 (14.60) | *Reference* | - |  |  | 46/12,058 (3.81) | *Reference* | - |  |
| Tertile 2 |  | 137/12,692 (10.79) | 0.81 (0.58, 1.11) | 0.192 |  |  | 43/12,692 (3.39) | 0.87 (0.60, 1.27) | 0.471 |  |
| Tertile 3 |  | 125/13,534 (9.24) | 0.70 (0.55, 0.89) | 0.004 |  |  | 34/13,534 (2.51) | 0.77 (0.45, 1.34) | 0.360 |  |
| **IV** |  |  |  |  |  |  |  |  |  |  |
| Continuous **^a^** |  | 438/38,283 (11.44) | 1.14 (1.09, 1.20) | < 0.001 |  |  | 123/38,283 (3.21) | 1.11 (1.03, 1.20) | 0.011 |  |
| Categories |  |  |  |  | < 0.001 |  |  |  |  | 0.296 |
| Tertile 1 |  | 120/12,552 (9.56) | *Reference* | - |  |  | 38/12,552 (3.03) | *Reference* | - |  |
| Tertile 2 |  | 124/12,922 (9.60) | 1.20 (0.88, 1.64) | 0.247 |  |  | 34/12,922 (2.63) | 1.00 (0.47, 2.16) | 0.992 |  |
| Tertile 3 |  | 194/12,809 (15.15) | 1.53 (1.19, 1.95) | 0.001 |  |  | 51/12,809 (3.98) | 1.30 (0.71, 2.37) | 0.394 |  |
| **M10** |  |  |  |  |  |  |  |  |  |  |
| Continuous |  | 438/38,283 (11.44) | 0.89 (0.85, 0.92) | < 0.001 |  |  | 123/38,283 (3.21) | 0.87 (0.80, 0.95) | 0.003 |  |
| Categories |  |  |  |  | < 0.001 |  |  |  |  | 0.014 |
| Tertile 1 |  | 227/12,568 (18.06) | *Reference* | - |  |  | 62/12,568 (4.93) | *Reference* | - |  |
| Tertile 2 |  | 117/12,893 (9.07) | 0.68 (0.52, 0.88) | 0.004 |  |  | 33/12,893 (2.56) | 0.78 (0.51, 1.20) | 0.261 |  |
| Tertile 3 |  | 94/12,822 (7.33) | 0.52 (0.36, 0.76) | 0.001 |  |  | 28/12,822 (2.18) | 0.52 (0.24, 1.11) | 0.095 |  |
| **L5** |  |  |  |  |  |  |  |  |  |  |
| Continuous |  | 438/38,283 (11.44) | 1.17 (1.06, 1.29) | 0.002 |  |  | 123/38,283 (3.21) | 1.03 (0.89, 1.20) | 0.659 |  |
| Categories |  |  |  |  | < 0.001 |  |  |  |  | 0.018 |
| Tertile 1 |  | 114/12,450 (9.16) | *Reference* | - |  |  | 24/12,450 (1.93) | *Reference* | - |  |
| Tertile 2 |  | 129/13,008 (9.92) | 0.93 (0.69, 1.26) | 0.651 |  |  | 40/13,008 (3.08) | 1.27 (0.65, 2.50) | 0.484 |  |
| Tertile 3 |  | 195/12,826 (15.20) | 1.64 (1.18, 2.27) | 0.003 |  |  | 59/12,826 (4.60) | 1.81 (0.93, 3.51) | 0.082 |  |
| **M10 onset time** |  |  |  |  |  |  |  |  |  |  |
| Categories |  |  |  |  | - |  |  |  |  | - |
| Tertile 1 |  | 150/12,529 (11.97) | 1.23 (0.89, 1.71) | 0.213 |  |  | 43/12,529 (3.43) | 1.17 (0.58, 2.37) | 0.667 |  |
| Tertile 2 |  | 144/12,962 (11.11) | *Reference* | - |  |  | 39/12,962 (3.01) | *Reference* | - |  |
| Tertile 3 |  | 144/12,793 (11.26) | 0.96 (0.70, 1.33) | 0.823 |  |  | 41/12,793 (3.21) | 1.04 (0.63, 1.72) | 0.868 |  |
| **L5 onset time** |  |  |  |  |  |  |  |  |  |  |
| Categories |  |  |  |  | - |  |  |  |  | - |
| Tertile 1 |  | 138/12,621 (10.86) | 1.11 (0.76, 1.61) | 0.586 |  |  | 38/12,621 (3.01) | 0.73 (0.39, 1.40) | 0.347 |  |
| Tertile 2 |  | 148/12,780 (11.66) | *Reference* | - |  |  | 42/12,780 (3.29) | *Reference* | - |  |
| Tertile 3 |  | 152/12,883 (11.80) | 1.18 (0.87, 1.61) | 0.297 |  |  | 43/12,883 (3.34) | 1.06 (0.57, 1.95) | 0.862 |  |

**^a^** Since these continuous variable has a narrow range (1-2), it was scaled by a factor of ten for the analysis. The HR reflects the hazard ratio for a 0.1 unit change in the variable.

**^b^** *P* for trend was calculated using multivariable Cox regression models to evaluate the differences in the medians of continuous variables across different tertile groups.

Models were adjusted for age, sex, race, education level, PIR, BMI, smoking status, drinking status, hypertension, diabetes, hyperlipidemia, depression, cancer and MVPA.

**Abbreviations:** CVD, cardiovascular disease; CKM, cardiovascular-kidney-metabolic syndrom; NHANES, National Health and Nutrition Examination Survey; CRAR, circadian rest-activity rhythm; PYs, person-years; HR, hazard ratio; CI, confidence interval; RA, relative amplitude; IS, interdaily stability; IV, intradaily variability; M10, the most active 10-h period; L5, the least active 5-h period; PIR, poverty-to-income ratio; BMI, body mass index; MVPA, moderate to vigorous physical activity.

**Table S17.** Multicollinearity evaluation of predictor variables for CVD incidence in individuals with CKM stages 0-3 through variance inflation factor (VIF) analysis.

| **Feature** | **VIF** |
| --- | --- |
| RA | 1.223 |
| Age | 1.585 |
| Sex | 1.084 |
| BMI | 1.248 |
| SBP | 1.215 |
| HbA1c | 1.098 |
| eGFR | 1.415 |
| MVPA | 1.280 |

**Abbreviations:** CVD, cardiovascular disease; CKM, cardiovascular-kidney-metabolic syndrom; RA, relative amplitude; BMI, body mass index; SBP, systolic blood pressure; HbA1c, glycated hemoglobin; eGFR, estimated glomerular filtration rate; MVPA, moderate to vigorous physical activity.

**Table S18.** Multicollinearity evaluation of predictor variables for all-cause mortality in patients with CKM stages 1-4 through variance inflation factor (VIF) analysis.

| **Feature** | **VIF** |
| --- | --- |
| RA | 2.338 |
| IS | 1.235 |
| L5 | 2.201 |
| Age | 1.589 |
| Sex | 1.104 |
| TDI | 1.035 |
| SBP | 1.111 |
| HbA1c | 1.069 |
| Total cholesterol | 1.053 |
| eGFR | 1.342 |
| Cancer | 1.020 |

**Abbreviations:** CKM, cardiovascular-kidney-metabolic syndrom; RA, relative amplitude; IS, interdaily stability; L5, the least active 5-h period; TDI, Townsend deprivation index; SBP, systolic blood pressure; HbA1c, glycated hemoglobin; eGFR, estimated glomerular filtration rate.

**Table S19.** Multicollinearity evaluation of predictor variables for CVD mortality in patients with CKM stages 1-4 through variance inflation factor (VIF) analysis.

| **Feature** | **VIF** |
| --- | --- |
| RA | 1.074 |
| M10 onset time | 1.063 |
| Age | 1.468 |
| Sex | 1.025 |
| SBP | 1.102 |
| HbA1c | 1.056 |
| eGFR | 1.333 |

**Abbreviations:** CVD, cardiovascular disease; CKM, cardiovascular-kidney-metabolic syndrom; RA, relative amplitude; M10, the most active 10-h period; SBP, systolic blood pressure; HbA1c, glycated hemoglobin; eGFR, estimated glomerular filtration rate.

**Table S20.** Additional evaluation Metrics for incremental predictive value of circadian rest-activity rhythm for CVD incidence, all-cause and CVD mortality in individuals with CKM.

|  |  | **AUC (95% CI)** | **ΔAUC (95% CI)** | **DeLong *P*** |  | **AIC** | **BIC** | **LRT *P*** |
| --- | --- | --- | --- | --- | --- | --- | --- | --- |
| **CVD incidence** **^a^** |  |  |  |  |  |  |  |  |
| Basic model |  | 0.701 (0.695, 0.707) | *Reference* |  |  | 145,249.21 | 145,296.96 |  |
| Basic model + CRAR |  | 0.702 (0.696, 0.708) | 0.001 (0.000, 0.002) | 0.008 |  | 145,198.79 | 145,253.36 | < 0.001 |
| **All-cause mortality** **^b^** |  |  |  |  |  |  |  |  |
| Basic model |  | 0.732 (0.723, 0.741) | *Reference* |  |  | 65,360.12 | 65,408.30 |  |
| Basic model + CRAR |  | 0.751 (0.742, 0.760) | 0.019 (0.015, 0.023) | < 0.001 |  | 64,878.10 | 64,944.35 | < 0.001 |
| **CVD mortality** **^c^** |  |  |  |  |  |  |  |  |
| Basic model |  | 0.777 (0.759, 0.796) | *Reference* |  |  | 11,797.29 | 11,818.96 |  |
| Basic model + CRAR |  | 0.794 (0.776, 0.812) | 0.017 (0.011, 0.023) | < 0.001 |  | 11,680.57 | 11,710.91 | < 0.001 |

**^a^** The population for predicting CVD incidence includes participants in CKM stages 0-3. The basic model incorporates variables including age, sex, BMI, SBP, HbA1c, eGFR, and MVPA. The incremental model builds upon the base model by adding CRAR feature, specifically RA.

**^b^** The population for predicting all-cause mortality includes participants in CKM stages 1-4. The basic model incorporates variables including age, sex, TDI, SBP, HbA1c, total cholesterol, eGFR, and cancer. The incremental model builds upon the base model by adding CRAR features, specifically RA, IS, and L5.

**^c^** The population for predicting CVD mortality includes participants in CKM stages 1-4. The basic model incorporates variables including age, sex, SBP, HbA1c, and eGFR. The incremental model builds upon the base model by adding CRAR feature, specifically RA and M10 onset time.

**Abbreviations:** CVD, cardiovascular disease; CKM, cardiovascular-kidney-metabolic syndrom; CRAR, circadian rest-activity rhythm; CI, confidence interval; AUC, area under the curve; AIC, Akaike information criterion; BIC, Bayesian information criterion; LRT, likelihood ratio test; BMI, body mass index; TDI, Townsend deprivation index; SBP, systolic blood pressure; HbA1c, glycated hemoglobin; eGFR, estimated glomerular filtration rate; MVPA, moderate to vigorous physical activity; RA, relative amplitude; IS, interdaily stability; L5, the least active 5-h period; M10, the most active 10-h period.

***Supplementary Figures***

**
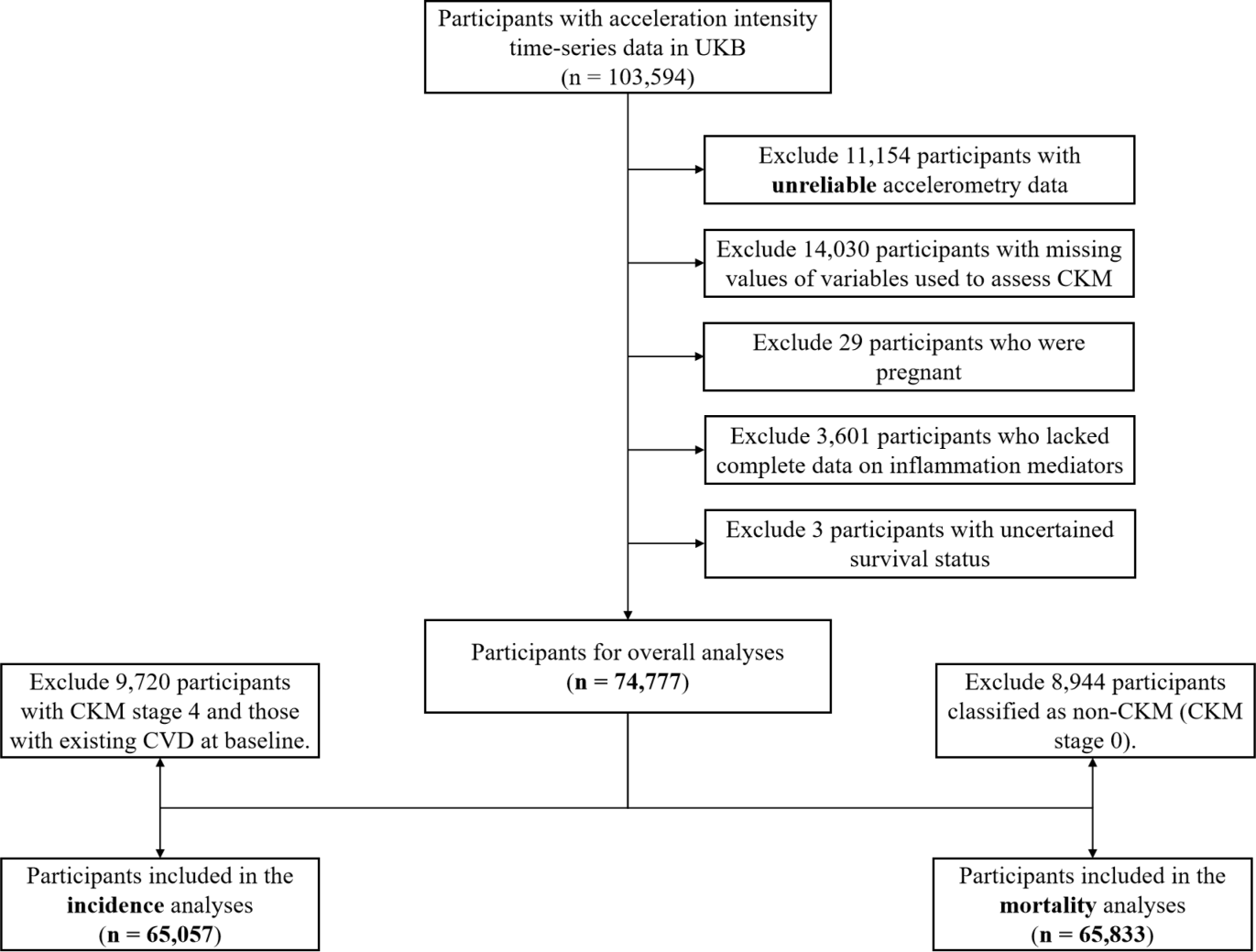
**

**Figure S1**. Flowchart of participants selection in the UKB cohort.

**Abbreviations:** UKB, UK Biobank; CKM, cardiovascular-kidney-metabolic syndrom; CVD, cardiovascular disease.

**
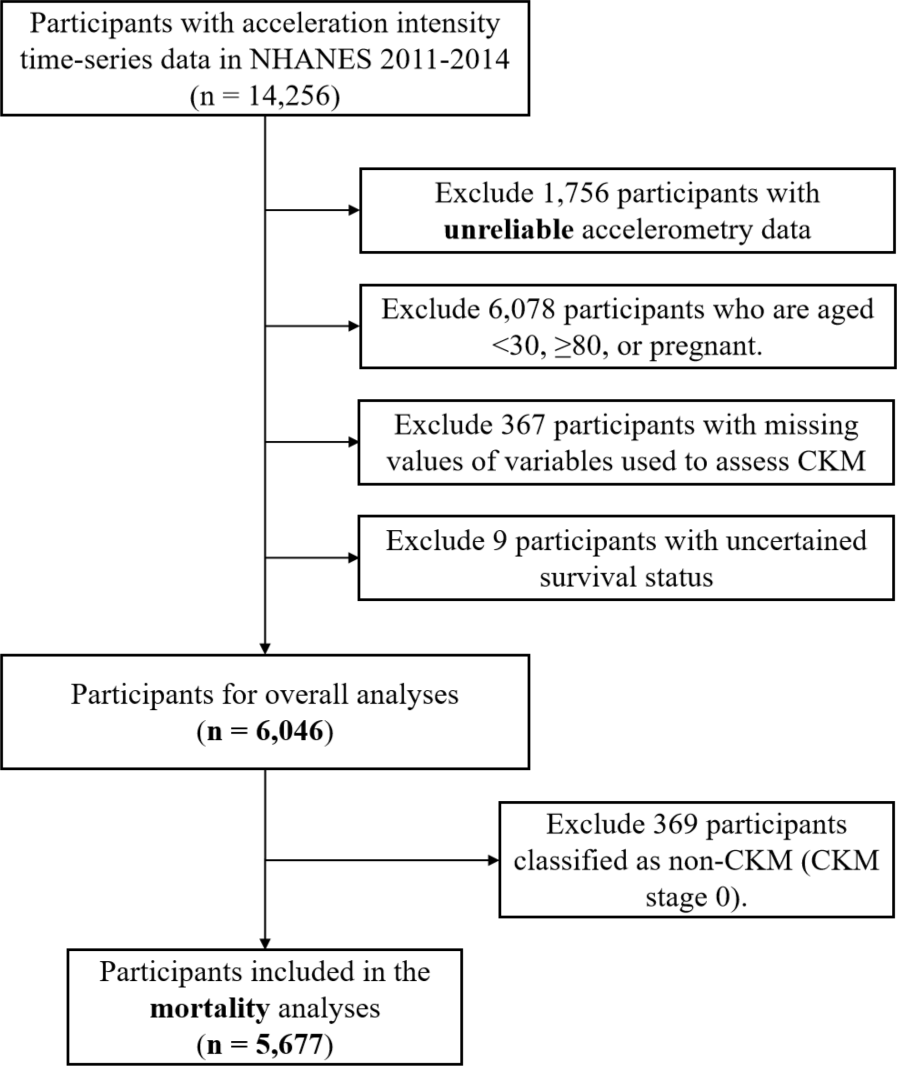
**

**Figure S2**. Flowchart of participants selection in the NHANES cohort.

**Abbreviations:** NHANES, National Health and Nutrition Examination Survey; CKM, cardiovascular-kidney-metabolic syndrom.


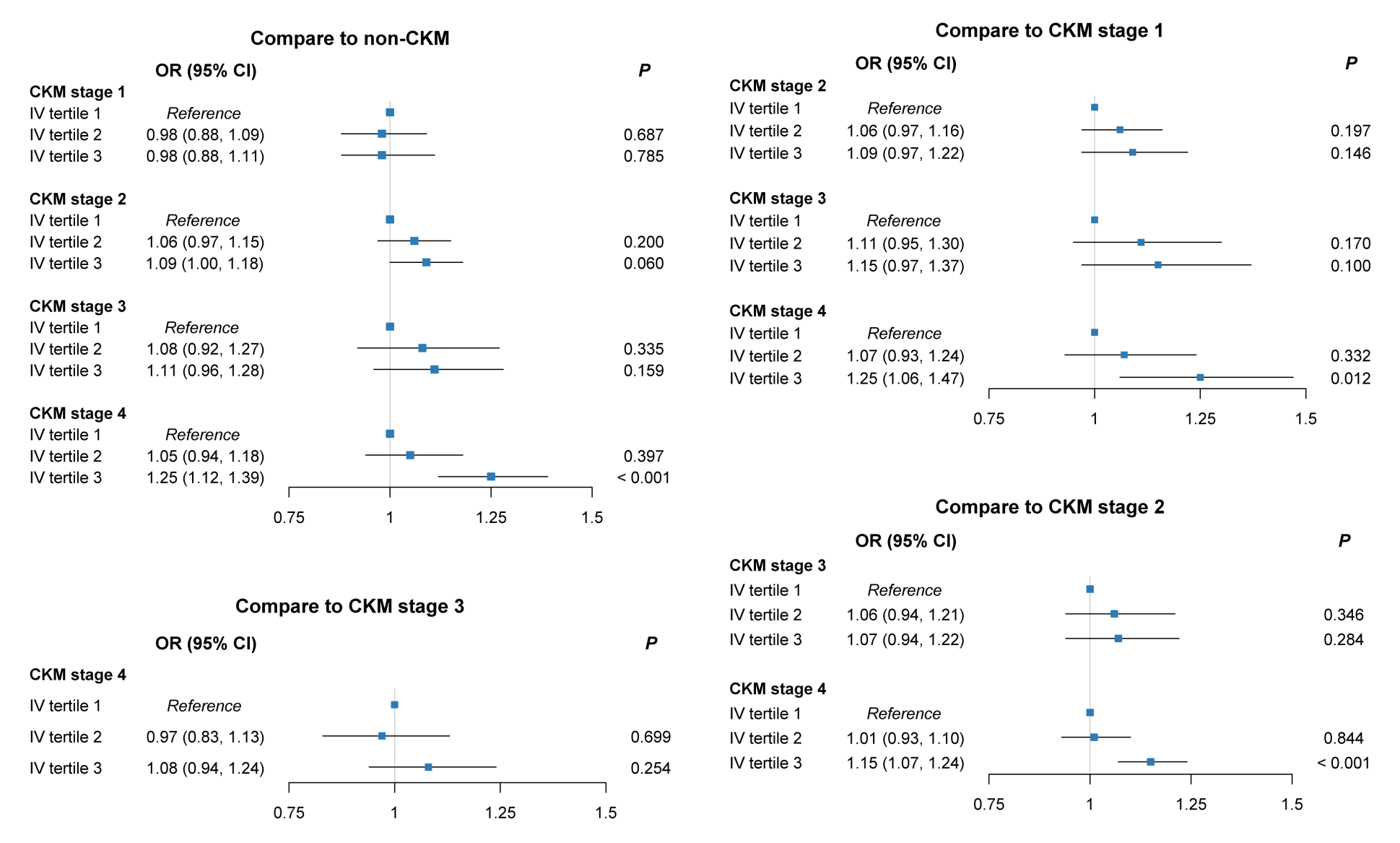


**Figure S3**. Association between IV tertiles and CKM staging (stages 0-4).

*P* values from multinomial logistic regression models adjusted for age, sex, race, education, TDI, BMI, smoking status, drinking status, healthy diet score, shift work, SBP, HbA1c, total cholesterol, MVPA, sleep duration, sleep efficiency, and season of accelerometer wear.

**Abbreviations:** CKM, cardiovascular-kidney-metabolic syndrome; IV, intradaily variability; OR, odds ratio; CI, confidence interval; TDI, Townsend deprivation index; BMI, body mass index; SBP, systolic blood pressure; HbA1c, glycated hemoglobin; MVPA, moderate to vigorous physical activity.


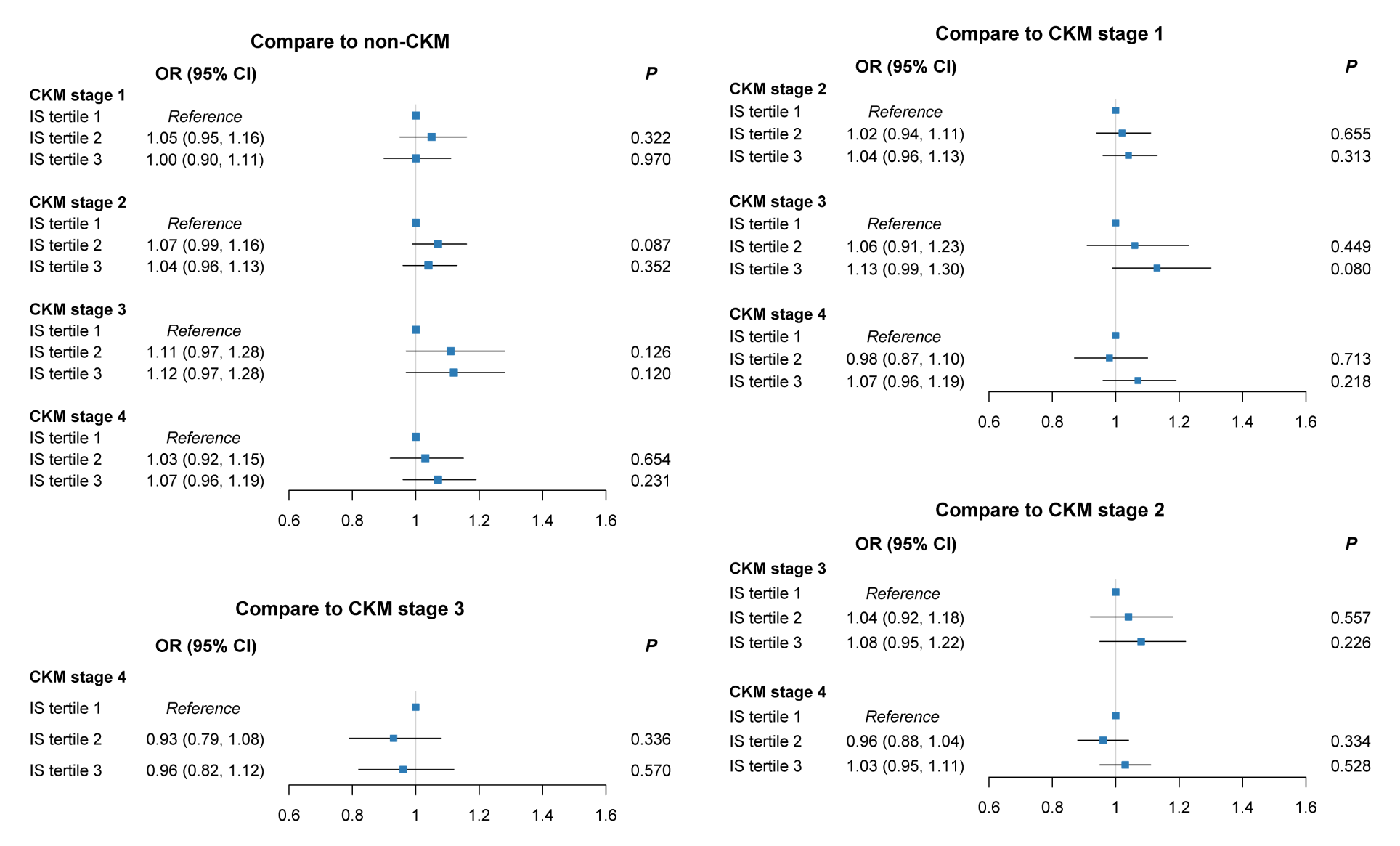


**Figure S4**. Association between IS tertiles and CKM staging (stages 0-4).

*P* values from multinomial logistic regression models adjusted for age, sex, race, education, TDI, BMI, smoking status, drinking status, healthy diet score, shift work, SBP, HbA1c, total cholesterol, MVPA, sleep duration, sleep efficiency, and season of accelerometer wear.

**Abbreviations:** CKM, cardiovascular-kidney-metabolic syndrome; IS, interdaily stability; OR, odds ratio; CI, confidence interval; TDI, Townsend deprivation index; BMI, body mass index; SBP, systolic blood pressure; HbA1c, glycated hemoglobin; MVPA, moderate to vigorous physical activity.


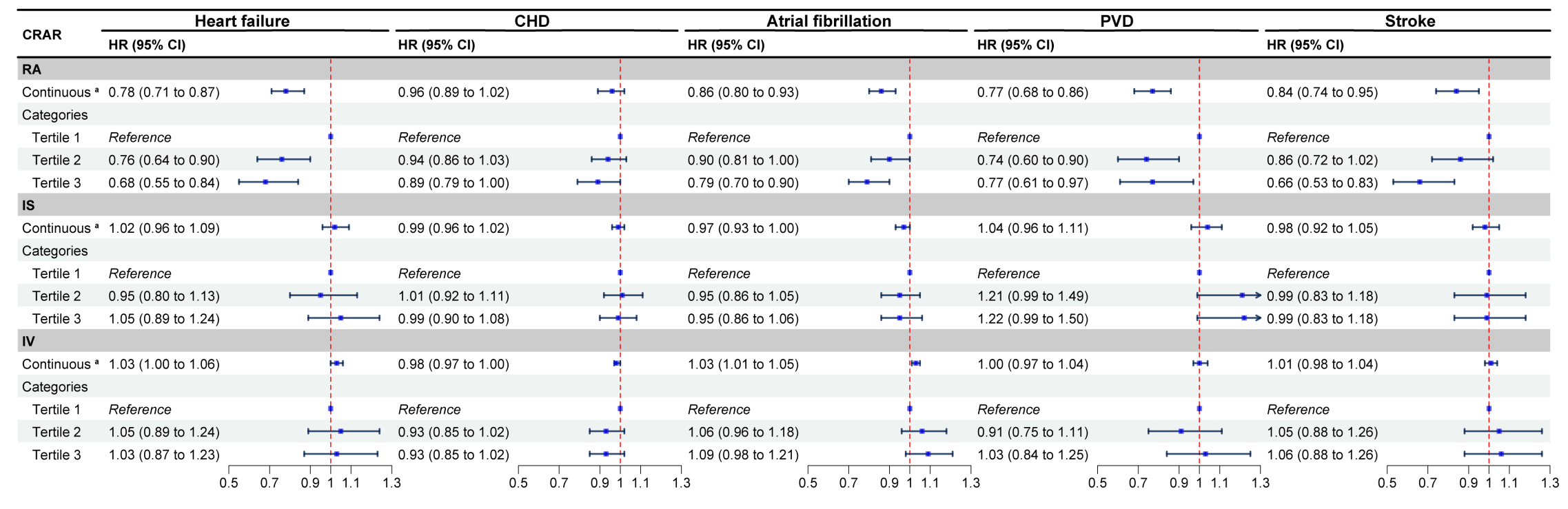


**Figure S5**. Associations between major CRAR and the risk of incidence of five CVD subtypes in individuals with CKM stages 0-3.

**^a^** Since the continuous variable has a narrow range (1-2), it was scaled by a factor of ten for the analysis. The HR reflects the hazard ratio for a 0.1 unit change in the variable.

**Abbreviations:** RA, relative amplitude; IS, interdaily stability; IV, intradaily variability; CHD, coronary heart disease; PVD, peripheral vascular disease; HR, hazard ratio; CI, confidence interval; CRAR, circadian rest-activity rhythm; CVD, cardiovascular disease; CKM, cardiovascular-kidney-metabolic syndrome.
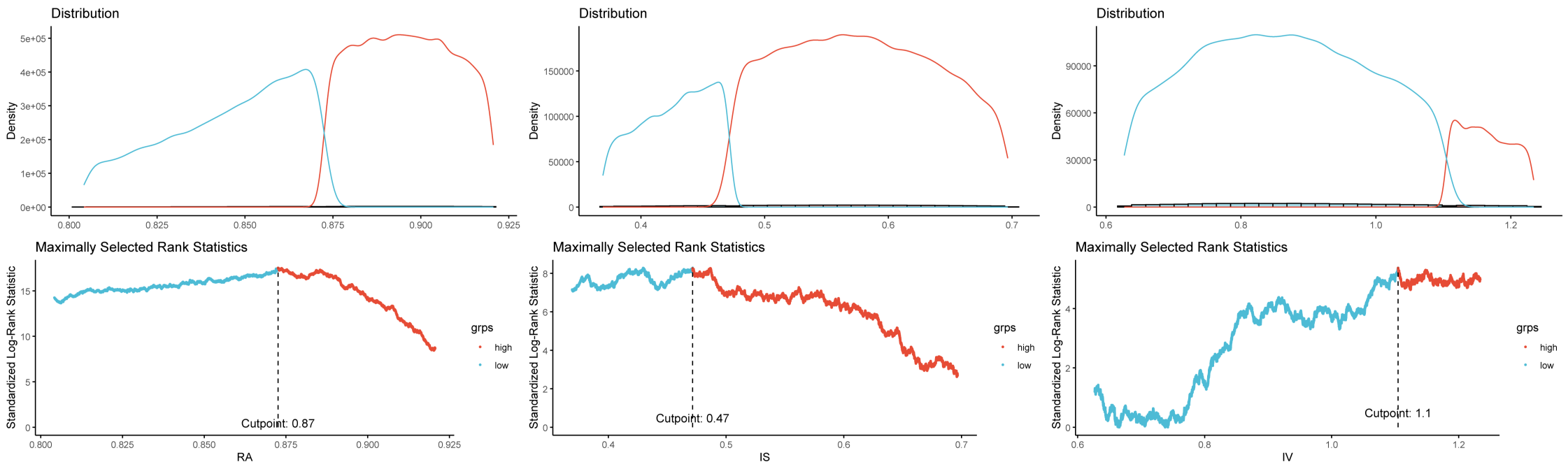


**Figure S6**. Determination of optimal risk stratification cut-off points for major CRAR on CVD incidence in individuals with CKM stages 0-3.

**Abbreviations:** RA, relative amplitude; IS, interdaily stability; IV, intradaily variability; CRAR, circadian rest-activity rhythm; CVD, cardiovascular disease; CKM, cardiovascular-kidney-metabolic syndrome.


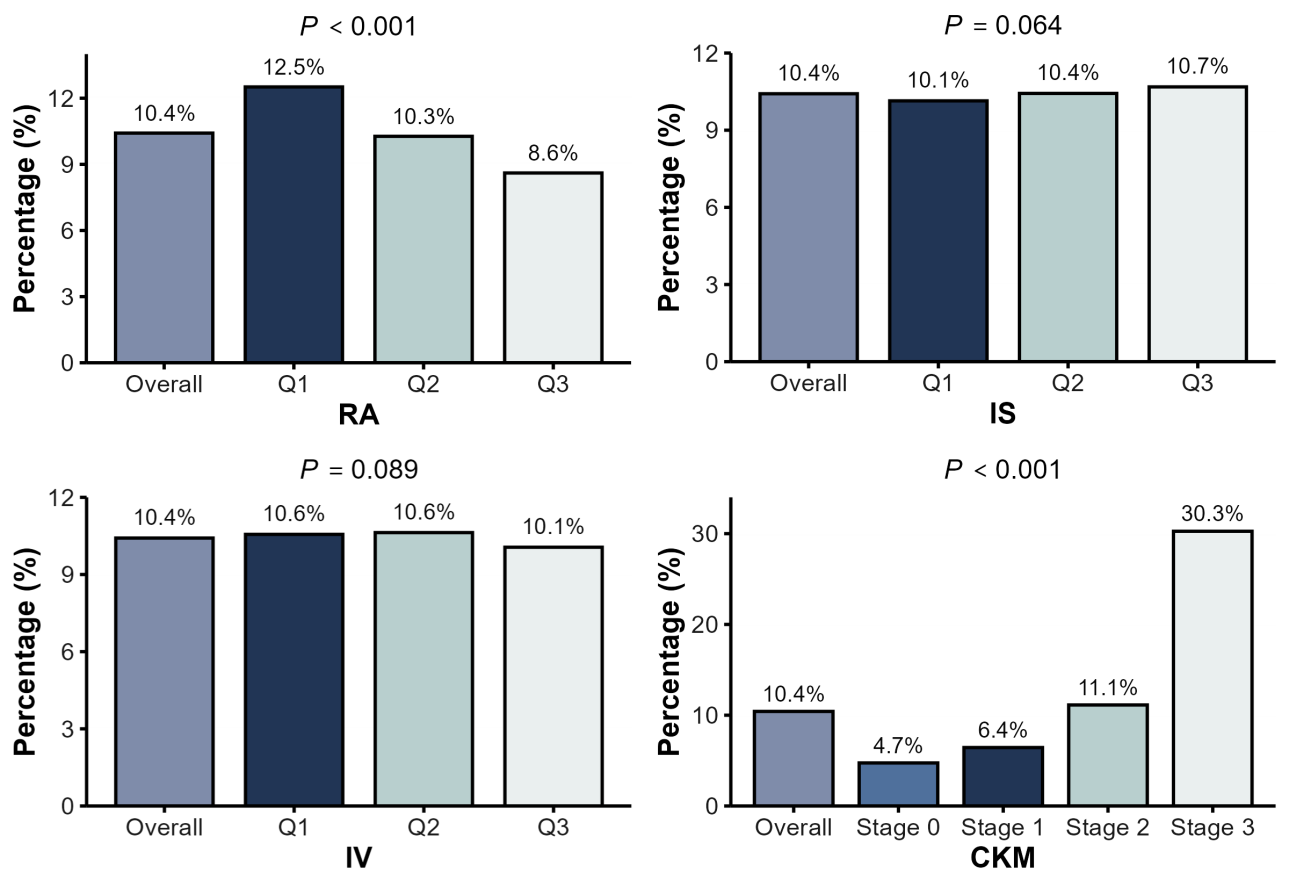


**Figure S7**. Distribution of CVD incidence by major CRAR tertiles and CKM stages in individuals with CKM stages 0-3.

*P* values from the Fisher’s exact test.

**Abbreviations:** RA, relative amplitude; IS, interdaily stability; IV, intradaily variability; CVD, cardiovascular disease; CRAR, circadian rest-activity rhythm; CKM, cardiovascular-kidney-metabolic syndrome.


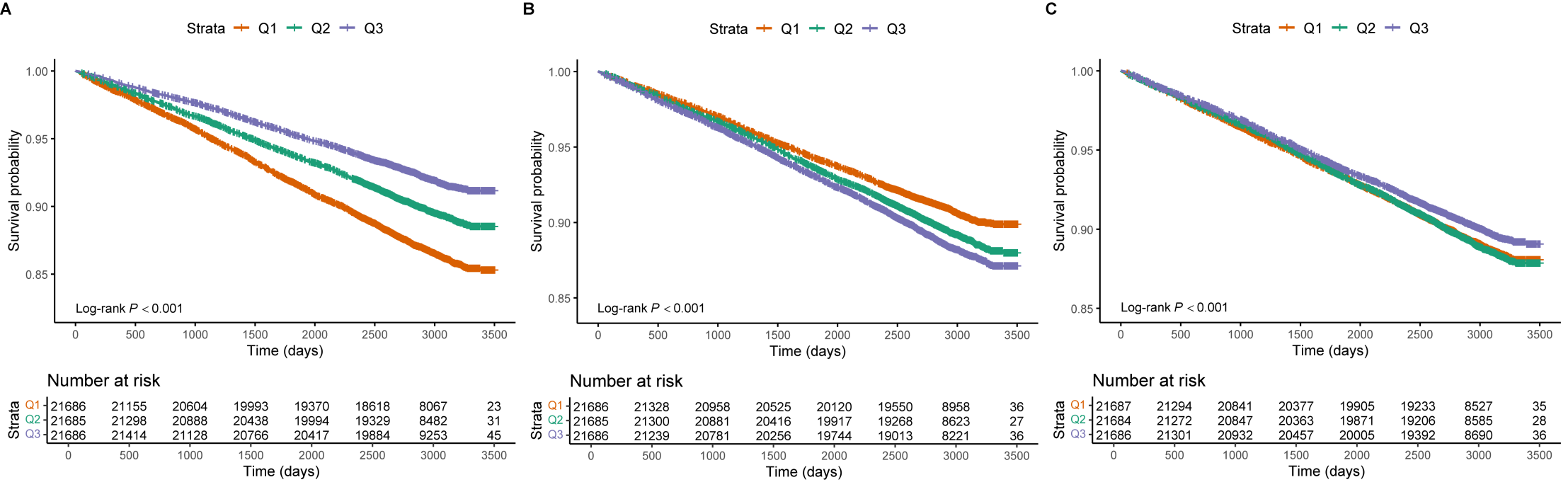


**Figure S8**. Kaplan-Meier survival curves for CVD incidence across major CRAR tertiles in individuals with CKM stages 0-3.

(A) RA, (B) IS, (C) IV.

Log-rank *P* values from log-rank test.

**Abbreviations:** CVD, cardiovascular disease; CRAR, circadian rest-activity rhythm; CKM, cardiovascular-kidney-metabolic syndrome; RA, relative amplitude; IS, interdaily stability; IV, intradaily variability.


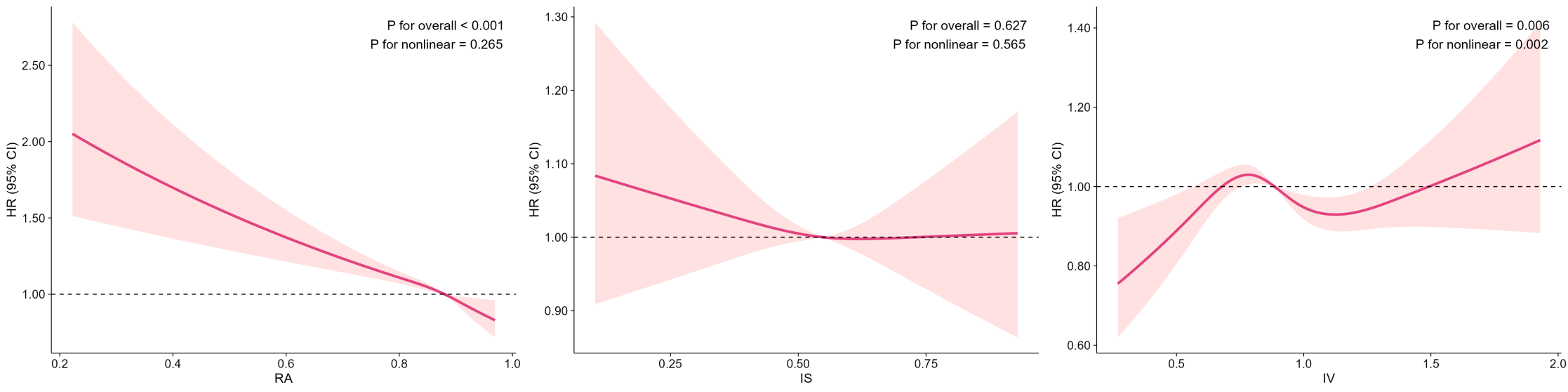


**Figure S9**. Restricted cubic spline analyses for associations between major CRAR and CVD incidence in individuals with CKM stages 0-3.

*P* values from multivariable Cox proportional hazards models adjusted for age, sex, race, education, TDI, BMI, smoking status, drinking status, healthy diet score, shift work, hypertension, diabetes, hyperlipidemia, MVPA, sleep duration, sleep efficiency, and season of accelerometer wear.

**Abbreviations:** RA, relative amplitude; IS, interdaily stability; IV, intradaily variability; HR, hazard ratio; CI, confidence interval; CRAR, circadian rest-activity rhythm; CVD, cardiovascular disease; CKM, cardiovascular-kidney-metabolic syndrome; TDI, Townsend deprivation index; BMI, body mass index; MVPA, moderate to vigorous physical activity.
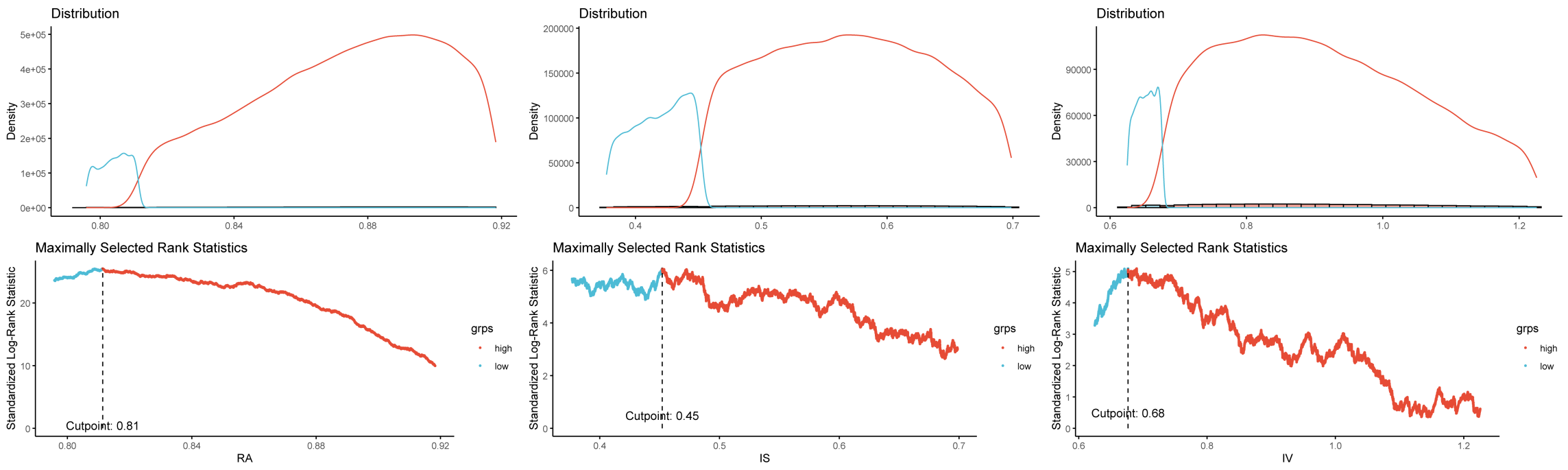


**Figure S10**. Determination of optimal risk stratification cut-off points for major CRAR on all-cause mortality in patients with CKM stages 1-4.

**Abbreviations:** RA, relative amplitude; IS, interdaily stability; IV, intradaily variability; CRAR, circadian rest-activity rhythm; CKM, cardiovascular-kidney-metabolic syndrome.


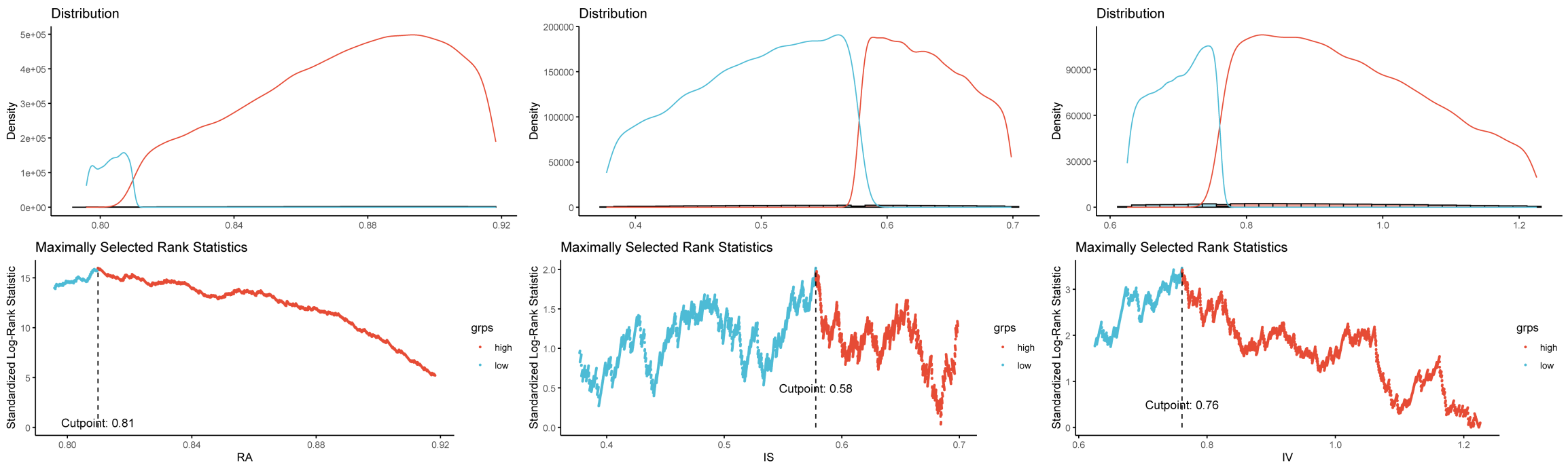


**Figure S11**. Determination of optimal risk stratification cut-off points for major CRAR on CVD mortality in patients with CKM stages 1-4.

**Abbreviations:** RA, relative amplitude; IS, interdaily stability; IV, intradaily variability; CRAR, circadian rest-activity rhythm; CVD, cardiovascular disease; CKM, cardiovascular-kidney-metabolic syndrome.


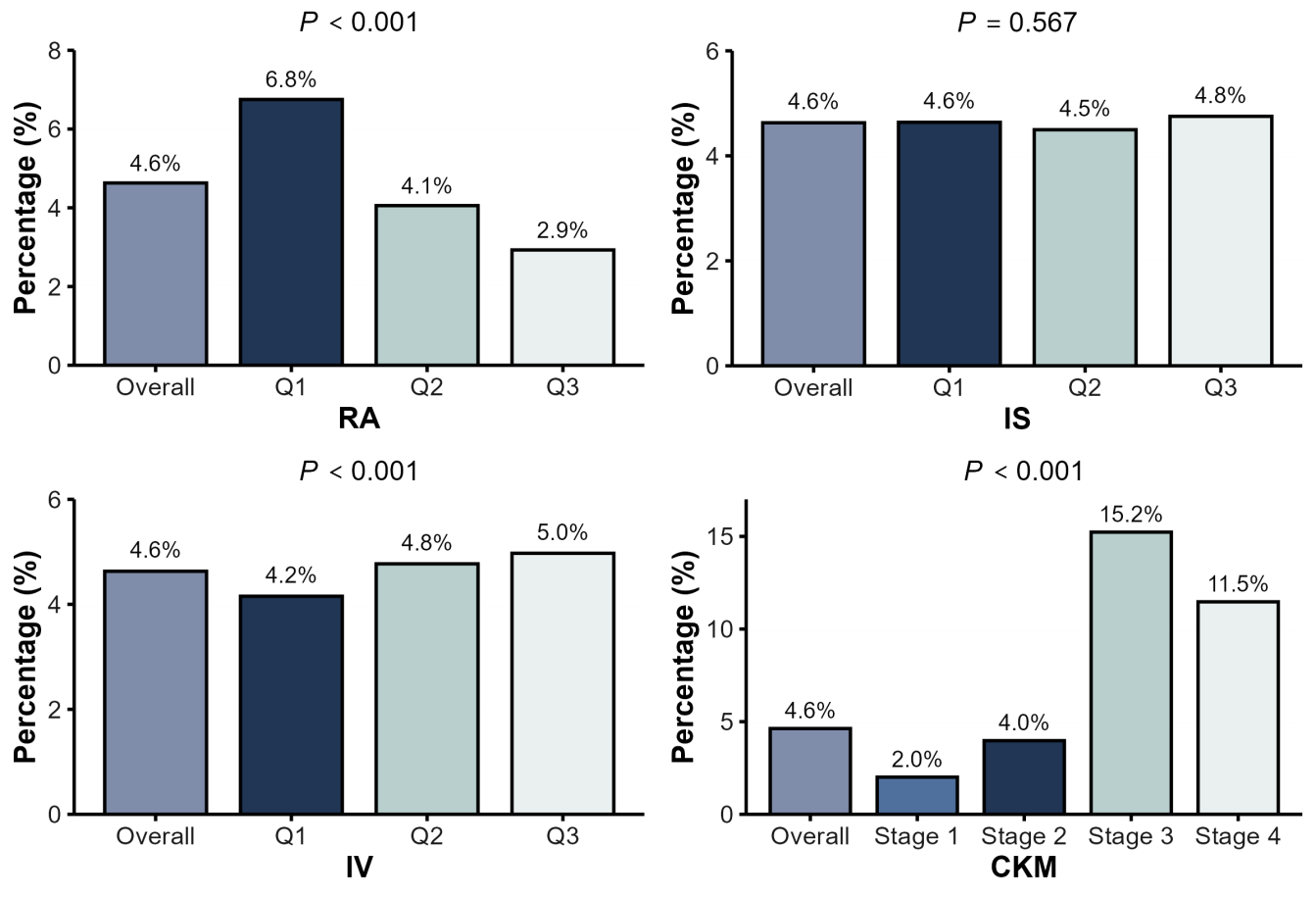


**Figure S12**. Distribution of all-cause mortality by major CRAR tertiles and CKM stages in patients with CKM stages 1-4.

*P* values from the Fisher’s exact test.

**Abbreviations:** RA, relative amplitude; IS, interdaily stability; IV, intradaily variability; CRAR, circadian rest-activity rhythm; CKM, cardiovascular-kidney-metabolic syndrome.


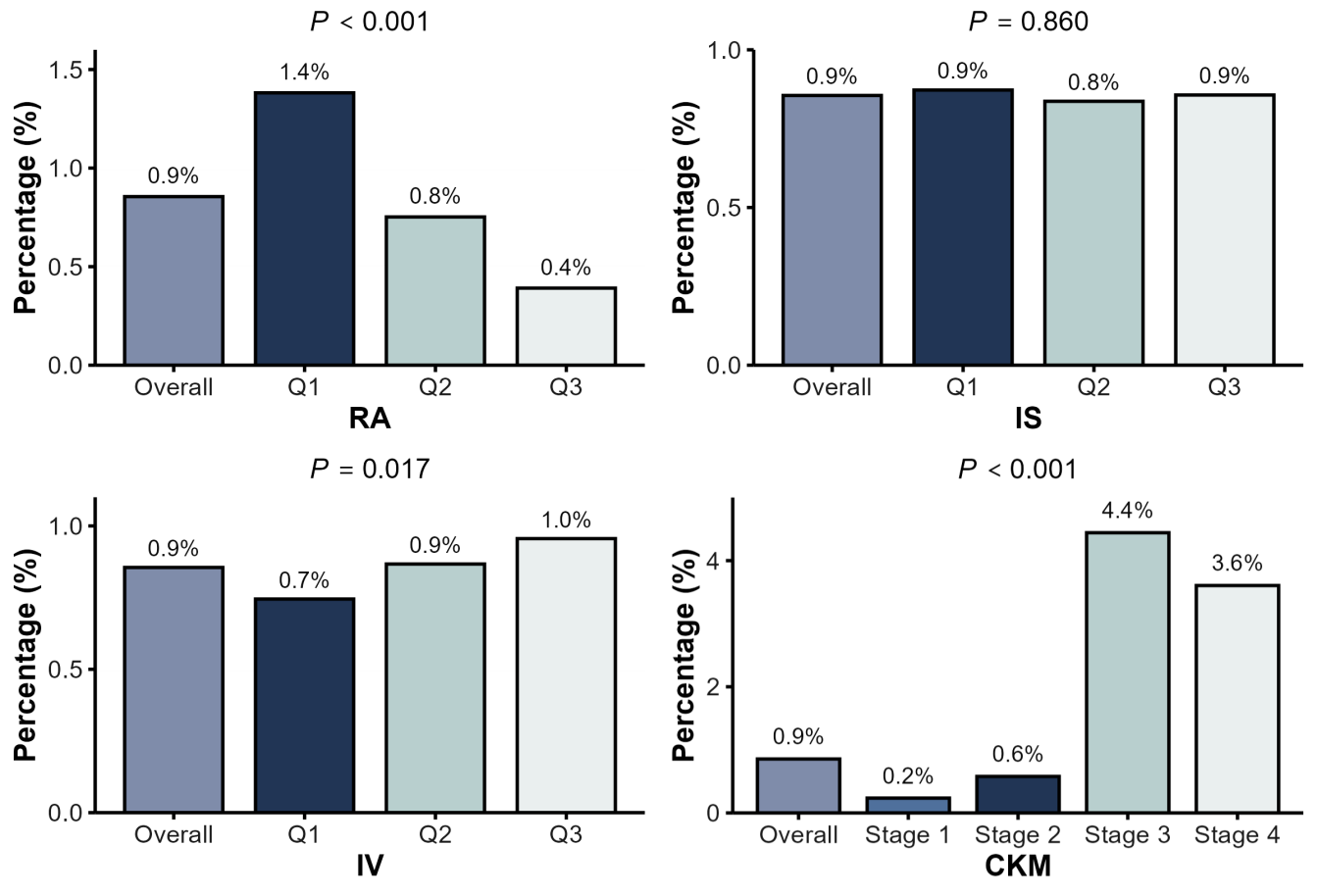


**Figure S13**. Distribution of CVD mortality by major CRAR tertiles and CKM stages in patients with CKM stages 1-4.

*P* values from the Fisher’s exact test.

**Abbreviations:** RA, relative amplitude; IS, interdaily stability; IV, intradaily variability; CVD, cardiovascular disease; CRAR, circadian rest-activity rhythm; CKM, cardiovascular-kidney-metabolic syndrome.


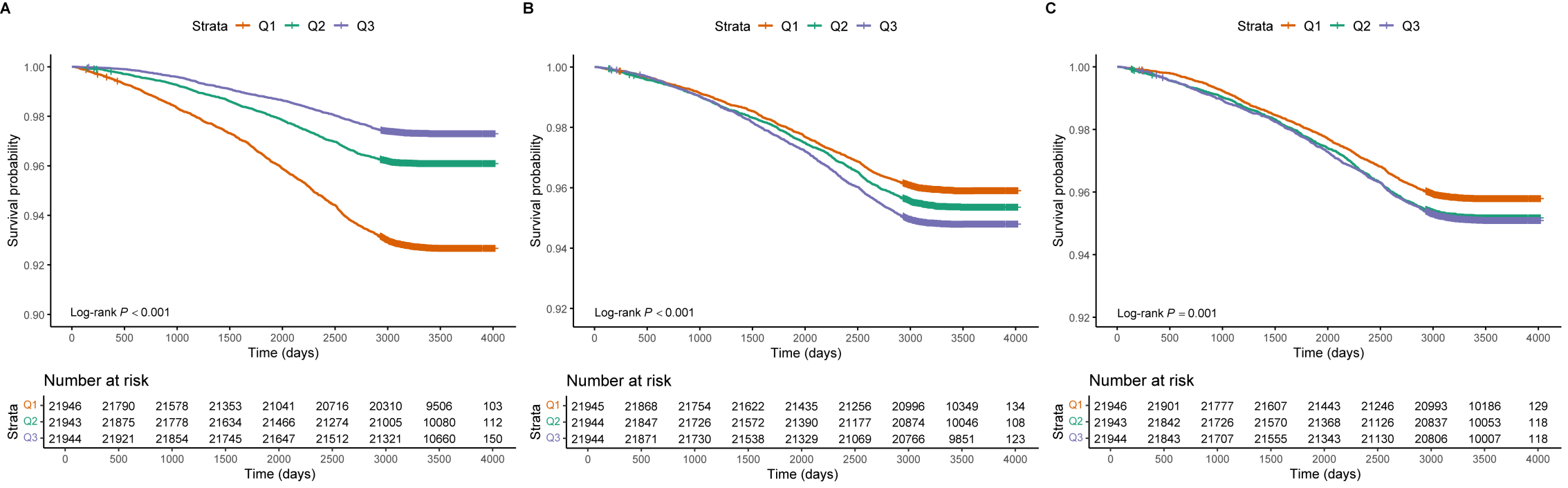


**Figure S14**. Kaplan-Meier survival curves for all-cause mortality across major CRAR tertiles in patients with CKM stages 1-4.

(A) RA, (B) IS, (C) IV.

Log-rank *P* values from log-rank test.

**Abbreviations:** CRAR, circadian rest-activity rhythm; CKM, cardiovascular-kidney-metabolic syndrome; RA, relative amplitude; IS, interdaily stability; IV, intradaily variability.


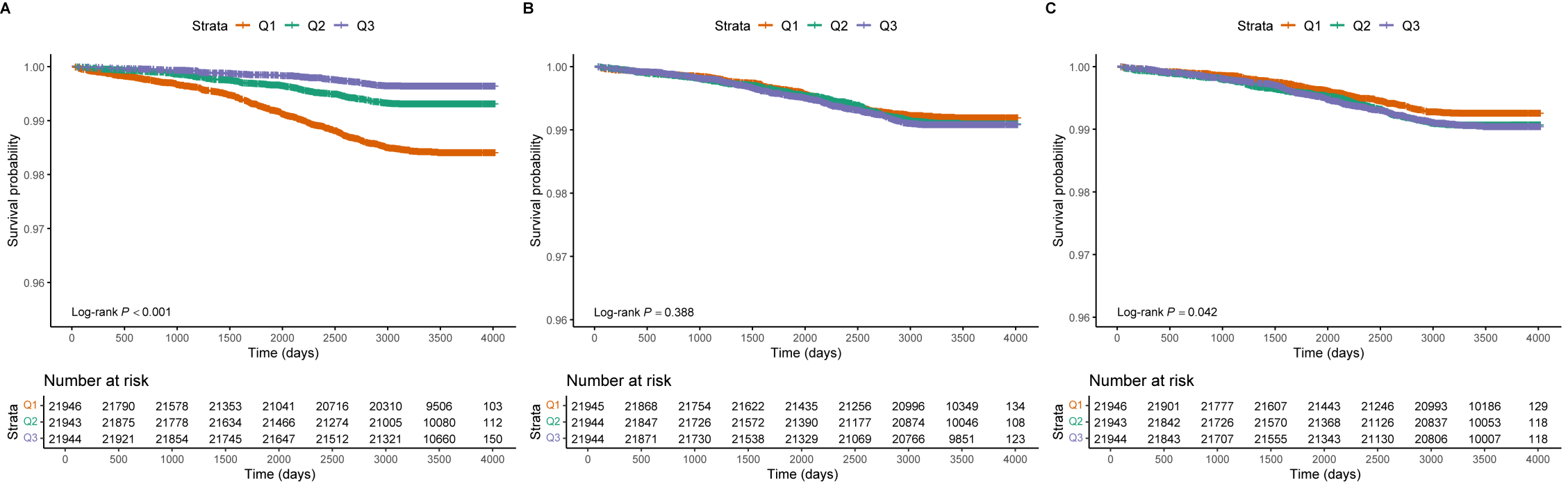


**Figure S15**. Kaplan-Meier survival curves for CVD mortality across major CRAR tertiles in patients with CKM stages 1-4.

(A) RA, (B) IS, (C) IV.

Log-rank *P* values from log-rank test.

**Abbreviations:** CRAR, circadian rest-activity rhythm; CVD, cardiovascular disease; CKM, cardiovascular-kidney-metabolic syndrome; RA, relative amplitude; IS, interdaily stability; IV, intradaily variability.


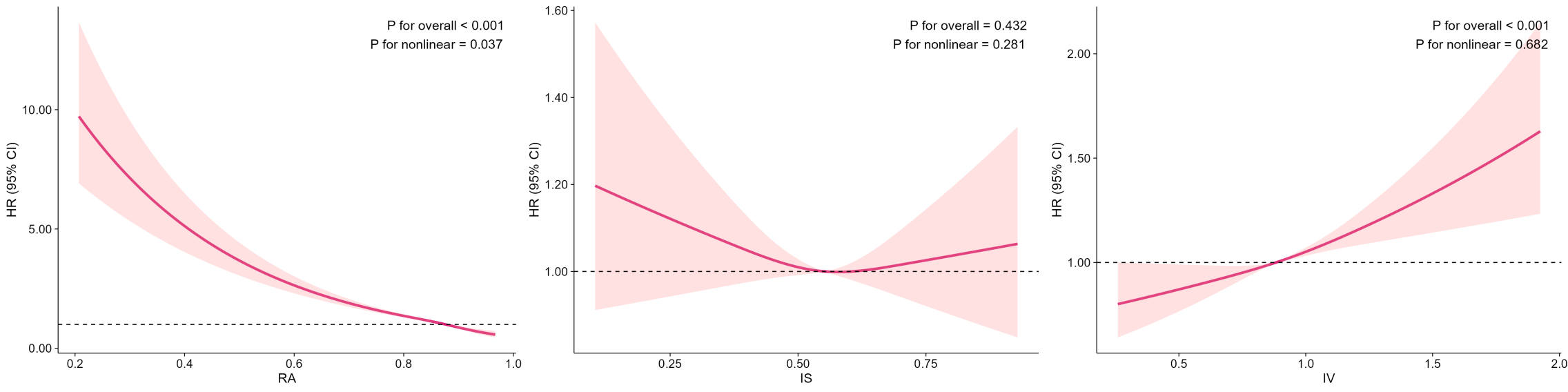


**Figure S16**. Restricted cubic spline analyses for associations between major CRAR and all-cause mortality in patients with CKM stages 1-4.

*P* values from multivariable Cox proportional hazards models adjusted for age, sex, race, education, TDI, BMI, smoking status, drinking status, healthy diet score, shift work, hypertension, diabetes, hyperlipidemia, MVPA, sleep duration, sleep efficiency, and season of accelerometer wear.

**Abbreviations:** RA, relative amplitude; IS, interdaily stability; IV, intradaily variability; HR, hazard ratio; CI, confidence interval; CRAR, circadian rest-activity rhythm; CKM, cardiovascular-kidney-metabolic syndrome; TDI, Townsend deprivation index; BMI, body mass index; MVPA, moderate to vigorous physical activity.


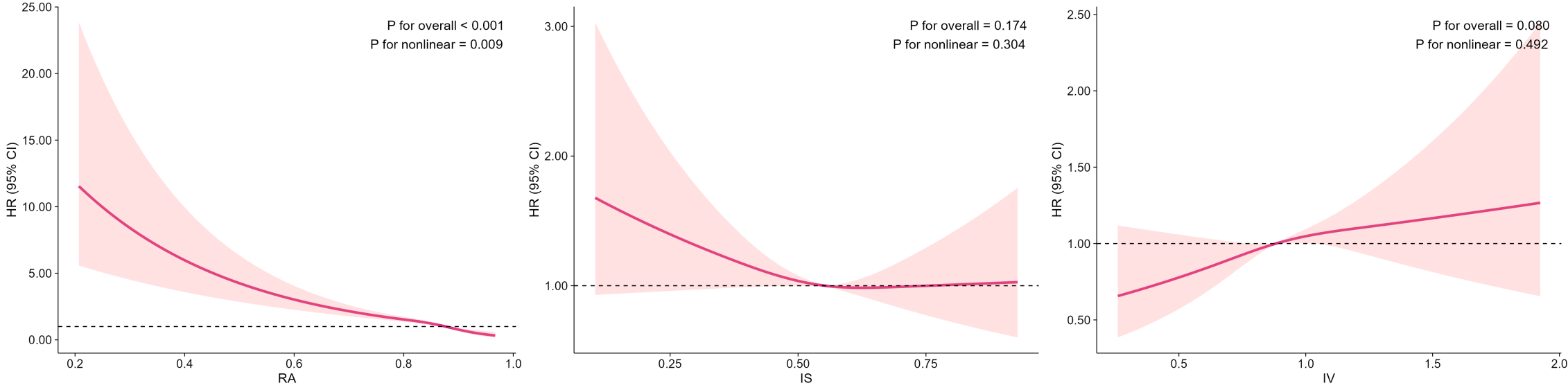


**Figure S17**. Restricted cubic spline analyses for associations between major CRAR and CVD mortality in patients with CKM stages 1-4.

*P* values from multivariable Cox proportional hazards models adjusted for age, sex, race, education, TDI, BMI, smoking status, drinking status, healthy diet score, shift work, hypertension, diabetes, hyperlipidemia, MVPA, sleep duration, sleep efficiency, and season of accelerometer wear.

**Abbreviations:** RA, relative amplitude; IS, interdaily stability; IV, intradaily variability; HR, hazard ratio; CI, confidence interval; CRAR, circadian rest-activity rhythm; CVD, cardiovascular disease; CKM, cardiovascular-kidney-metabolic syndrome; TDI, Townsend deprivation index; BMI, body mass index; MVPA, moderate to vigorous physical activity.


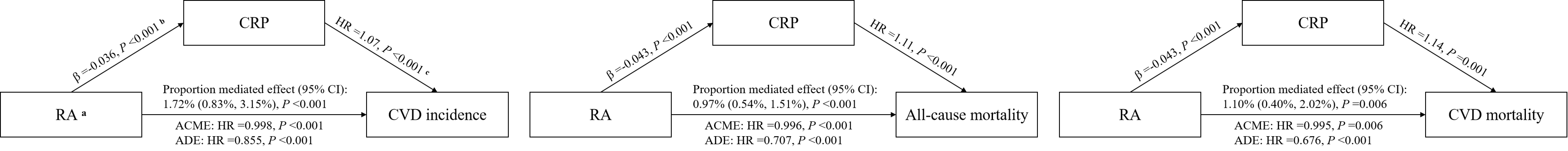


**Figure S18**. Mediation analyses of CRP in the associations of RA with CVD incidence, all-cause and CVD mortality in CKM individuals.

**^a^** Since the continuous variable has a narrow range (1-2), it was scaled by a factor of ten for the analysis. The HR reflects the hazard ratio for a 0.1 unit change in the variable.

**^b^** *P* values from multivariable logistic regression models for major CRAR and inflammation biomarkers.

**^c^** *P* values from multivariable Cox proportional hazards models for inflammation biomarkers and all-cause mortality.

**Abbreviations:** CRP, C-reactive protein; RA, relative amplitude; RDW, red cell distribution width; RAR, red cell distribution width to albumin ratio; HR, hazard ratio; CI, confidence interval; ACME: average causal mediation effect; ADE: average direct effect; CRAR, circadian rest-activity rhythm; CKM, cardiovascular-kidney-metabolic syndrome.


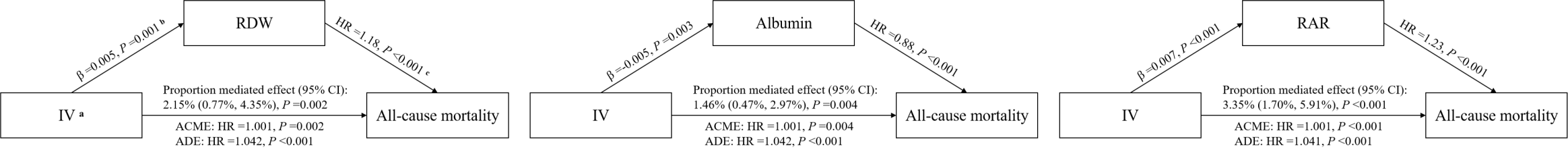


**Figure S19**. Mediation analyses of inflammation biomarkers in the associations of IV with all-cause mortality in patients with CKM stages 1-4.

**^a^** Since the continuous variable has a narrow range (1-2), it was scaled by a factor of ten for the analysis. The HR reflects the hazard ratio for a 0.1 unit change in the variable.

**^b^** *P* values from multivariable logistic regression models for major CRAR and inflammation biomarkers.

**^c^** *P* values from multivariable Cox proportional hazards models for inflammation biomarkers and all-cause mortality.

**Abbreviations:** IV, intradaily variability; RDW, red cell distribution width; RAR, red cell distribution width to albumin ratio; HR, hazard ratio; CI, confidence interval; ACME: average causal mediation effect; ADE: average direct effect; CRAR, circadian rest-activity rhythm; CKM, cardiovascular-kidney-metabolic syndrome.


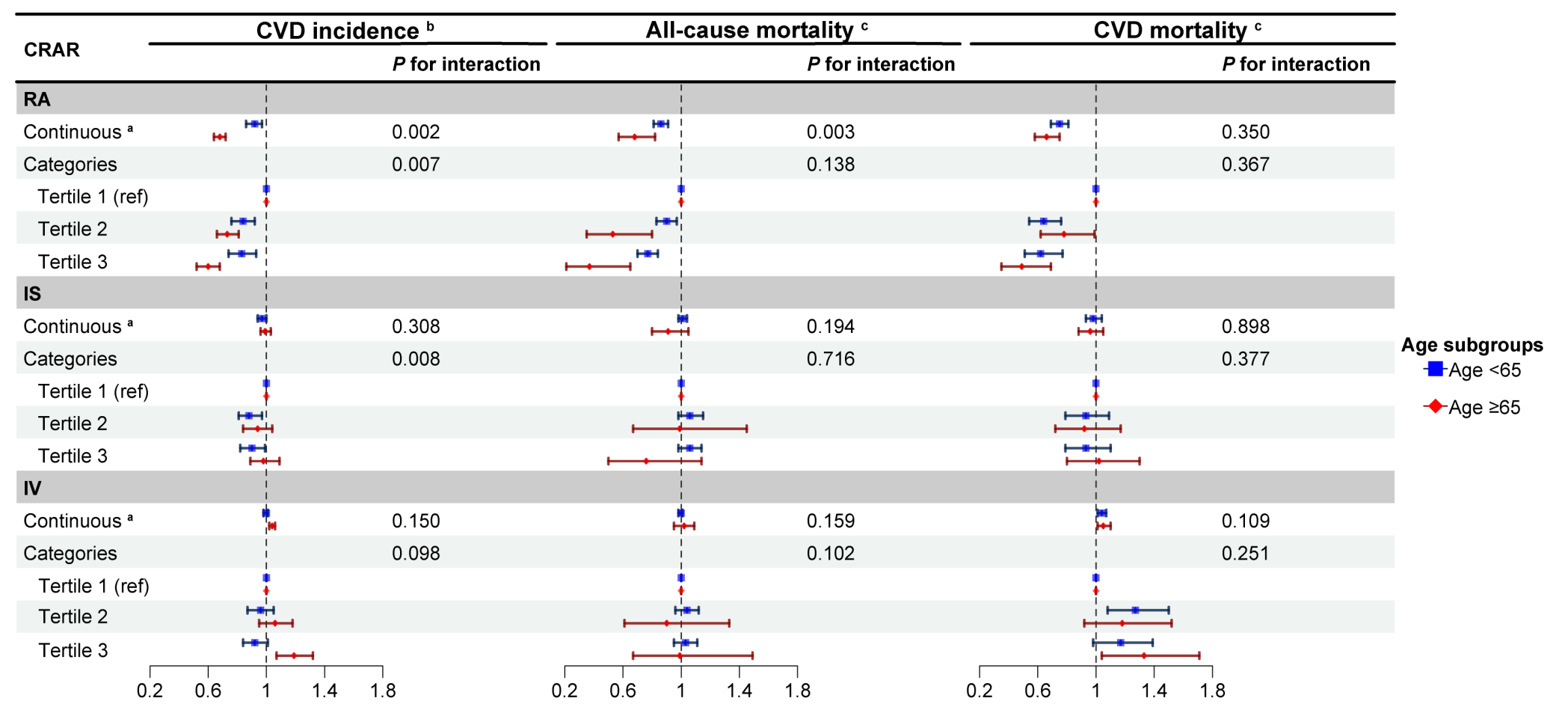


**Figure S20**. Associations between major CRAR and the risk of CVD incidence, all-cause and CVD mortality in CKM individuals, stratified by age.

**^a^** Since the continuous variable has a narrow range (1-2), it was scaled by a factor of ten for the analysis. The HR reflects the hazard ratio for a 0.1 unit change in the variable.

**^b^** The study population for CVD incidence consists of individuals with CKM stages 0-3.

**^c^** The study population for all-cause and CVD mortality consists of patients with CKM stages 1-4.

**Abbreviations:** CRAR, circadian rest-activity rhythm; RA, relative amplitude; IS, interdaily stability; IV, intradaily variability; CVD, cardiovascular disease; CKM, cardiovascular-kidney-metabolic syndrome; HR, hazard ratio.


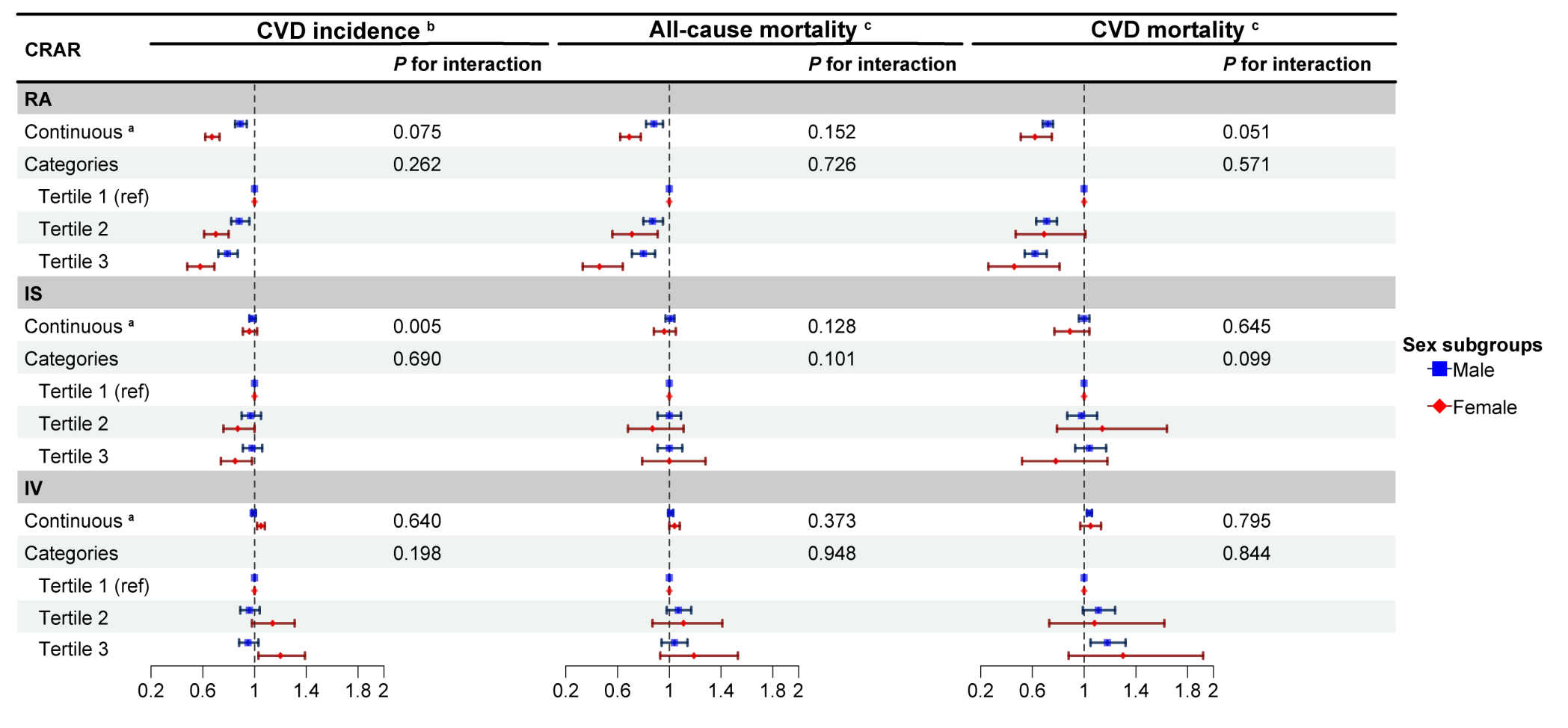


**Figure S21**. Associations between major CRAR and the risk of CVD incidence, all-cause and CVD mortality in CKM individuals, stratified by sex.

**^a^** Since the continuous variable has a narrow range (1-2), it was scaled by a factor of ten for the analysis. The HR reflects the hazard ratio for a 0.1 unit change in the variable.

**^b^** The study population for CVD incidence consists of individuals with CKM stages 0-3.

**^c^** The study population for all-cause and CVD mortality consists of patients with CKM stages 1-4.

**Abbreviations:** CRAR, circadian rest-activity rhythm; RA, relative amplitude; IS, interdaily stability; IV, intradaily variability; CVD, cardiovascular disease; CKM, cardiovascular-kidney-metabolic syndrome; HR, hazard ratio.


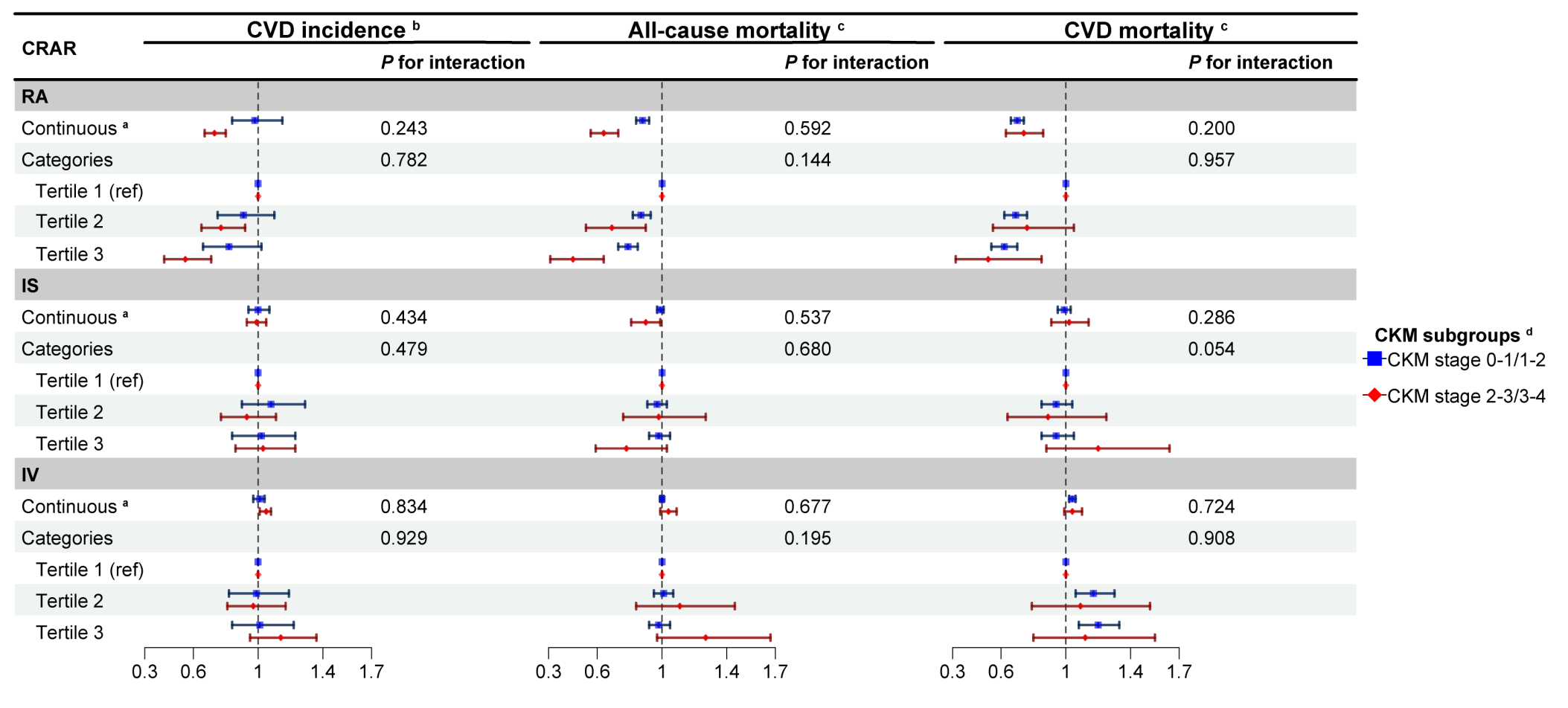


**Figure S22**. Associations between major CRAR and the risk of CVD incidence, all-cause and CVD mortality in CKM individuals, stratified by CKM stages.

**^a^** Since the continuous variable has a narrow range (1-2), it was scaled by a factor of ten for the analysis. The HR reflects the hazard ratio for a 0.1 unit change in the variable.

**^b^** The study population for CVD incidence consists of individuals with CKM stages 0-3.

**^c^** The study population for all-cause and CVD mortality consists of patients with CKM stages 1-4.

**^d^** The stratification for CVD incidence is based on CKM stages 0-1 and 2-3, while the stratification for all-cause and CVD mortality is based on CKM stages 1-2 and 3-4.

**Abbreviations:** CRAR, circadian rest-activity rhythm; RA, relative amplitude; IS, interdaily stability; IV, intradaily variability; CVD, cardiovascular disease; CKM, cardiovascular-kidney-metabolic syndrome; HR, hazard ratio.


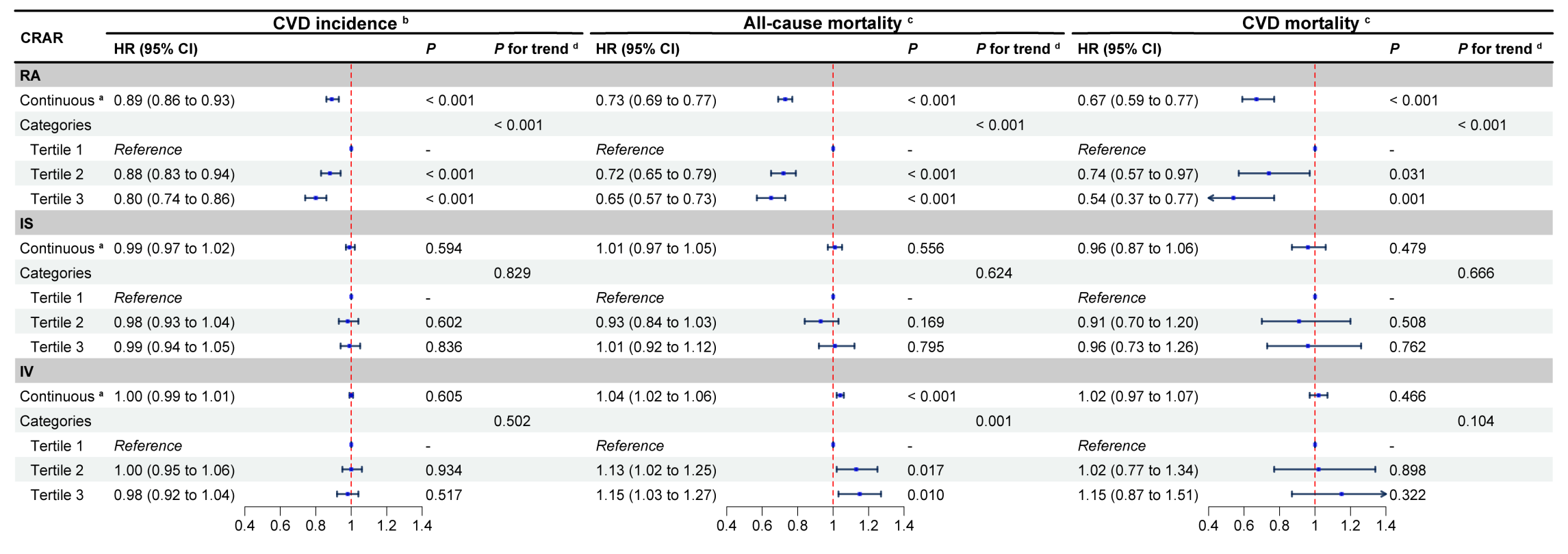


**Figure S23**. Associations between major CRAR and the risk of CVD incidence, all-cause and CVD mortality in CKM individuals, further adjusted for eGFR, depression and cancer.

**^a^** Since the continuous variable has a narrow range (1-2), it was scaled by a factor of ten for the analysis. The HR reflects the hazard ratio for a 0.1 unit change in the variable.

**^b^** The study population for CVD incidence consists of individuals with CKM stages 0-3.

**^c^** The study population for all-cause and CVD mortality consists of patients with CKM stages 1-4.

**^d^** *P* for trend was calculated using multivariable Cox regression models to evaluate the differences in the medians of continuous variables across different tertile groups.

**Abbreviations:** CRAR, circadian rest-activity rhythm; RA, relative amplitude; IS, interdaily stability; IV, intradaily variability; CVD, cardiovascular disease; HR, hazard ratio; CI, confidence interval; CKM, cardiovascular-kidney-metabolic syndrome; eGFR, estimated glomerular filtration rate.


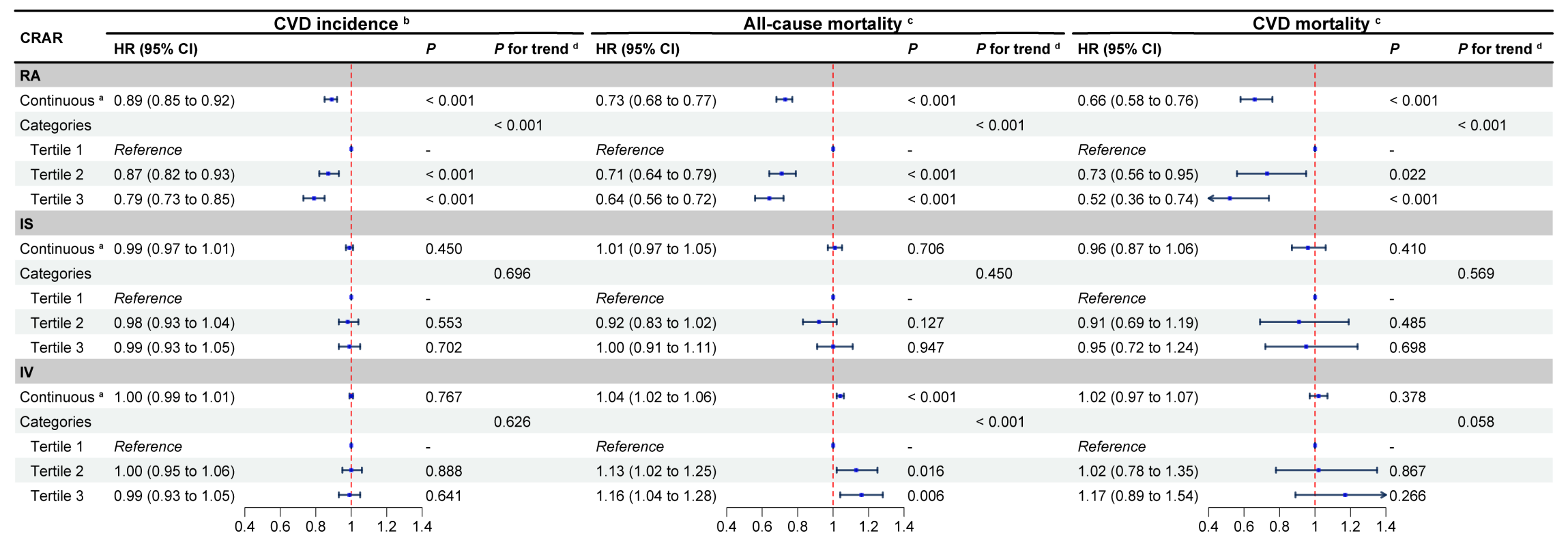


**Figure S24**. Associations between major CRAR and the risk of CVD incidence, all-cause and CVD mortality in CKM individuals, after excluding outcomes occurring within the first two years of follow-up.

**^a^** Since the continuous variable has a narrow range (1-2), it was scaled by a factor of ten for the analysis. The HR reflects the hazard ratio for a 0.1 unit change in the variable.

**^b^** The study population for CVD incidence consists of individuals with CKM stages 0-3.

**^c^** The study population for all-cause and CVD mortality consists of patients with CKM stages 1-4.

**^d^** *P* for trend was calculated using multivariable Cox regression models to evaluate the differences in the medians of continuous variables across different tertile groups.

**Abbreviations:** CRAR, circadian rest-activity rhythm; RA, relative amplitude; IS, interdaily stability; IV, intradaily variability; CVD, cardiovascular disease; HR, hazard ratio; CI, confidence interval; CKM, cardiovascular-kidney-metabolic syndrome; eGFR, estimated glomerular filtration rate.


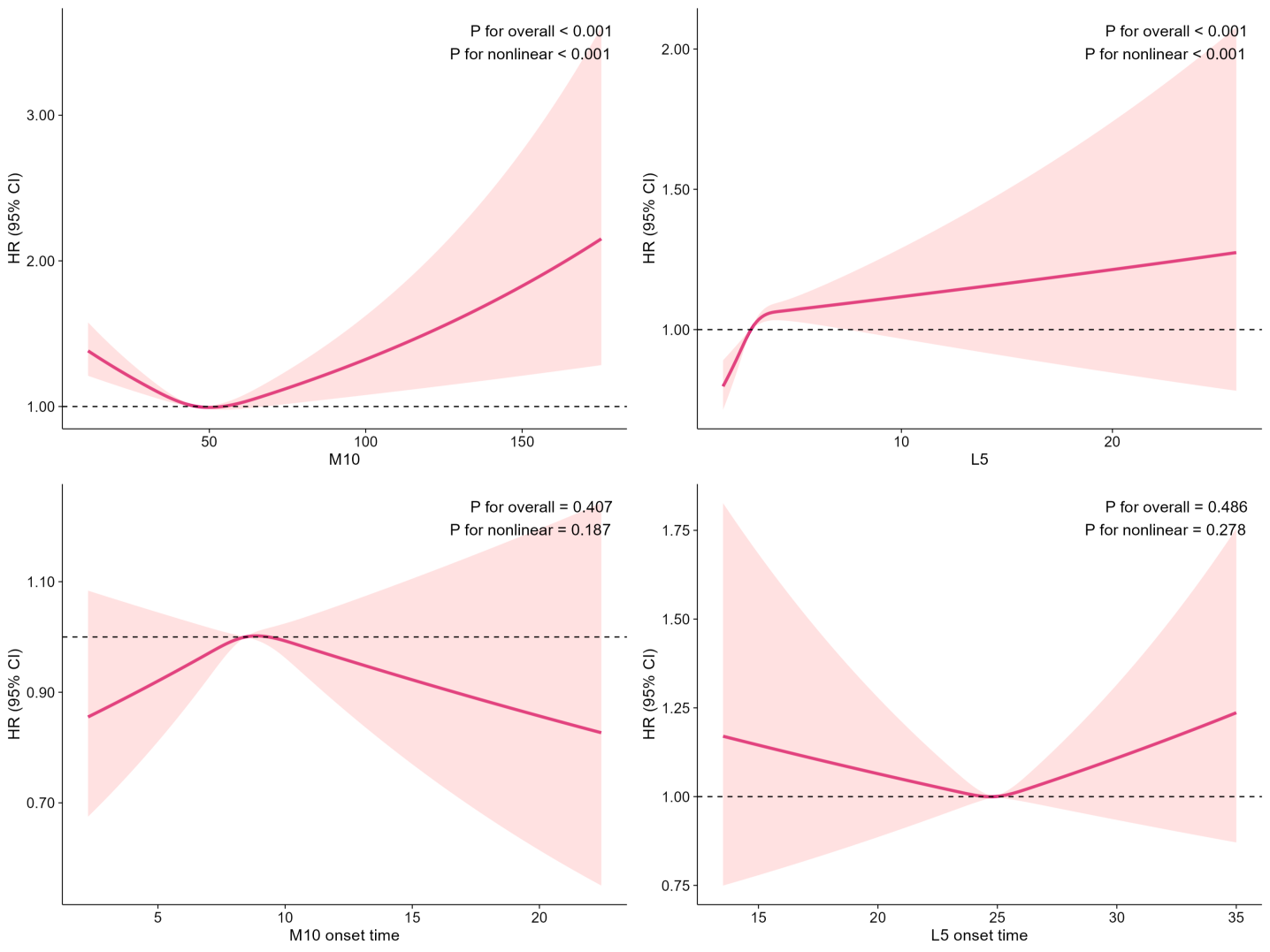


**Figure S25**. Restricted cubic spline analyses for associations between minor CRAR and CVD incidence in individuals with CKM stages 0-3.

*P* values from multivariable Cox proportional hazards models adjusted for age, sex, race, education, TDI, BMI, smoking status, drinking status, healthy diet score, shift work, hypertension, diabetes, hyperlipidemia, MVPA, sleep duration, sleep efficiency, and season of accelerometer wear.

**Abbreviations:** M10, the most active 10-h period; L5, the least active 5-h period; HR, hazard ratio; CI, confidence interval; CRAR, circadian rest-activity rhythm; CVD, cardiovascular disease; CKM, cardiovascular-kidney-metabolic syndrome; TDI, Townsend deprivation index; BMI, body mass index; MVPA, moderate to vigorous physical activity.


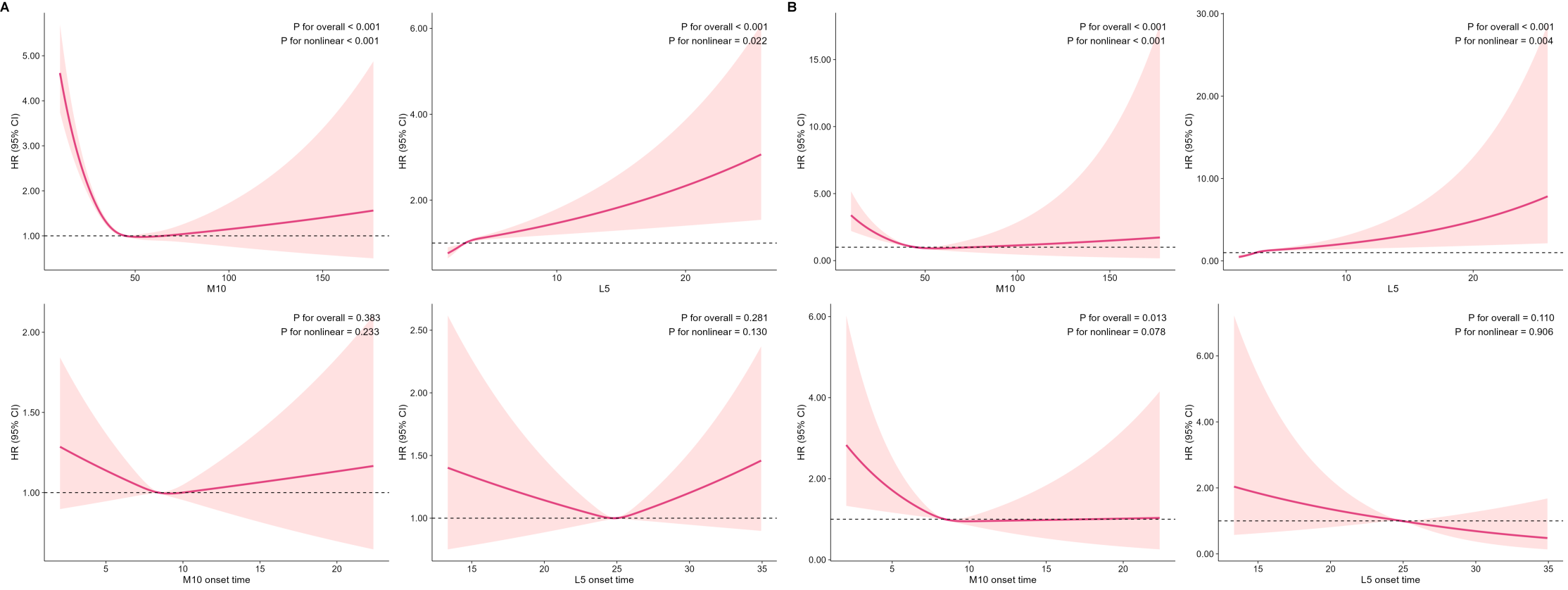


**Figure S26**. Restricted cubic spline analyses for associations between minor CRAR and all-cause and CVD mortality in patients with CKM stages 1-4.

(A) all-cause mortality, (B) CVD mortality.

*P* values from multivariable Cox proportional hazards models adjusted for age, sex, race, education, TDI, BMI, smoking status, drinking status, healthy diet score, shift work, hypertension, diabetes, hyperlipidemia, MVPA, sleep duration, sleep efficiency, and season of accelerometer wear.

**Abbreviations:** M10, the most active 10-h period; L5, the least active 5-h period; HR, hazard ratio; CI, confidence interval; CRAR, circadian rest-activity rhythm; CVD, cardiovascular disease; CKM, cardiovascular-kidney-metabolic syndrome; TDI, Townsend deprivation index; BMI, body mass index; MVPA, moderate to vigorous physical activity.


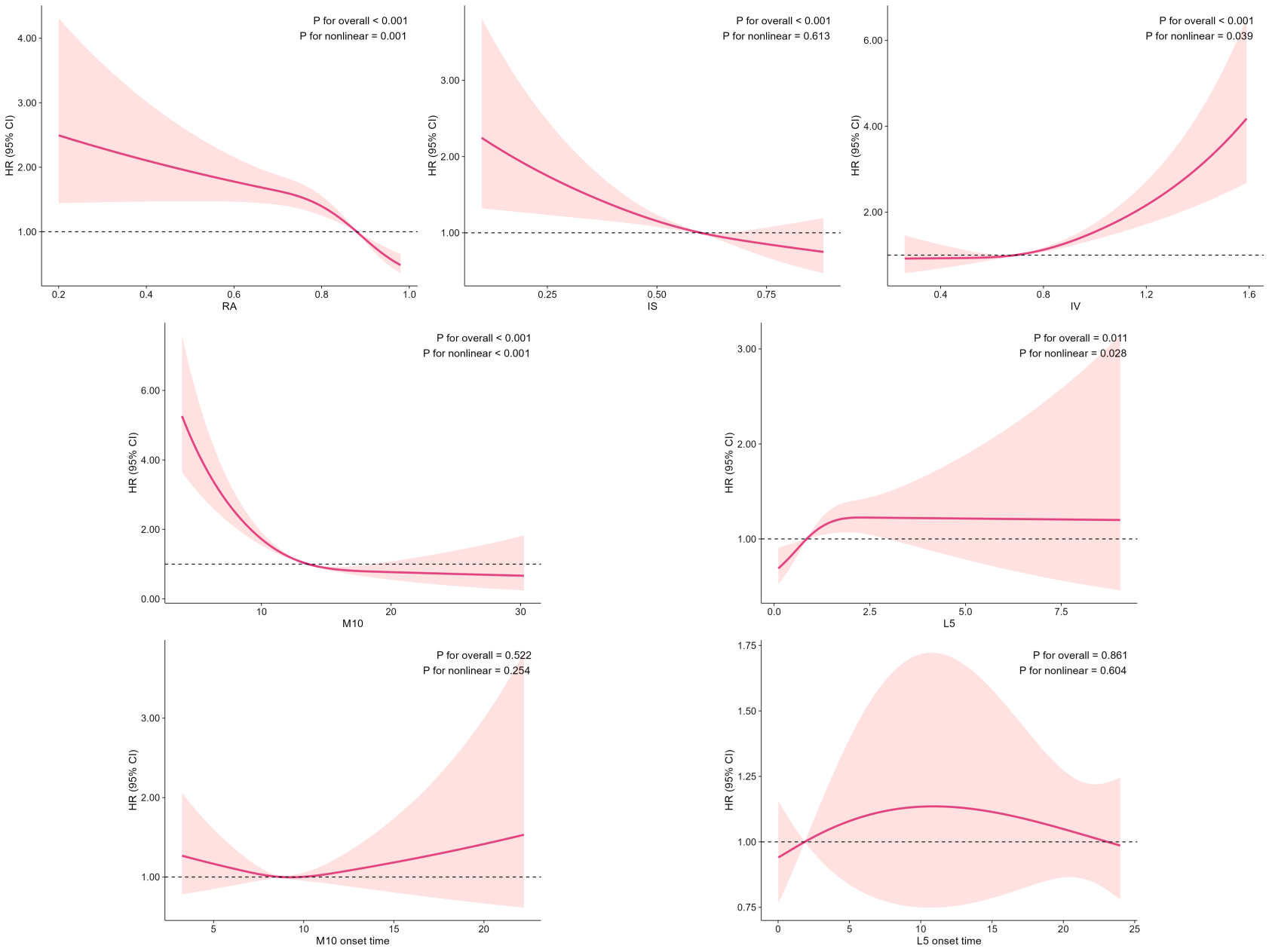


**Figure S27**. Restricted cubic spline analyses for associations between CRAR and all-cause mortality in patients with CKM stages 1-4 from the NHANES.

*P* values from multivariable Cox proportional hazards models adjusted for age, sex, race, education, PIR, BMI, smoking status, drinking status, hypertension, diabetes, hyperlipidemia, MVPA, depression and cancer.

**Abbreviations:** RA, relative amplitude; IS, interdaily stability; IV, intradaily variability; M10, the most active 10-h period; L5, the least active 5-h period; HR, hazard ratio; CI, confidence interval; CRAR, circadian rest-activity rhythm; CKM, cardiovascular-kidney-metabolic syndrome; NHANES, National Health and Nutrition Examination Survey; PIR, poverty income ratio; BMI, body mass index; MVPA, moderate to vigorous physical activity.


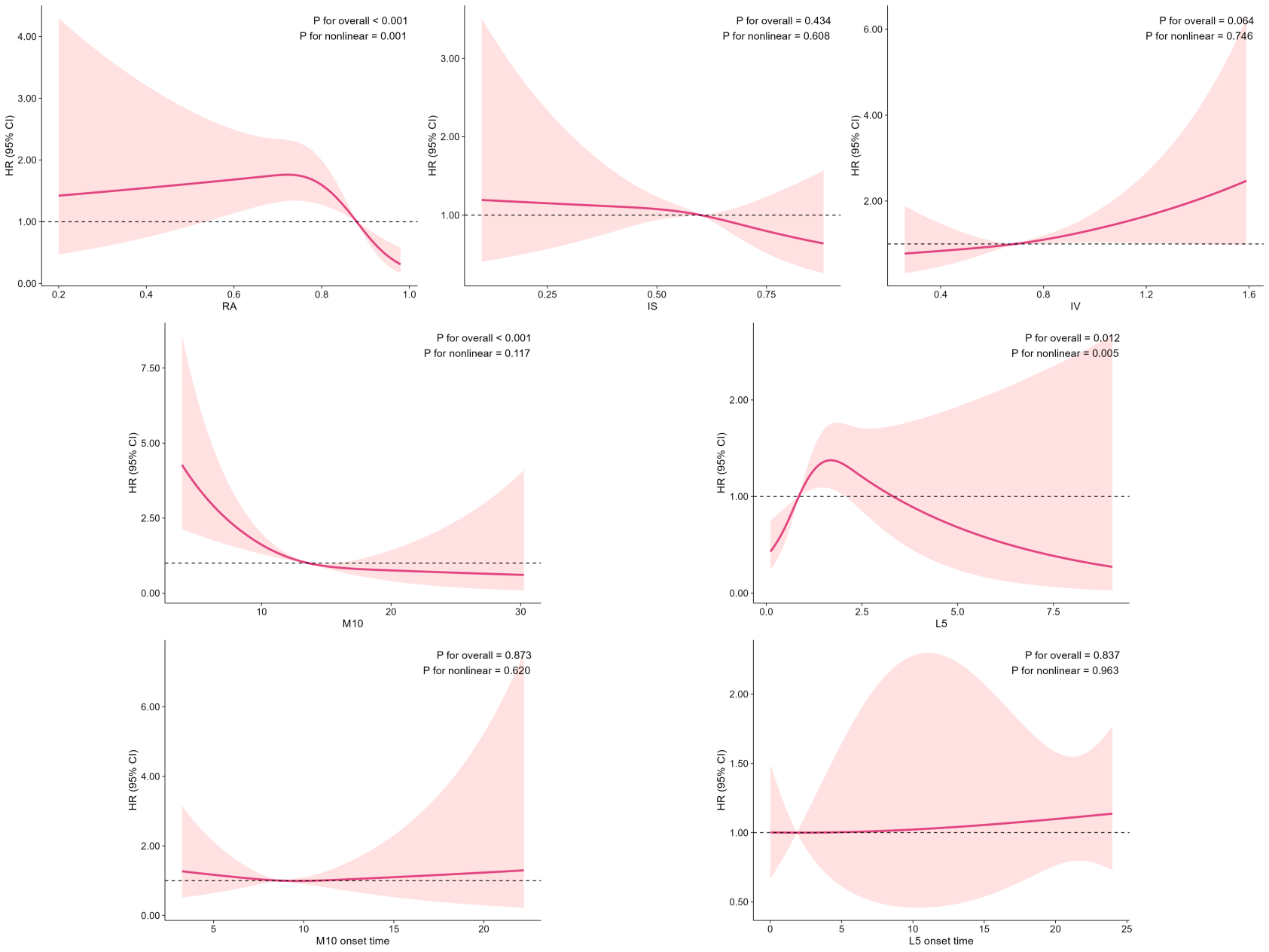


**Figure S28**. Restricted cubic spline analyses for associations between CRAR and CVD mortality in patients with CKM stages 1-4 from the NHANES.

*P* values from multivariable Cox proportional hazards models adjusted for age, sex, race, education, PIR, BMI, smoking status, drinking status, hypertension, diabetes, hyperlipidemia, MVPA, depression and cancer.

**Abbreviations:** RA, relative amplitude; IS, interdaily stability; IV, intradaily variability; M10, the most active 10-h period; L5, the least active 5-h period; HR, hazard ratio; CI, confidence interval; CRAR, circadian rest-activity rhythm; CVD, cardiovascular disease; CKM, cardiovascular-kidney-metabolic syndrome; NHANES, National Health and Nutrition Examination Survey; PIR, poverty income ratio; BMI, body mass index; MVPA, moderate to vigorous physical activity.


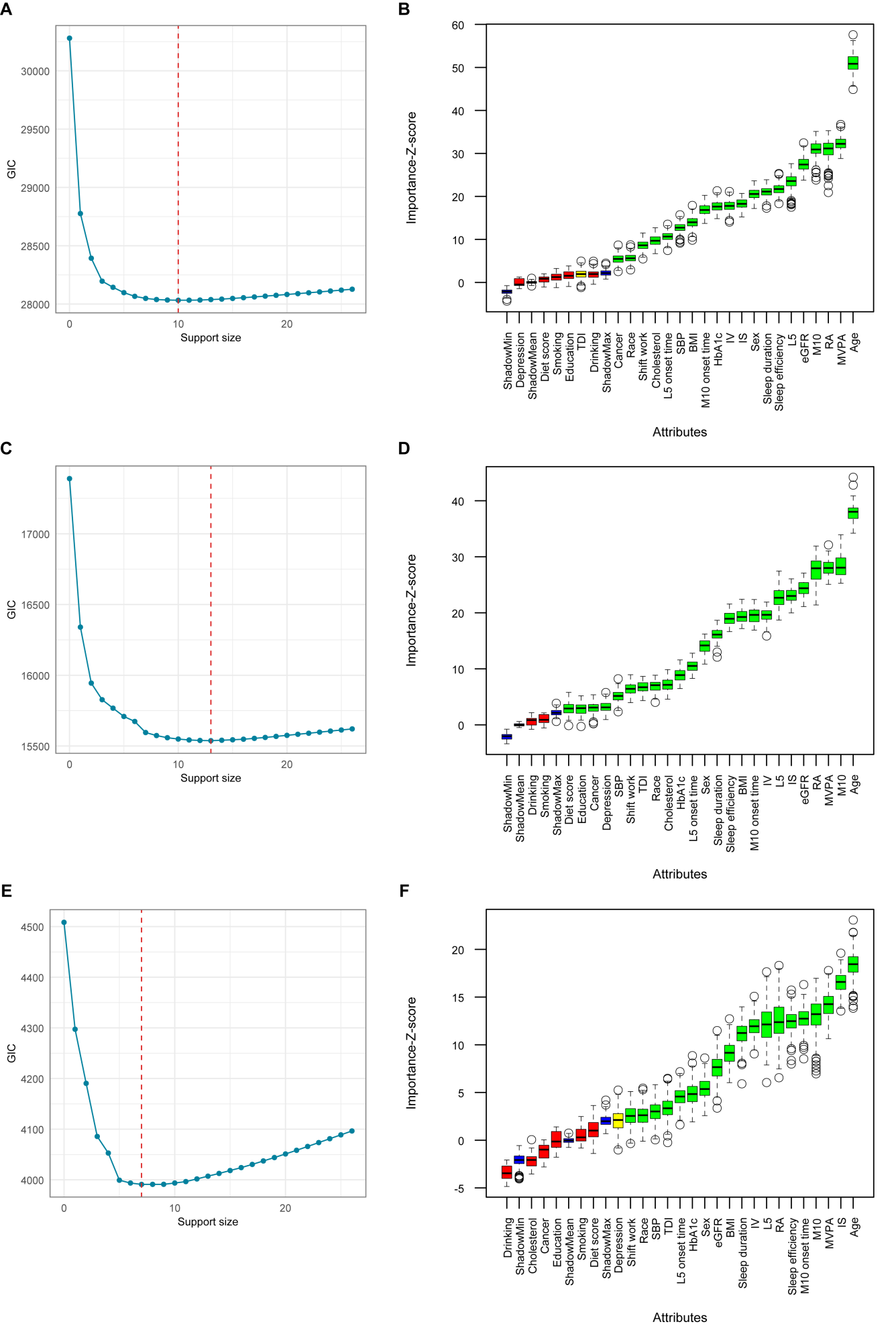


**Figure S29**. Feature selection for CVD incidence, all-cause and CVD mortality using the ABESS and Boruta algorithms.

(A) ABESS-based selection for CVD incidence, (B) Boruta-based selection for CVD incidence, (C) ABESS-based selection for all-cause mortality, (D) Boruta-based selection for all-cause mortality, (E) ABESS-based selection for CVD mortality, (F) Boruta-based selection for CVD mortality.

In the boxplots for B, D, and F, the x-axis represents feature names, and the y-axis represents Z-values. Green boxes indicate selected variables, red boxes rejected, yellow boxes under consideration, and blue boxes shadow variables.

The study population for CVD incidence includes individuals with CKM stages 0-3, while those for all-cause and CVD mortality consist of patients with CKM stages 1-4.

**Abbreviations:** GIC, generalized information criterion; RA, relative amplitude; BMI, body mass index; SBP, systolic blood pressure; HbA1c, glycated hemoglobin; eGFR, estimated glomerular filtration rate; MVPA, moderate to vigorous physical activity; IS, interdaily stability; L5, the least active 5-h period; TDI, Townsend deprivation index; HbA1c, glycated hemoglobin; M10, the most active 10-h period; CVD, cardiovascular disease; CKM, cardiovascular-kidney-metabolic syndrome; ABESS, Adaptive Best Subset Selection.


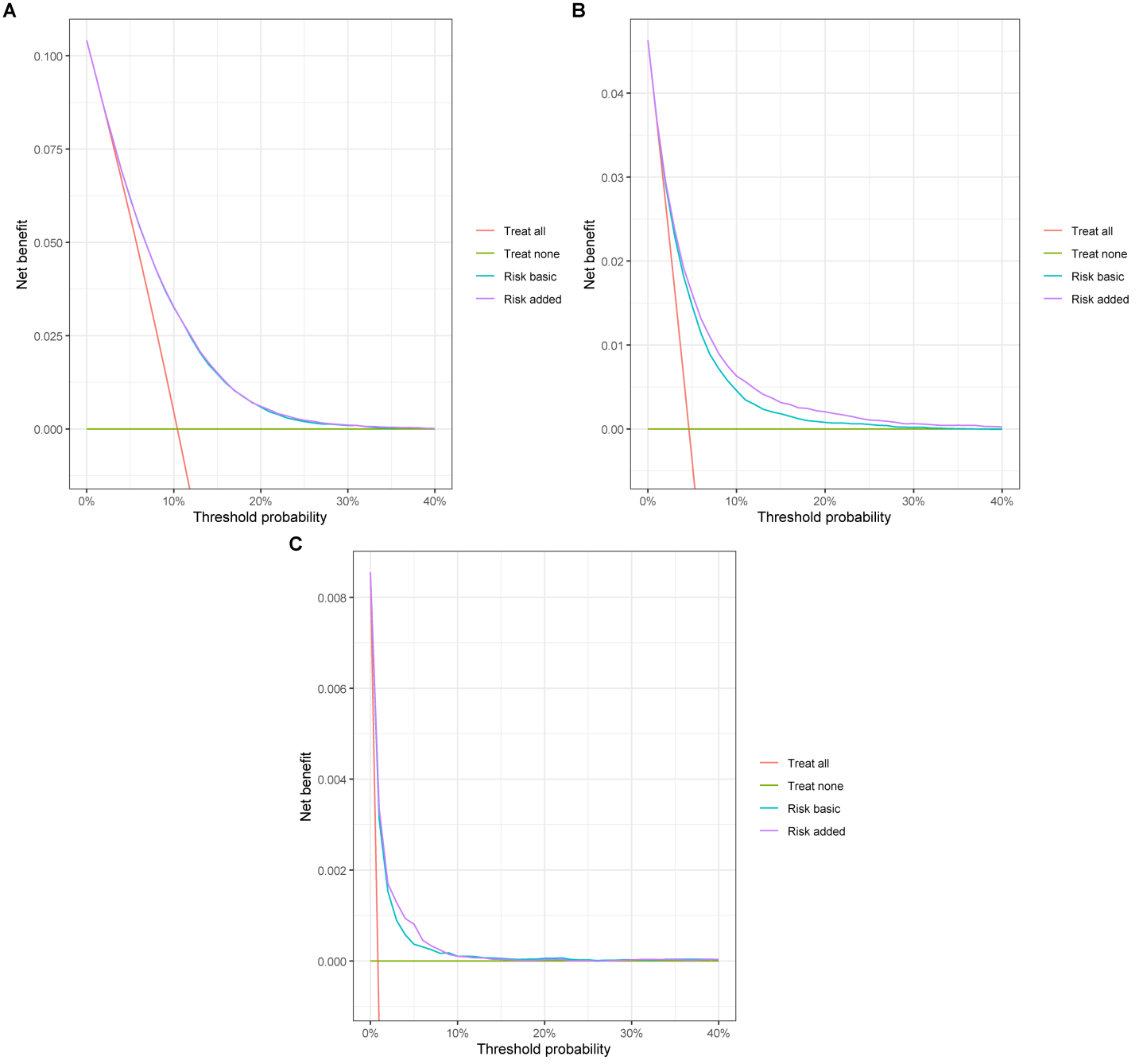


**Figure S30**. Clinical decision curve analyses for basic and incremental prediction models of CVD incidence, all-cause and CVD mortality.

(A) CVD incidence, (B) all-cause mortality, (C) CVD mortality.

The study population for CVD incidence includes individuals with CKM stages 0-3, while those for all-cause and CVD mortality consist of patients with CKM stages 1-4.

**Abbreviations:** CVD, cardiovascular disease; CKM, cardiovascular-kidney-metabolic syndrome.
